# Systemic gene therapy rescues retinal dysfunction and hearing loss in a model of Norrie disease

**DOI:** 10.15252/emmm.202317393

**Published:** 2023-08-29

**Authors:** Valda Pauzuolyte, Aara Patel, James R Wawrzynski, Neil J Ingham, Yeh Chwan Leong, Rajvinder Karda, Maria Bitner‐Glindzicz, Wolfgang Berger, Simon N Waddington, Karen P Steel, Jane C Sowden

**Affiliations:** ^1^ UCL Great Ormond Street Institute of Child Health, University College London London UK; ^2^ NIHR Great Ormond Street Hospital Biomedical Research Centre London UK; ^3^ Wolfson Centre for Age‐Related Diseases, King's College London London UK; ^4^ EGA Institute for Woman's Health, University College London London UK; ^5^ Institute of Medical Molecular Genetics, University of Zürich Zürich Switzerland; ^6^ Zurich Center for Integrative Human Physiology (ZIHP), University of Zürich Zürich Switzerland; ^7^ Neuroscience Center Zurich, University and ETH Zurich, University of Zürich Zürich Switzerland; ^8^ MRC Antiviral Gene Therapy Research Unit, Faculty of Health Sciences University of the Witswatersrand Johannesburg South Africa

**Keywords:** AAV9 gene therapy, cochlea, Ndp, retina, vascular, Genetics, Gene Therapy & Genetic Disease

## Abstract

Deafness affects 5% of the world's population, yet there is a lack of treatments to prevent hearing loss due to genetic causes. Norrie disease is a recessive X‐linked disorder, caused by *NDP* gene mutation. It manifests as blindness at birth and progressive sensorineural hearing loss, leading to debilitating dual sensory deprivation. To develop a gene therapy, we used a Norrie disease mouse model (*Ndp* ^*tm1Wbrg*^), which recapitulates abnormal retinal vascularisation and progressive hearing loss. We delivered human *NDP* cDNA by intravenous injection of adeno‐associated viral vector (AAV)9 at neonatal, juvenile and young adult pathological stages and investigated its therapeutic effects on the retina and cochlea. Neonatal treatment prevented the death of the sensory cochlear hair cells and rescued cochlear disease biomarkers as demonstrated by RNAseq and physiological measurements of auditory function. Retinal vascularisation and electroretinograms were restored to normal by neonatal treatment. Delivery of *NDP* gene therapy after the onset of the degenerative inner ear disease also ameliorated the cochlear pathology, supporting the feasibility of a clinical treatment for progressive hearing loss in people with Norrie disease.

## Graphical abstract

Norrie disease is a genetic condition causing blindness and progressive deafness. Successful AAV‐mediated gene augmentation therapy in a mouse model showed that the Norrie phenotype is responsive to treatment after the onset of degeneration, preventing further progression of hearing loss.

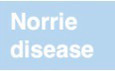

**The paper explained**

**Problem**

Norrie disease is a devastating genetic disorder that causes dual vision and hearing loss in patients without treatment. The study aims to develop a gene therapy for Norrie disease using a mouse model and lay the groundwork for future application in patients.

**Results**

(i) Systemic treatment at an early stage (neonates) resulted in the rescue of vision and hearing, but may not be translatable to humans due to the differences in the development of ears and eyes and the onset of blindness and hearing loss. (ii) Treatment at later stages in mice, equivalent to treatment of children and young adults, was not efficient for rescue of retinal dysfunction, but showed efficacy in significantly improving the outcomes of the progressive hearing deterioration. (iii) Vascular barrier abnormalities in the retina and inner ear were at least partially responsive to treatment across the different stages of the disease.

**Impact**

This study demonstrates that *NDP* gene therapy could be a viable approach to prevent the progression of hearing loss in a genetic deafblindness syndrome, Norrie disease. The efficacy of the therapy after the onset of degenerative changes in the cochlea and in improvement of the vascular barrier in eye and ear strongly supports continuing the effort towards the clinic.

## Introduction

Norrie disease is a rare recessive X‐linked dual sensory disorder, caused by mutations in the Norrie Disease Pseudoglioma (*NDP*) gene and manifests as congenital blindness and progressive hearing loss (Fradkin, 1971; Holmes, 1971; Chen *et al*, 1992; Berger *et al*, 1992a,b). Vision loss is caused by underdevelopment of the deep retinal vasculature, resulting in hypoxia and tractional retinal detachment (Apple *et al*, 1974; Drenser *et al*, 2007). Hearing is usually normal in infants but begins to gradually deteriorate from on average 12 years of age. Hearing loss characteristically begins in one frequency region before spreading to others (Smith *et al*, 2012; Bryant *et al*, 2022). Patients with Norrie disease may also have cognitive impairment, other neurological symptoms and peripheral vascular disease with erectile dysfunction (Rehm *et al*, 1997; Michaelides *et al*, 2004; Smith *et al*, 2012; Cação *et al*, 2018). No curative treatment exists for Norrie disease. However, the delayed onset of hearing loss provides a window of opportunity after diagnosis for early therapeutic intervention that may preserve hearing.

*NDP* encodes norrin (NDP), a secreted soluble WNT analogue, which binds to a receptor complex, consisting of FZD4, LRP5 or LRP6, and TSPAN12, to induce intracellular β‐catenin signalling (Xu *et al*, 2004; Junge *et al*, 2009; Chang *et al*, 2015). This pathway is essential in the eye for the development of the deep retinal vasculature and maintenance of the inner blood–retinal barrier (Apple *et al*, 1974; Xu *et al*, 2004). Hearing loss in Norrie patients has been traced to the cochlea, and both cochlear microvasculature and sensory hair cells are affected (Parving *et al*, 1978; Nadol *et al*, 1990), consistent with norrin signalling being important for development or maintenance of these structures (Rehm *et al*, 2002; Ye *et al*, 2011; Hayashi *et al*, 2021; Bryant *et al*, 2022).

The *Ndp*‐KO mouse model recapitulates human Norrie disease (Nadol *et al*, 1990; Berger *et al*, 1996; Rehm *et al*, 2002). We previously demonstrated that from an early stage in development, these mice have abnormal cochlear vascular morphology and barrier function and a reduction in endocochlear potential. Vascular morphological abnormalities were apparent in the spiral ligament and stria vascularis as early as P10. Loss of cochlear vascular barrier was detected at P20 and reduction of endocochlear potential by 1 month. These changes are followed at the age of 1–2 months by outer hair cell (OHC) degeneration within a discrete “sensitive” tonotopic region associated with corresponding hearing loss in the mid‐frequencies (Bryant *et al*, 2022). This sequence of events implies that auditory dysfunction is directly related to OHC degeneration and that the vascular pathology may be the primary cause of OHC death and hearing loss in Norrie disease (Bryant *et al*, 2022).

To date, no clinical treatments are available to prevent any form of genetic hearing loss or deafblindness. Norrie disease is a good candidate for gene therapy due to its small gene size (coding sequence of 402 bp; Ohlmann & Tamm, 2012). Importantly, targeting *NDP* expression to a specific cell type may not be essential as norrin is secreted, and there is evidence that it does not exert concentration gradient effects or provide directional cues (Ohlmann *et al*, 2005; Wang *et al*, 2012). Adeno‐associated viral (AAV) vectors are favoured for *in vivo* clinical application (Verdoodt *et al*, 2021). AAV9 has been shown to cross the blood–brain barrier (Merkel *et al*, 2017) and transduce a broad range of cells, including in the retina and cochlea (Shibata *et al*, 2017; Massaro *et al*, 2020), and is already approved for clinical use (Mendell *et al*, 2017; Strauss *et al*, 2022).

Considering the multiple sites of pathology in Norrie disease, we investigated the efficacy of an AAV9 vector, carrying a human *NDP* gene therapy construct when delivered intravenously to the *Ndp*‐KO mouse model at three stages of disease progression. We show that early postnatal treatment fully rescued both cochlear and retinal structure and function. Furthermore, treatment of juvenile and young adult mice fully or partially preserved cochlear sensory cells and hearing, while only restoring the blood vessel barrier proteins in the retina. This is the first treatment of Norrie disease phenotype by AAV‐mediated gene therapy and demonstrates amelioration in cochlear pathology and auditory function after treating at clinically relevant stages of Norrie disease progression. It also shows the feasibility in a mouse model of intravenous treatment of deafblindness.

## Results

### Experimental design and function of the *NDP* gene therapy construct *in vitro*

**Figure 1:**
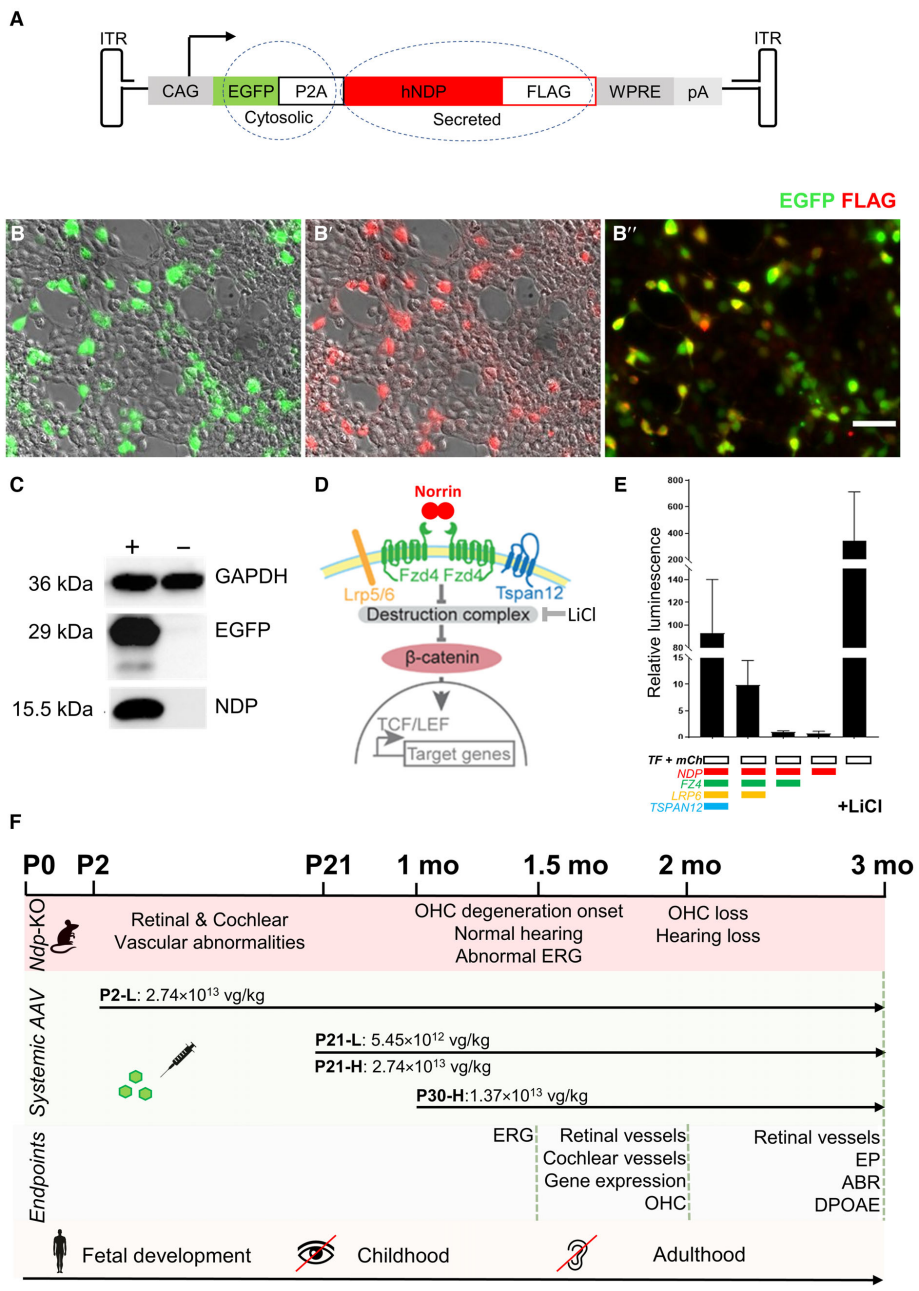
Gene therapy construct evaluation and study design A
Schematic of the AAV9.NDP expression construct. Ubiquitous CAG promoter (cytomegalovirus (CMV) immediate enhancer fused to the chicken beta‐actin promoter) drives expression of the transgene cassette: enhanced green fluorescent protein, EGFP; P2A self‐cleaving linker, human NDP cDNA with a C‐terminal FLAG tag.B‐B″
Expression of AAV9.NDP construct in transfected HEK293 cells. EGFP (green), anti‐FLAG (red), colocalisation (yellow). Scale bar: 50 μm.C
Western blot of transfected (+) and untransfected (−) HEK293 cell lysate. Detection of NDP, GFP and GAPDH proteins (GAPDH provides a loading control; Appendix Fig S1 shows uncropped Western blot images).D
Schematic of the NDP induced β‐catenin signalling. NDP dimer binds to FZ4 complex with essential co‐receptor LRP5 or LRP6 and signal amplifying co‐receptor TSPAN12 and induces β‐catenin binding to TCF/LEF sites in the promoters of the downstream target genes activating transcription.E
TopFlash assay shows activity of transgenic *NDP* in HEK293 cells. AAV9.NDP construct was co‐transfected with FZ4, LRP6, TSPAN12 expression plasmids and TopFlash plasmid encoding firefly luciferase under a promoter containing TCF/LEF binding sites; mCherry plasmid was used as a transfection control. LiCl addition was used as a positive control as it mimics the destruction complex inhibition allowing β‐catenin binding. Luciferase activity was measured as relative luminescence levels; mean ± SD, *N* = 3 technical replicates.F
Experimental design of the treatment administration, endpoints and approximately corresponding stages in human development. Intravenous treatment of the AAV9.NDP virus was administered at P2, P21 or P30 with doses indicated in the schematic. Eye and ear histology in all groups was analysed at 2 months of age; a separate set of mice was analysed for visual function (ERG) at 1.5 months and audiology (DPOAE, ABR) at 3 months of age. Source data are available online for this figure.

To evaluate gene therapy in *Ndp*‐KO mice, we designed an experimental construct to express the human *NDP* gene. It was composed of the strong synthetic CAG promoter upstream of enhanced green fluorescent protein (EGFP; to label the transduced cells), a self‐cleaving P2A linker, the full‐length human NDP coding sequence (including the native secretion signal) with an inserted FLAG epitope sequence at the C terminus (to aid detection of transgenic norrin), followed by a Woodchuck Hepatitis Virus Posttranscriptional Regulatory Element, WPRE sequence and SV40 late poly A sequence at the 3′ end (Fig 1A). Construct expression and function were characterised *in vitro* in HEK293 cells. Fig 1B shows cytoplasmic EGFP (green) in the transfected HEK293 cells that are colabelled with anti‐FLAG (red) immunostaining on the cell surface (co‐localised signal, yellow, Fig 1B‐B″). EGFP and NDP (norrin) protein were detected in Western blots of transfected HEK293 cell lysates (Fig 1C, Appendix Fig S1). Recombinant NDP was detected as a band of NDP‐monomer size (15.5 kDa) with addition of reducing agent β‐mercaptoethanol (Fig 1C, Appendix Fig S1).

A TopFlash luciferase reporter assay in HEK293 cells was used to confirm the competence of the recombinant NDP to activate β‐catenin signalling by interacting with its cognate receptor complex (Fig 1D) (Chang *et al*, 2015). The NDP expression construct induced luciferase activity when cotransfected with human *FZD4*, *LRP6* and *TSPAN12* expression plasmids (Chang *et al*, 2015), but not alone (Fig 1E), consistent with the previously demonstrated NDP interactions with its receptor complex (Chang *et al*, 2015; Lai *et al*, 2017). Addition of lithium chloride, known to stabilise β‐catenin by inhibiting GSK3 (Zeilbeck *et al*, 2014) induced luciferase activity as expected without NDP or its receptors and acted as a positive control. Together, these data indicate that the construct expresses biologically active recombinant NDP.

### Safety and transduction efficiency of eye and ear after systemic delivery of AAV9.NDP

To test the expression of the *NDP* gene therapy construct (EGFP‐P2A‐NDP) in the mouse model, the AAV9 serotype was selected for packaging as it is capable of crossing the blood–brain barrier without transducing the endothelial cells of the blood vessels (Merkel *et al*, 2017). We predicted that the use of the CAG promoter and AAV9 serotype would deliver *NDP* to the retina and cochlea after intravenous injection of recombinant virus (referred to as AAV.NDP) while avoiding iatrogenic damage to the eye or ear by local administration. Based on our previous analysis of *Ndp‐*KO mice (Bryant *et al*, 2022), the goal in the cochlea was to transduce cells close to the blood vessels in the vascularised modiolus and lateral wall so that secreted NDP could target the endothelial cells of adjacent vessels and maintain the microenvironment conducive to sensory hair cell survival (Fig 1F). In the retina, the goal was similarly to transduce cells close to the developing retinal blood vessels so that secreted NDP could potentially prevent retinal vascular malformation.

**Figure 2:**
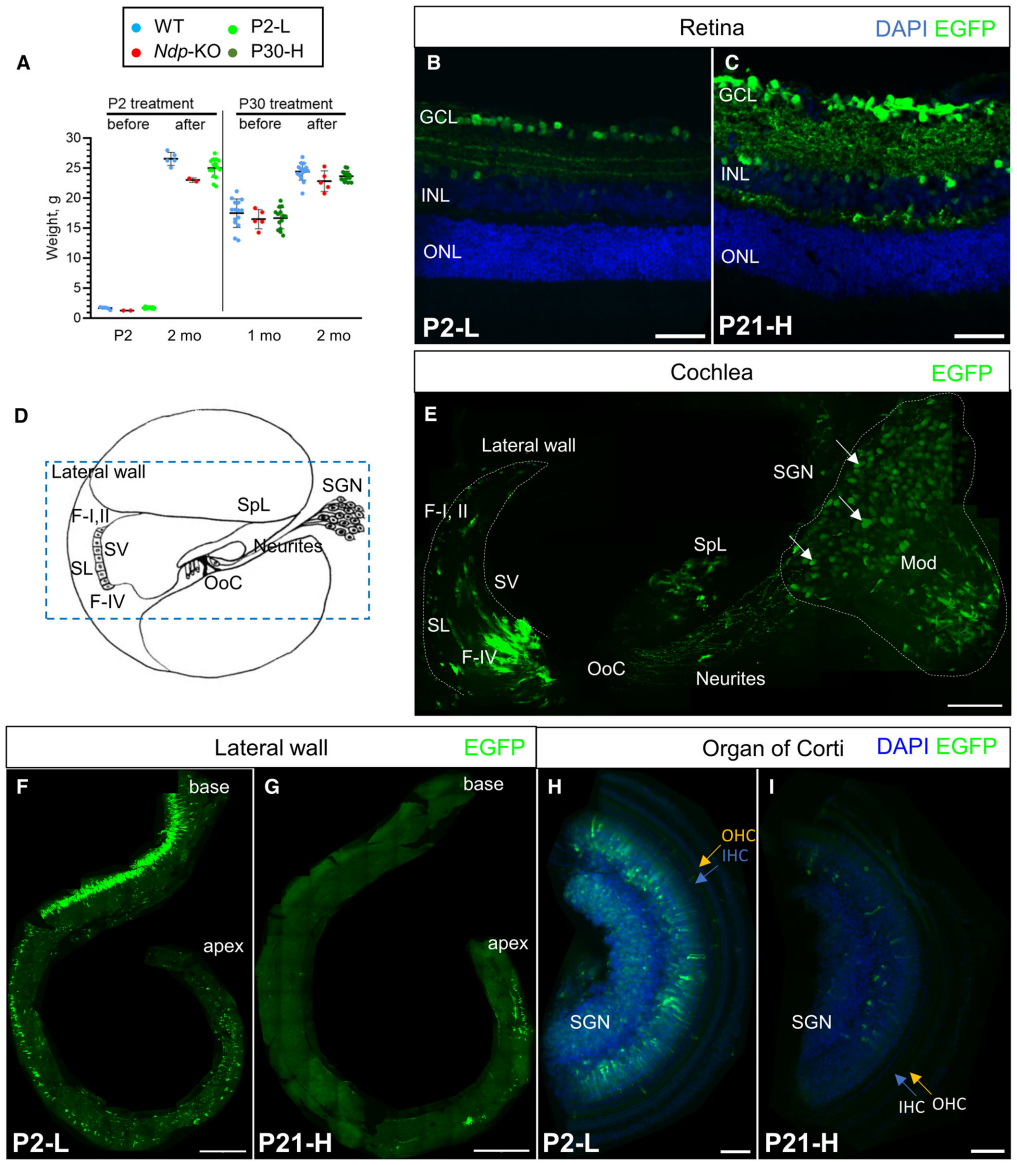
Expression of the construct in eye and ear at 2 months A
Weights of male treated mice and age‐matched litter mate controls before and after AAV9.NDP administration. Data are shown as mean ± SD. Animal numbers: P2‐L group, *n* (WT) = 5, *n* (*Ndp*‐KO) = 2, *n* (P2‐L) = 16; P30‐H group, *n* (WT) = 15, *n* (*Ndp*‐KO) = 5, *n* (P30‐H) = 14.B, C
AAV9.NDP transduction at 2 months in P2 and P21 treated retinal sections: (B) P2‐L group, *n* = 4; (C) P21‐H group, *n* = 4. Staining: anti‐GFP antibody (EGFP, green). GCL – ganglion cell layer, ONL – outer nuclear layer, INL – inner nuclear layer. Scale bar 50 μm.D
A schematic of the axial cross‐section of one turn of the cochlea. SL, spiral ligament; SV, stria vascularis; SGN, spiral ganglion neurons; OoC, organ of Corti; SpL, spiral limbus; F‐I, II, type I and II fibrocytes; F‐IV, type IV fibrocytes. Blue dashed rectangle outlines region, shown in (E). Blue solid rectangle outlines spiral ganglia region.E–I
AAV9.NDP transduction in cochlea: anti‐EGFP antibody (green) and DAPI (blue). Transduction in P2‐treated mouse cochlea at 2 months, cross‐section corresponding to the dashed outline in (D) (E). Transduction of the lateral wall of the cochlea in wholemounts at 2 months after treatment at P2 (F) and P21 (G). Scale bar: 50 μm. Transduction of the organ of Corti in wholemounts at 2 months after treatment at P2 (H) and P30 (I). Scale bar: 100 μm. Appendix Fig S4 shows extended views of cochlear transduction. Source data are available online for this figure.

To test efficacy of AAV9.NDP treatment for progressive hearing loss at timepoints relevant for people with Norrie disease (Bryant *et al*, 2022), three time points were chosen for vector administration, to represent different stages of tissue maturation and pathology stages in the eye and cochlea (Fig 1F): (i) neonatal (postnatal day, P2): before the onset of vision and hearing; at the commencement of retinal vascular formation and before establishment of the endocochlear potential; (ii) juvenile, pre‐degenerative (P21): the retinal and cochlear vasculature have recently matured; no hair cell death has yet occurred; (iii) juvenile, degenerative (P30, referred to as young adult); at the onset of progressive hair cell death in the cochlea; neovascularisation is present in the eye. These treatment timepoints correspond to human development as before birth (mid gestation), in childhood and in young adults. In patients with Norrie disease, severe retinal vascular abnormality and consequent retinal detachment are present at birth; however, hearing is normal at birth and hearing loss progresses from childhood into adulthood (Smith *et al*, 2012). In untreated *Ndp*‐KO mice, the development of the superficial retinal vasculature is slower than normal, but is completed by P20; however, the deep layers fail to form (Richter *et al*, 1998; Luhmann *et al*, 2005a); Appendix Fig S2A–F shows the status of the *Ndp*‐KO retinal vasculature at the P2 and P21 treatment time points and the onset of hair cell death in the cochlea at the P30 treatment timepoint. Fig 1F summarises the study design. AAV9.NDP was delivered by intravenous injection to groups of *Ndp*‐KO neonatal mice (2.74 × 10^13^ viral genomes per kilogramme body weight, vg/kg; dose P2‐L), juvenile mice at P21 at one of two doses (5.45 × 10^12^ vg/kg; dose P21‐L and 2.74 × 10^13^ vg/kg; P21‐H) and young adults at P30 (1.37 × 10^13^ vg/kg; dose P30‐H). AAV9.NDP treated *Ndp*‐KO mice were monitored periodically and were of normal weight and similar general health as compared to WT and untreated *Ndp*‐KO controls (Fig 2A, Appendix Fig S2G and H).

**Figure EV1:**
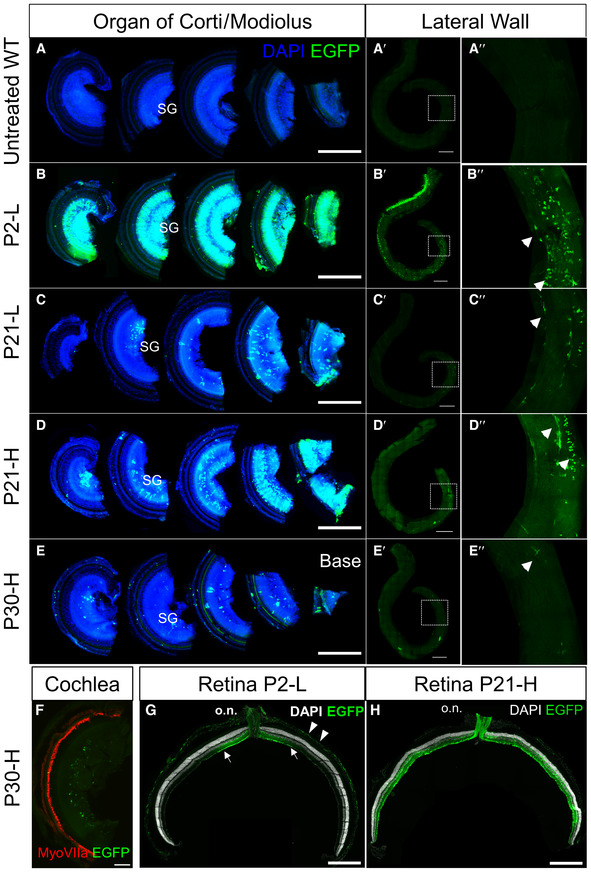
AAV9.NDP vector transduction in the cochlea and retina A–E
Organ of Corti and lateral wall wholemounts stained with anti‐GFP antibody. (A‐A″) Untreated WT cochlea; (B‐B″) P2‐L dose; (C‐C″) P21‐L dose; (D‐D″) P21‐H dose; (E‐E″) P30‐H dose. Scale bars = 500 μm (A‐E), 500 μm (A′‐E′), enlarged view of boxed region shown in A″‐E″. SGN, spiral ganglia region; arrowheads, fibrocyte shaped cells in the lateral wall.F
P30‐H dose; cochlea stained with anti‐MyoVIIa antibody showing that hair cells are not transduced. Scale bar 100 μm.G, H
EGFP immunostaining at 2 months in retinal cryosections after AAV9.NDP treatment at P2 (G) and P21 (H); optic nerve: o.n. Arrows indicate transduced region of the retina; arrowheads indicate transduced cells in the RPE. Scale bar 500 μm. Source data are available online for this figure.

At 2 months of age, transduction of the retina and cochlea was confirmed by EGFP immunostaining (Figs 2 and EV1). Retinas of P2‐injected mice were most efficiently transduced in the central region, which reflects the region of retina thus far vascularised at P2 (Figs 2B and EV1G). Administration at P21, by which time the vasculature covers the entire inner retinal surface in the *Ndp‐*KO mice, resulted in widespread transduction (Figs 2C and EV1H). Retinal ganglion cells were efficiently transduced in early or late treated mice, whereas expression in Müller glial cells, a physiological site of *Ndp* expression (Ye *et al*, 2009), was rare (Fig 2B and C).

In the cochlea, transduction was achieved in the modiolus and lateral wall near to the blood vessels and putative targets of NDP signalling (Rehm *et al*, 2002; Hayashi *et al*, 2021; Bryant *et al*, 2022) (Fig 2D and E). The spiral ganglia region was transduced as well as the lateral wall and modiolus (Figs 2F–I and EV1A–E). Transduction appeared higher after neonatal administration compared with treatment in juveniles and young adults (Figs 2F–I and EV1A–E). GFP labelling showed that lateral wall transduction was efficient in the P2‐L and P21‐H group, but not in the P30‐H group (Fig EV1). No transduction was observed in the outer hair cells and vascular endothelial cells (Fig EV1F).

**Figure 3:**
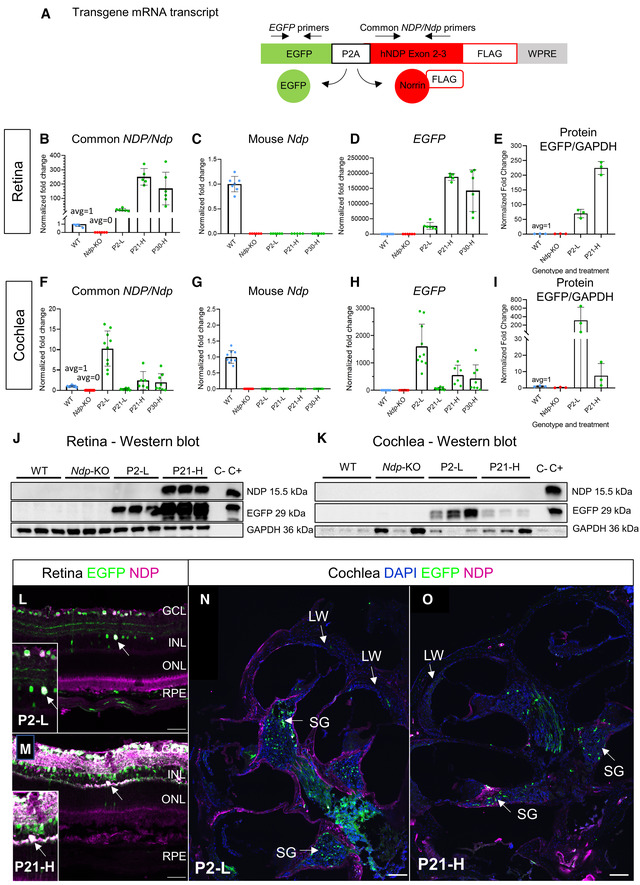
Efficacy of construct expression in the eye and ear A
Schematic of the human NDP gene therapy construct. Primers specific to the EGFP sequence detected construct derived EGFP‐P2A‐NDP mRNA. The ORF of the NDP/Ndp gene spans exons 2 and 3. Primers to conserved sequence common to the human *NDP* gene therapy construct and the mouse mRNA were designed to span intron 2, allowing the comparison of levels of endogenous or construct derived mRNA (Common *NDP/Ndp* primers).B–D
qRT‐PCR analysis of levels of the gene therapy construct EGFP‐P2A‐NDP mRNA and endogenous *Ndp* and at 2 months in the retina in all treatment groups. Common *NDP*/*Ndp* primers (B), mouse *Ndp* primers (C), EGFP primers (D). Highest *NDP* and *EGFP* expressions were detected in the P21‐H group in retina and the P2‐L group in cochlea. No amplicon was obtained from *Ndp*‐KO treated groups or *Ndp*‐KO controls using primers specific to the mouse *Ndp* sequence. Data are shown as mean ± SD; each data point represents a biological replicate.E
Analysis of western blots of retina at 2 months. Levels of EGFP relative to GAPDH were quantified. Data are shown as mean ± SD; each data point represents a biological replicate.F–H
qRT‐PCR analysis of levels of the gene therapy construct EGFP‐P2A‐NDP mRNA and endogenous *Ndp* and at 2 months in the cochlea in all treatment groups. Common *NDP*/*Ndp* primers (F), mouse *Ndp* primers (G), EGFP primers (H). Highest *NDP* and *EGFP* expressions were detected in the P21‐H group in retina and in the P2‐L group in cochlea. No amplicon was obtained from *Ndp*‐KO treated groups or *Ndp*‐KO controls using primers specific to the mouse *Ndp* sequence. Data are shown as mean ± SD; each data point represents a biological replicate.I
Analysis of Western blots of cochlea at 3 months. Levels of EGFP relative to GAPDH were quantified (E, I). Data are shown as mean ± SD; each data point represents a biological replicate.J, K
Western blots from all treatment groups used for analyses in E, I showing NDP protein was detected in the P21‐H treatment group in retina but was below detectable levels in the P2‐L group (J) and was not detectable in cochlea samples (K). Transfected (+) and untransfected (−) HEK293 cell lysate was used as a control. NDP protein was not detected in WT retina or cochlea by Western blot.L, M
Immunostaining analysis of AAV9.NDP transduction in retinal sections at 2 months after P2 and P21 treatments. GCL, ganglion cell layer; INL, inner nuclear layer; ONL, outer nuclear layer; RPE, retinal pigment epithelium. Scale bar 50 μm. Anti‐GFP antibody (EGFP, green); anti‐NDP antibody; (magenta), colocalisation signal is white; DAPI (blue). Figure 3L and M reused in EV2 DAPI/P21‐H and EGFP/P2‐L.N, O
Immunostaining analysis of AAV9.NDP transduction in cochlea sections at 3 months after P2 and P21 treatments. Scale bar 100 μm. SG, spiral ganglia region; LW, lateral wall. Anti‐GFP antibody (EGFP, green); anti‐NDP antibody (magenta); colocalisation signal is white; DAPI (blue). Fig 3L and M reused in EV2 DAPI/P21‐H and EGFP/P2‐L. Source data are available online for this figure.

To assess the efficiency of transgene expression in treated mice after early (P2) and later (P21‐P30) AAV9.NDP treatment, retina and cochlea samples were analysed by qRT‐PCR (at 2 months of age) and Western blot (at 3 months of age). We employed an EGFP‐P2A‐NDP construct in our study design (Fig 3A) (which produces both EGFP and NDP proteins from a single EGFP‐P2A‐NDP transgene mRNA) as previously we were not able to detect NDP protein by immunohistochemistry or Western blotting analysis of WT mouse tissue. Primers complementary to coding sequences conserved between human *NDP* and mouse *Ndp* in exons 2 and 3 (Fig 3A, *Ndp/NDP* primers) were designed to compare the levels of the transgene mRNA with endogenous mouse *Ndp* mRNA expression levels (Fig 3B and F). Mouse *Ndp‐*specific primers confirmed absence of *Ndp* expression in *Ndp*‐KO and the treated mice compared to presence in the WT (Fig 3C and G). Primers specific to EGFP sequence in the transgene mRNA were also used to confirm EGFP‐P2A‐NDP transgene expression levels (Fig 3D and H).

In the retina of treated *Ndp*‐KO mice, human *NDP* mRNA levels were 20‐fold higher in the P2‐L treatment group, and over 250‐ and 168‐fold higher in the retinas of the P21‐H and P30‐H groups, respectively, compared to endogenous *Ndp* expression in WT mice (Fig 3B). Western blot analysis confirmed lower levels of EGFP protein in the P2‐L treated group compared to the P21‐H group (Fig 3E and J).

In the cochlea, *NDP* mRNA levels were 10‐fold higher expression in the P2‐L group and 2.4‐fold and 1.9‐fold, respectively, in the P21‐H and P30‐H groups, with negligible expression in the P21‐L group, compared to endogenous *Ndp* expression in the WT (Fig 3F). Similarly, the levels of the transgene mRNA in the cochlea, detected with *EGFP*‐specific primers, were highest after treatment in neonates (see P2‐L and P21‐H groups, injected with the same amount by vg/kg; Fig 3H). In juveniles and young adults, expression corresponded to AAV9.NDP dosage with higher expression at P21‐H and P30‐H groups compared with the low‐dose treatment at P21‐L (Fig 3H). These patterns are in line with the patterns of GFP transduction observed in cochlear whole mounts (Fig EV1) indicating dependence of transduction levels on age and dose. Western blot analysis confirmed a higher level of EGFP protein band in the P2‐L treated cochlea compared to P21‐H (Fig 3I–K).

We also attempted NDP protein analyses; a 15.5‐kDa band corresponding to NDP protein was detected in the P21‐H retina samples only (Fig 3J) and not in WT and P2‐L treatment groups. NDP protein could not be detected by Western blot in cochlea samples (Fig 3K); NDP is known to bind extracellular matrix and form disulphide‐bridged oligomers (Perez‐Vilar & Hill, 1997) which may impede isolation of already low levels of NDP.

Together, these data show transduction of both neonatal, juvenile and young adult cochlea and retina was achieved by the AAV9.NDP vector. Western blot assays detected EGFP protein levels consistent with qRT‐PCR and immunohistochemistry results. Transduction levels at 2 months appeared highest in the cochlea after treatment at P2 and highest in the retina after treatment at P21.

### Transgenic norrin was detected by immunostaining in the retina and cochlea of treated mice

Although the secreted NDP proved technically difficult to immunostain, we developed a new protocol which detected recombinant norrin protein in 2‐month retina and 3‐month cochlea cryosections of *Ndp*‐KO mice from the P2‐L and P21‐H treatment groups (Fig 3L–O; Appendix Figs S3 and S4).

In the retina, after AAV9‐NDP treatment at P2 and P21 (Fig 3L and M), anti‐NDP immunostaining showed cytoplasmic NDP signal (magenta) coinciding with, though less widespread than anti‐EGFP staining (co‐localised signal, white) in cells in the ganglion cell and inner nuclear layers (Appendix Fig S3A–D shows the separate anti‐NDP and GFP fluorescent channels). Rarely, photoreceptor cells and Müller cells were transduced.

In the cochlea, anti‐NDP extracellular immunostaining was observed along the walls of the hollow cochlear chambers, whereas cytoplasmic GFP localised predominantly in the modiolus (Fig 3N and O) after AAV9‐NDP treatment at P2 and P21 (separate anti‐NDP and GFP fluorescent channels shown in Appendix Fig S4). Co‐staining for EGFP with TUBB3 (neuronal marker; Appendix Fig S4I) and with GFAP (glial marker; Appendix Fig S4I′) in P2‐L cochlea showed that AAV9‐NDP transduced spiral ganglion neurons.

Based on the analysis of an alkaline phosphatase reporter *Ndp* ^*AP*^ mouse model (Ye *et al*, 2011), *Ndp* expression is reported in the postnatal mouse retina and in the lateral wall and modiolus of the postnatal and adult mouse cochlea. NDP protein was recently reported to localise to the inner sulcus of the organ of Corti (Hayashi *et al*, 2021). To better elucidate the expression of endogenous *Ndp* in the WT retina and cochlea, to compare it with the AAV‐mediated *NDP* expression, we analysed recently generated publicly available scRNA sequencing datasets (Heng *et al*, 2019; Milon *et al*, 2021; Dong *et al*, 2022). *Ndp* expression was detected in Müller glial cells and horizontal cells in the P11 (Appendix Fig S5A and B) and adult mouse retina (Appendix Fig S5C and D). In the adult mouse cochlea, *Ndp* expression was detected in glia/Schwann cell clusters in the spiral ganglia region (Appendix Fig S5E and F) and basal cells and fibrocytes in the lateral wall (Appendix Fig S5G and H). Datasets EV1 and EV2 show the top marker genes identifying each cell cluster. Our EGFP/NDP immunostaining in the spiral ganglia region of the cochlea of treated mice labelled mainly neuronal rather than glial cells (Appendix Fig S4). In the lateral wall, few EGFP cells were detected by anti‐NDP immunostaining on cryosections (Appendix Fig S4). These analyses suggest that there are differences in the cell types expressing AAV‐GFP‐P2A‐NDP and the endogenous *Ndp* gene in the modiolus and lateral wall. However, secreted recombinant NDP protein localised in both these target regions of the cochlea.

### Neonatal treatment with AAV9.NDP rescues retinal vasculature and visual function

**Figure 4:**
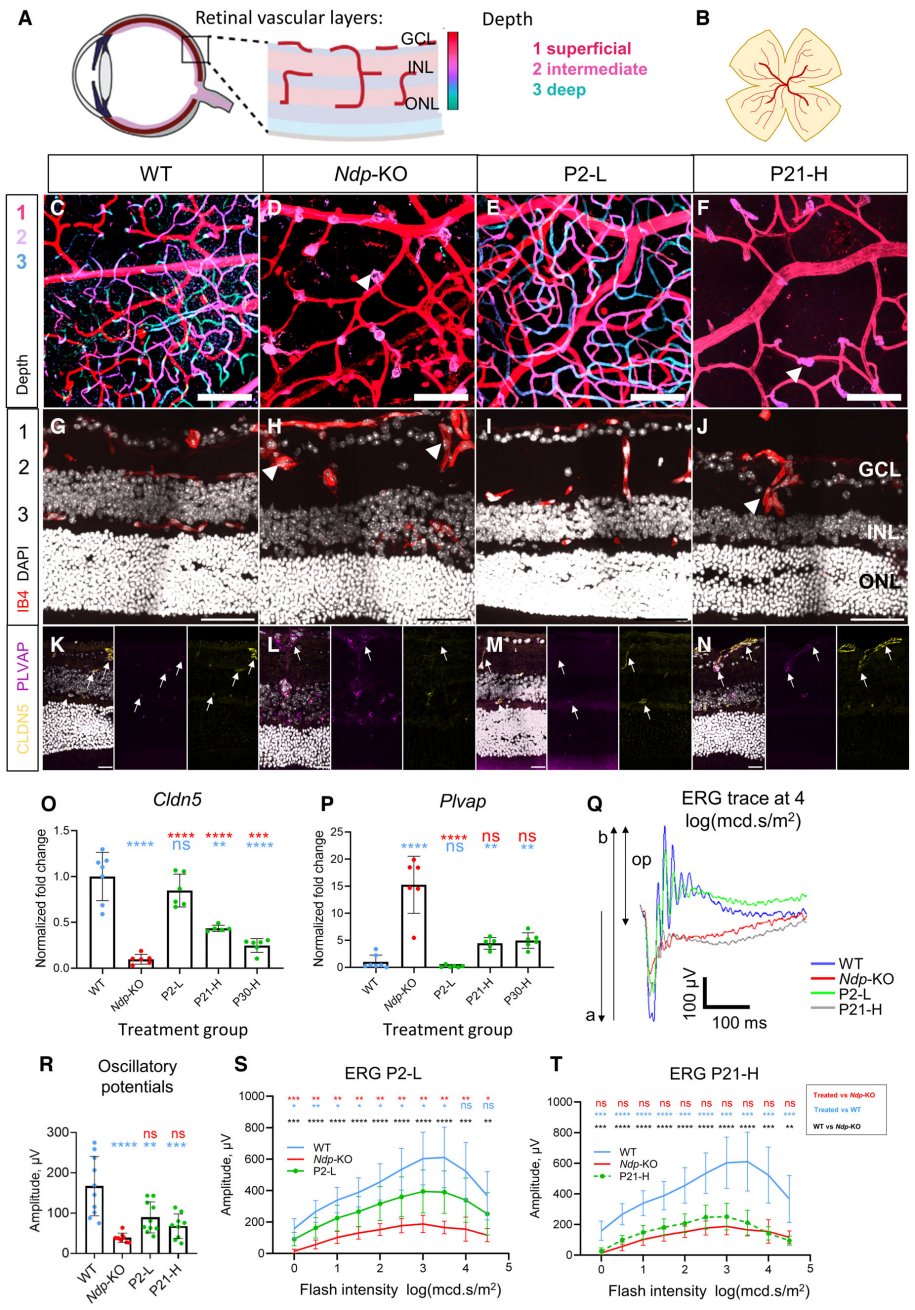
Effects of AAV9.NDP treatment on the retinal vessel morphology and visual function A, B
Schematic of the normal retinal vasculature showing three vascular plexi in (A) cross‐sections and (B) whole mount, colour depth projection scheme indicated for (C–F).C–F
Pseudocoloured depth projections of vascular plexi in the central retina of retinal whole mounts at 3 months. Vasculature staining with isolectin B4 (IB4) shows three plexi in the WT and after treatment at P2. C–F images are z‐projections of 50 slices of 0.75 μm. Treatment groups: C, WT; D, *Ndp*‐KO; E, P2‐L; F, P30‐H.G–J
Vasculature: IB4, nuclei: DAPI at 2 months.K–N
Localisation of tight junction marker claudin‐5 (CLDN5) and PLVAP at 2 months.O, P
qRT‐PCR analysis of pathology‐related genes (O) *Cldn5* (P) *Plvap* in retina at 2 months. Data information: Genotype and treatment ages (WT, *Ndp*‐KO, P2‐L, P21‐L, P21‐H, P30‐H) indicated on *x* axes. Mean ± SD; *n* = biological replicates. Sample numbers: *n* (WT) = 7, *n* (*Ndp*‐KO) = 6, *n* (P2‐L) = 6, *n* (P21‐L) = 6, *n* (P21‐H) = 6, *n* (P30‐H) = 6. Statistical analysis: analysed with one‐way ANOVA with Sidak's *post hoc* test, all values compared to WT (blue asterisks) and *Ndp*‐KO (red asterisks).Q–T
Evaluation of visual function recovery with scotopic electroretinography (ERG) at 1.5 months of age. Example of ERG waves at 10 cd/s.m^2^ flash intensity stimulus in WT, *Ndp*‐KO, P2‐L and P21‐H groups, *n* = 1 (average of 10 repeated flashes; a‐wave, b‐wave, op—oscillatory potential) (Q). Oscillatory potentials (R), mean ERG b‐wave at increasing stimulus flash intensities in P2‐L (S) and P21‐H (T) groups compared with *Ndp*‐KO and WT. Data are shown as individual traces in (Q) and as mean ± SD in (R and T). Data information: Sample numbers for C–N: *n* = 4 per each group. O, P, R analysed with one‐way ANOVA with Sidak's *post hoc* test, S‐T analysed with two‐way repeated measures ANOVA with Tukey's *post hoc* test; all values compared to respective WT (blue asterisks) and *Ndp*‐KO (red asterisks). *Post hoc* test values: *P ≤ 0.05, **P ≤ 0.01, ***P ≤ 0.001, ****P ≤ 0.0001; ns, non‐significant. Sample numbers for ERG analysis: *n* (WT) = 10, *n* (*Ndp*‐KO) = 10, *n* (P2‐L) = 7, *n* (P21‐H) = 10. *n* = biological replicates. GCL, ganglion cell layer; INL, inner nuclear layer; ONL, outer nuclear layer. Numbers 1, 2 and 3 label the superficial, intermediate and deep vascular plexuses. Arrows point to deep plexus vessels. Arrowheads point to abnormal neovascular tufts in *Ndp‐*KO and P21‐H. Scale bars: C–J 50 μm, K–N 20 μm. C–F and G–N images are z‐projections of 20 slices of 0.45 μm. Source data are available online for this figure.

We assessed the effect of AAV9.NDP gene delivery by analysing retinal vasculature (Fig 4A and B) in the eyes of mice at 2 and 3 months of age after treatment at P2 and P21‐H.

**Figure EV2:**
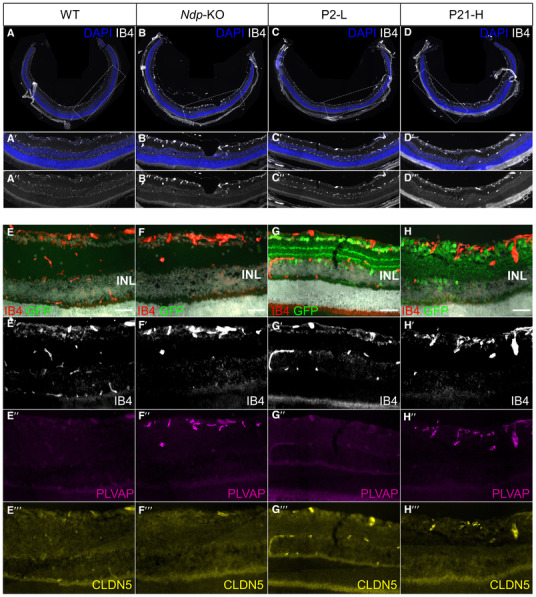
Effects of AAV9.NDP treatment on the retinal vessel morphology and barrier proteins A–H
Retinal cryosections at 2 months stained with IB4 (A–D, E–G, E–G′), anti‐PLVAP and anti‐CLDN5 antibodies (E″–G″) and anti‐EGFP antibody (E–H). Note the presence of three layers of vessels on WT (A‐A″, E‐E′) and P2‐L (C‐C″, G‐G′) groups and only one layer in *Ndp*‐KO (B‐B″, F‐F′) and P21‐H (D‐D″, H‐H′) groups. CLDN5 expression is visible in vessels in WT (E″′), P2‐L (G″′) and P21‐H (H″′) groups. Scale bar 50 μm (E–H). Source data are available online for this figure.

Vascular network formation in each plexus was confirmed in colour‐coded Z‐stack depth projections of retinal whole mounts from each group (Fig 4C–F). WT retinal wholemounts show three vascular plexi (Fig 4C) compared with the presence of only the superficial plexus in the *Ndp*‐KO (Fig 4D). Treatment at P2, but not at P21, rescued all three plexi (Fig 4E and F). Three vascular networks at different depths were also detected in cryosections of WT and P2‐treated *Ndp*‐KO mice (Figs 4G and I, and EV2A‐A″ and C‐C″), but in untreated *Ndp*‐KO and P21‐H treated *Ndp*‐KO mice, only an abnormal superficial vascular plexus was present and individual non‐branching neovascular tufts (Figs 4H and J arrows, and EV2B‐B″ and D‐D″). The blood–retinal barrier is usually established by P17‐P20 (Fruttiger, 2002). At 2 months, immunostaining of the *Ndp*‐KO retina showed reduced expression of claudin‐5 (a structural component of endothelial cell tight junctions) on blood vessels and increased PLVAP (Plasmalemma Vesicle Associated Protein; a component of the transendothelial transport pathway), compared to WT (Figs 4K and L, and EV2E‐E″′ and F‐F″′). Both changes have previously been reported as early markers of the abnormal vasculature in Norrie disease (Wang *et al*, 2012). In both the P2‐L and P21‐H treatment groups, the expression of claudin‐5 was restored and PLVAP staining, typical of the *Ndp*‐KO, disappeared (Figs 4M and N, and EV2G‐G″′ and H‐H″′), consistent with rescue of the blood–retinal barrier. qRT‐PCR analysis was used to measure the levels of gene expression of *Cldn5* and *Plvap* in the *Ndp*‐KO retina after AAV9.NDP treatment at 2 months (Fig 4O and P). The *Ndp*‐KO retina shows the loss of *Cldn5* and upregulation of *Plvap* expression compared to WT. The largest rescue of the dysregulated expression was seen in the P2‐L treatment group. The later treatment groups (P21‐H and P30‐H) showed smaller improvements in expression compared to the untreated *Ndp*‐KO, despite the higher levels of retinal transduction achieved after P21 and P30 treatment compared to P2 (Fig 3).

**Figure EV3:**
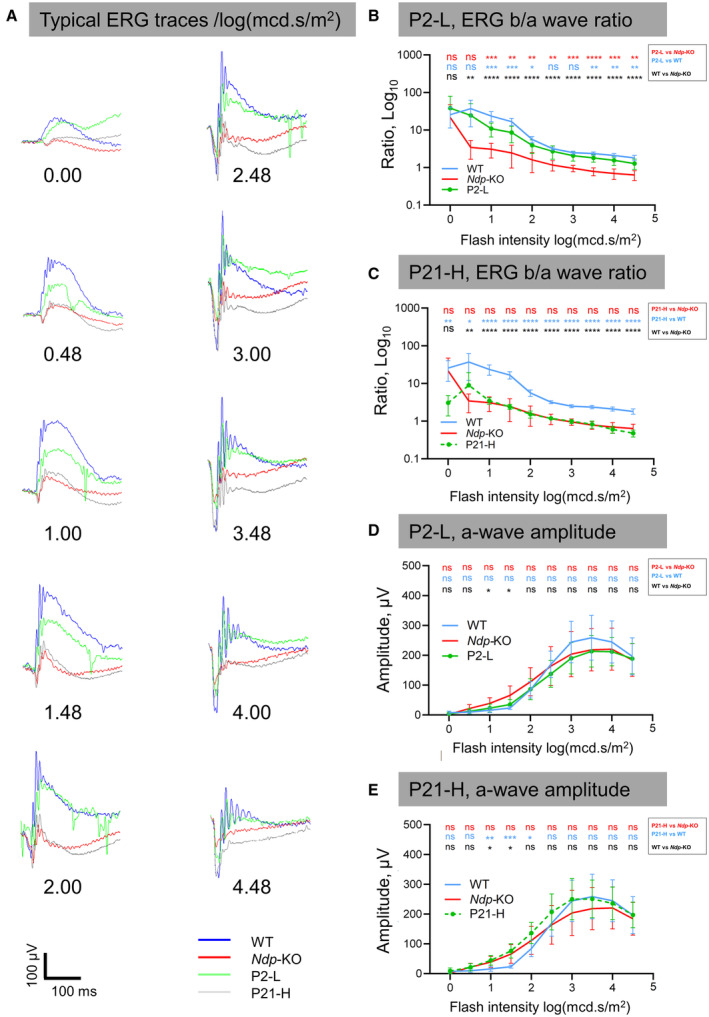
Electroretinograms showing effects of early and late treatments A
Representative ERG traces in response to flashes of light of increasing intensity (an average of 10 flashes shown for each trace).B, C
Flash ERG, ratio of b‐wave to a‐wave amplitude for P2‐L and P21‐H treatment groups.D, E
Flash ERG, a‐wave amplitudes for P2‐L and P21‐H treatment groups. *n* = biological replicates. Data information: *n* (WT) = 10, *n* (*Ndp*‐KO) = 10, *n* (P2‐L) = 7, *n* (P21‐H) = 10. Data are shown as mean ± SD. Statistical analysis was performed by one‐way ANOVA with Sidak's *post hoc* test, comparing each treatment group with WT (blue asterisks) and *Ndp*‐KO (red asterisks), between WT and *Ndp*‐KO (black asterisks). *Post hoc* test values: *P ≤ 0.05, **P ≤ 0.01, ***P ≤ 0.001, ****P ≤ 0.0001: ns, not significant. Source data are available online for this figure.

To assess the effect of AAV9.NDP delivery on visual function, scotopic electroretinograms (ERG) were recorded from the P2‐L and P21‐H treatment groups and controls at 1.5 months of age. Figure 4Q represents the typical scotopic ERG traces of WT, *Ndp*‐KO, P2‐L and P21‐H groups in response to a bright 10 cd × s × m^−2^ flash. The pronounced b‐wave in the WT, signifying signal transduction from photoreceptors to bipolar cells, was almost absent in *Ndp*‐KO. P2‐L‐treated animals resembled the WT and showed partial recovery of the b‐wave and oscillatory potentials, whereas the P21‐H ERG trace was similar to *Ndp*‐KO. Figure EV3 demonstrates the full set of traces for each group (Fig EV3A) and the ratio of b‐wave to a‐wave amplitudes (Fig EV3B and C). An improvement of oscillatory potential amplitudes was observed, though it did not reach significance in either group (Fig 4R).

No differences were found in the a‐wave parameters between WT and *Ndp*‐KO, nor the treatment groups (Fig EV3D and E); the b‐wave amplitudes between WT and *Ndp*‐KO were significantly different with large effect size (Fig 4S and T). In P2‐L, the b‐waves showed significant improvement compared to the *Ndp*‐KO, consistent with the revascularisation of the deep retina (Fig 4S). In P21‐H, the b‐wave amplitude was partially restored (Fig 4T).

In summary, these data indicated the efficacy of intravenous AAV9.NDP vector to ameliorate the retinal pathology in the *Ndp*‐KO mouse. Treatment prior to retinal vascular maturation, but not at later time points, rescued deep retinal vascular pathology. This is consistent with previous studies using genetically engineered mice that showed restoration of the deep vascular plexi in *Ndp*‐KO mice was not possible after maturation (P17‐20) (Wang *et al*, 2012).

### Norrie disease biomarkers in the cochlea respond to AAV9.NDP treatment

**Figure 5:**
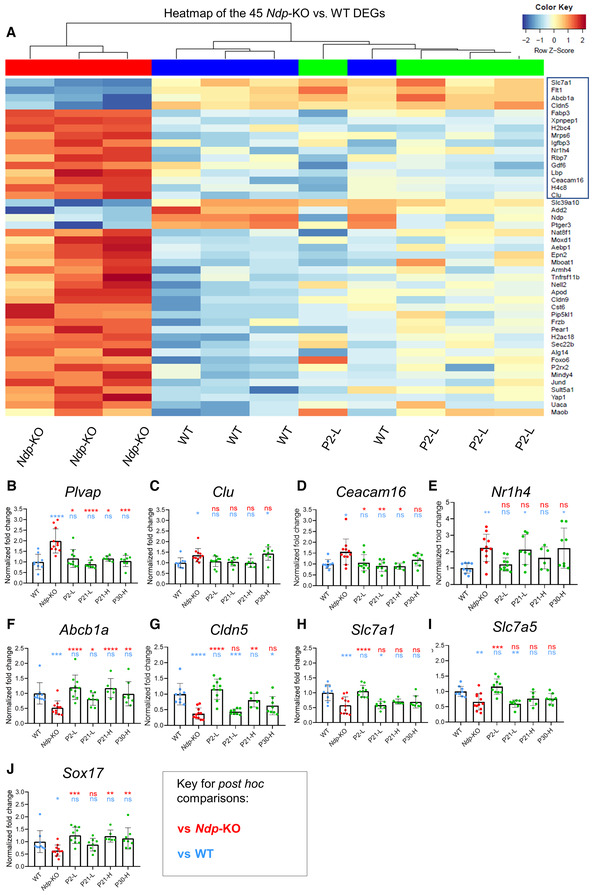
Preservation of gene expression in the cochlea by 2 months of age demonstrated by RNA‐seq and qRT‐PCR A
Heatmap showing levels of expression of 45 pathology‐related DEGs identified between WT (blue) and *Ndp*‐KO (red) in WT, *Ndp*‐KO and P2‐L treated cochleas. Treatment restored expression to levels comparable to the WT (no significant difference in the WT versus P2‐L comparison for all 45 genes). Box indicates 16 genes significantly different in the P2‐L versus *Ndp*‐KO comparison.B–J
qRT‐PCR analysis of the expression of the pathology‐related biomarker genes in all treatment groups. Expression of pathology‐related genes (B) *Plvap*, (C) *Clu*, (D) *Ceacam16*, (E) *Nr1h4*, (F) *Abcb1a*, (G) *Cldn5*, (H) *Slc7a1*, (I) *Slc7a5*, (J) *Sox17*. Data information: Genotype and treatment ages (WT, *Ndp*‐KO, P2‐L, P21‐L, P21‐H, P30‐H) indicated on *x* axes. B–E data are shown as mean ± SD; *n* = biological replicates. Sample numbers: *n* (WT) = 9, *n* (*Ndp*‐KO) = 12, *n* (P2‐L) = 10, *n* (P21‐L) = 8, *n* (P21‐H) = 6, *n* (P30‐H) = 8. Statistical analysis: B–J analysed with one‐way ANOVA with Sidak's *post hoc* test, all values compared to WT (blue asterisks) and *Ndp*‐KO (red asterisks). *Post hoc* test values: *P ≤ 0.05, **P ≤ 0.01, ***P ≤ 0.001, ****P ≤ 0.0001; ns, non‐significant. Source data are available online for this figure.

**Figure EV4:**
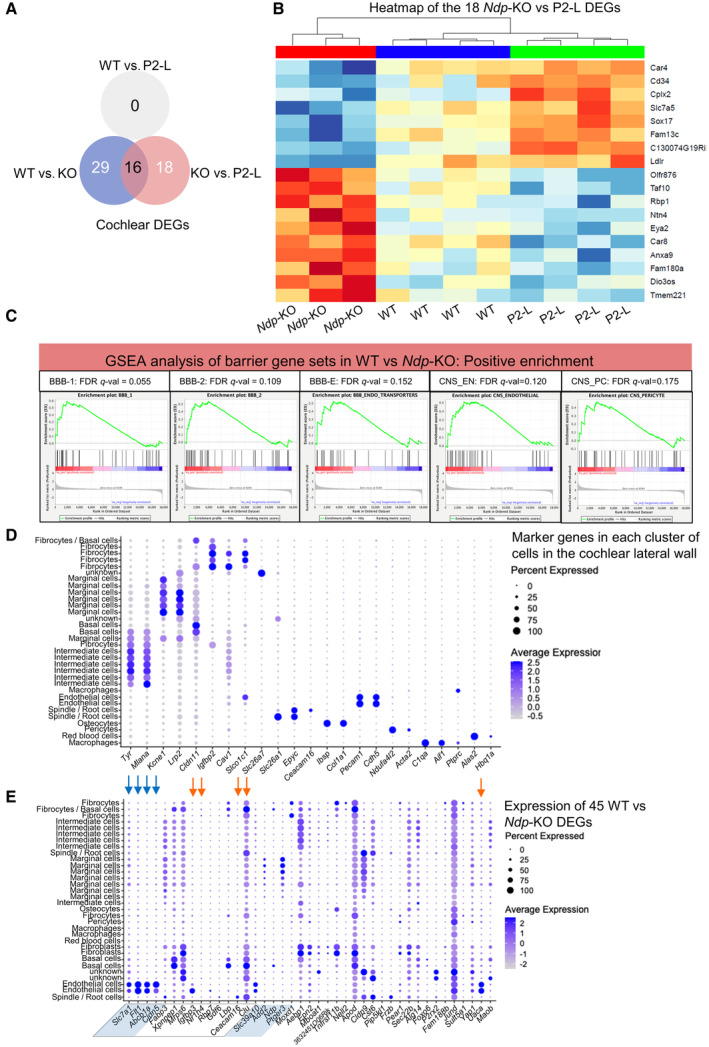
Differential gene expression in the cochlea by RNA sequencing and qRT‐PCR Venn diagram showing overlap between *Ndp*‐KO versus WT DEGs and *Ndp*‐KO versus P2‐L DEGs.Heatmap showing levels of expression of the 18 pathology‐related DEGs identified between *Ndp*‐KO (red) versus P2‐L (blue) in WT, *Ndp*‐KO and P2‐L treated cochleas (green). Note that these genes showed trends of differential expression between *Ndp*‐KO and WT cochleas that did not reach significance. No genes were found to be significantly differentially expressed between WT and P2‐L cochleas, indicating rescue by the treatment. In heat map, red indicates upregulated and blue indicates downregulated gene expression in *Ndp*‐KO.Gene set enrichment analyses of differentially expressed genes between WT and *Ndp*‐KO using gene sets were previously defined by transcriptome profiling of FACS sorted CNS vs peripheral endothelial cells (Daneman *et al*, 2010) and previously used to assess transcriptomes of WT and *Ndp*‐KO retinas (Zhou *et al*, 2014). Gene sets characterising barrier vasculature, BBB1 and 2, BBB endothelial transporters, CNS endothelial and CNS pericyte were significantly positively correlated (FDR < 0.25) with the WT genotype.Dot plot using scRNA seq data of the adult mouse cochlear lateral wall from GEO database: accession numbers GSM5124299, GSM5124300, GSM5124301, and GSM5124302. Gene markers used to distinguish 30 cell type clusters in the UMAP were as previously reported (Gu *et al*, 2020; Bryant *et al*, 2022).Dot plot showing expression of the 45 DEGs identified in WT versus *Ndp*‐KO analysis in the 30 cell type clusters identified in the adult mouse cochlear lateral wall at the single cell level. Blue arrows indicate endothelial cell genes (*Cldn5*, *Abcb1a* and *Flt1*) downregulated in *Ndp*‐KO, and orange arrows indicate upregulated genes in *Ndp*‐KO. Box indicates genes significantly different in the P2‐L versus *Ndp*‐KO comparison. Shaded DEGs are downregulated in the *Ndp*‐KO cochlea. Note expression of some DEGs across several different cell type clusters.

As understanding of the downstream molecular mechanisms that lead to cochlear insults in Norrie disease is limited, we next aimed to define new biomarkers of the cochlear disease in order to assess treatment efficacy. We compared patterns of gene expression in the cochlea of WT and *Ndp*‐KO and in *Ndp*‐KO mice P2 treated mice. Dysregulated gene expression profiles were identified by RNAseq analysis of the whole cochlea from WT (*n* = 4), *Ndp‐*KO (*n* = 3) and P2‐L (*n* = 4) mice at 2 months. Forty‐five significantly differentially expressed genes (DEGs, adjusted *P* < 0.05) were identified between WT and *Ndp*‐KO cochlea (Fig 5A; Appendix Dataset EV3). There were no DEGs between the WT and treated *Ndp*‐KO P2‐L groups indicating rescue resulting from treatment. Unsupervised clustering also showed the treated *Ndp*‐KO P2‐L samples clustering with the WT samples rather than the untreated *Ndp*‐KO samples (Fig 5A). Thirty‐four DEGs were also identified between *Ndp*‐KO and P2‐L samples (Dataset EV3); 16 overlapped with the set of 45 disease biomarker DEGs (Figs 5A boxed, and EV4A) and 18 showed enhanced responses to treatment (Fig EV4B; Dataset EV3).

Gene set enrichment analysis (GSEA) showed enrichment of endothelial barrier gene sets in the *Ndp*‐KO (Fig EV4C). Endothelial cell DEGs associated with the normal function of cochlear microvasculature were identified as likely downstream targets of NDP signalling. Barrier gene *Cldn5*, vascular endothelial growth factor receptor 1 gene (*Flt1*), which is important for vascular barrier and branching (Eilken *et al*, 2017; Wang *et al*, 2019; Zhang *et al*, 2021), and molecule transporter genes, *Abcb1a*, *Slc7a1*, were all downregulated in the *Ndp*‐KO and returned to normal levels with treatment (Fig 5A). *Abcb1a* is associated with hearing loss and increased sensitivity to ototoxicity in mice (Zhang *et al*, 2000; Saito *et al*, 2001). *Slc7a1* is an amino acid transporter, typical to normal blood–brain barrier (Yahyaoui & Pérez‐Frías, 2019). *Slc7a5*, another amino acid transporter gene known to be expressed in cochlear vasculature (Sharlin *et al*, 2011), showed increased expression after treatment (Fig EV4B). Investigation of a scRNAseq atlas data set of the mouse cochlea confirmed that these genes are expressed in vascular endothelial cells of the cochlear lateral wall (Fig EV4D and E). These findings of down regulation of endothelial cell barrier markers and transporters in the *Ndp*‐KO were consistent with microvasculature as a primary site of pathology in Norrie disease, supporting the hypothesis that microvascular disruption leads to an unsuitable microenvironment for hair cell survival in the Norrie cochlea. The genes upregulated in the *Ndp*‐KO are also expressed in the lateral wall (Fig EV4D and E) and considering their function could be related to the Norrie disease cochlear pathology (Figs 5A and EV4). *Clu* is expressed in multiple cell types in the cochlea and encodes a secreted chaperone protein (Lee *et al*, 2017) known to be involved in responses to cell and tissue damage (Rohne *et al*, 2016). Ceacam16 is a secreted glycoprotein that interacts with the acellular tectorial membrane and is critical for maintaining this structure (Zheng *et al*, 2011). It is also expressed in spindle/root cells of the lateral wall (Fig EV4E; Gu *et al*, 2020). Nr1h4 is thought to play a role in vascular endothelial homeostasis (He *et al*, 2006). Of note is the fact that several of the cochlea DEGs have also been identified in studies of differential gene expression in the *Ndp*‐KO retina. For example, *Cldn5* and *Slc7a1* were identified as downregulated in the postnatal *Ndp*‐KO retina (Schafer *et al*, 2009; Zhou *et al*, 2014), suggesting that NDP signalling acts on similar pathways needed for vascular endothelial cell function in the cochlea and the retina.

The expression of the identified biomarkers of cochlear pathology was analysed by qRT‐PCR analysis of whole cochlea samples at 2 months after AAV9.NDP treatment at early (P2‐L) or at the later time points (P21‐L, P21‐H, P30‐H groups) to compare treatment efficacy. We also performed comparative analysis of the levels of *EGFP‐P2A‐NDP* transgene expression, the transcellular permeability gene *Plvap*, which we previously showed was dysregulated in the *Ndp*‐KO cochlea at 2 months (Bryant *et al*, 2022) and *Sox17*, a transcription factor gene known to be upregulated in retinal endothelial cells in response to Ndp signalling (Ye *et al*, 2009).

qRT‐PCR confirmed significant differential expression between *Ndp*‐KO and WT for nine genes; *Plvap*, *Clu*, *Ceacam16*, *Nr1h4* were upregulated in the *Ndp*‐KO; *Abcb1a*, *Cldn5*, *Slc7a1*, *Slc7a5* and *Sox17* were downregulated (Fig 5B–J, two‐way ANOVA with Tukey's *post hoc* test *P* < 0.05). At 2 months, disease biomarker gene expression returned to WT expression levels in the neonatal P2‐L and juvenile P21‐H treatment groups (all nine genes), and in the young adult P30‐H group (all except *Cldn5*, *Clu*, *Nr1h4*), while the low‐dose P21‐L treatment was less effective (Fig 5B–J) (blue, ns, indicating gene expression showing no significant difference from WT in each treatment group).

These data indicate that dysregulated gene expression levels found in the *Ndp*‐KO was restored to that of the WT, not only after neonatal treatment but also after later treatment of juvenile and young adult mice at later stages of pathology. Overall P2 injection better recapitulated the WT expression level of cochlear genes (Fig 5E–I) (see red asterisks indicating significant difference from *Ndp*‐KO, as well as no significant difference from WT, blue, ns). These patterns of rescue of gene dysregulation are in line with the levels of GFP transduction and transgene expression in the cochlea (Fig 3) whereby the highest levels of cochlea transduction were shown after treatment at P2. Since several of these genes are biomarkers for cochlear microvascular pathology, our results suggest that delivery of NDP by gene therapy may maintain and restore cochlear barrier and transport function.

### Effect of AAV9.NDP treatment on the lateral wall vasculature

To assess whether the restored biomarker gene expression patterns correspond with rescue of tissue pathology, cochlear whole mounts from AAV9.NDP treated mice were analysed by immunostaining to assess the effect on lateral wall microvasculature morphology and survival of hair cells in the organ of Corti.

**Figure 6:**
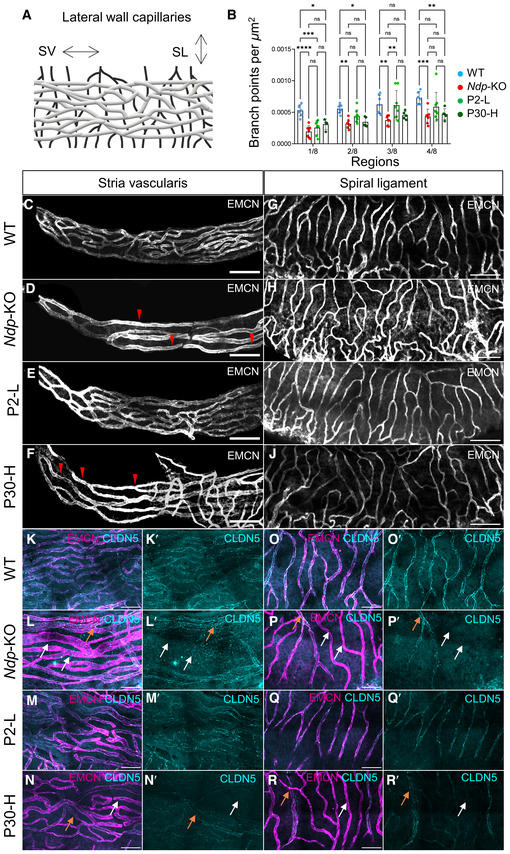
Differences of early and late treatment efficacy for the rescue of the cochlear vasculature at 2 months A
Schematic of the lateral wall vasculature. SL, spiral ligament; SV, stria vascularis capillaries.B
Quantification of capillary branching point numbers per area in sequential apical regions 1–4 along the stria vascularis. Branchpoint number was significantly reduced in *Ndp*‐KO compared to WT. Note that branching was improved more in the P2‐L than in the P30‐H treatment groups. No significant differences in capillary branch point number between WT and *Ndp*‐KO were detected in the rest of the cochlea, regions 5–8. *n* = biological replicates. WT, *n* = 6; *Ndp*‐KO, *n* = 6; P2‐L *n* = 8; P30‐H, *n* = 5.C–F
Capillary network density and morphology at the apical tip of the stria vascularis labelled by anti‐endomucin staining (EMCM). Scale bar: 100 μm, *n* = 4 per each group. (C) WT, *n* = 4; (D) *Ndp*‐KO, *n* = 4; (E) P2‐L, *n* = 5; (F) P30‐H, *n* = 4. Red arrowheads indicate reduced network density and enlargement of vessel diameter.G–J
Capillary network density by anti‐endomucin staining (EMCM) in the spiral ligament showing more irregular branching vasculature in *Ndp*‐KO and P30‐H compared to WT and P2‐L. Scale bar: 100 μm, *n* = 4 per each group. (G) WT, (H) *Ndp*‐KO, (I) P2‐L, (J) P30‐H.K–R
Immunostaining for tight junction marker claudin‐5 (CLDN5) in anti‐endomucin stained capillaries of the stria vascularis (K–N) and the spiral ligament (O–R). Claudin‐5 shows atypical uneven expression in *Ndp*‐KO and P30‐H compared to regular labelling of vessels in WT and P2‐L samples. White arrows indicate atypical vessels labelled with endomucin but with low/absent claudin‐5, and orange arrows indicate endomucin‐stained vessels with high claudin‐5 expression. Scale bar 50 μm. Data information: B data are shown as mean ± SD. Statistical analysis: two‐way repeated measures ANOVA with Tukey's *post hoc* test, all values compared to WT (blue) and *Ndp*‐KO (red). *Post hoc* test values: *P ≤ 0.05, **P ≤ 0.01, ***P ≤ 0.001, ****P ≤ 0.0001; ns, non‐significant. Source data are available online for this figure.

Previously, we identified malformation of cochlear microvasculature to be an early finding in disease development (Bryant *et al*, 2022). Endomucin immunostaining was used to compare the lateral wall vasculature morphology in the stria vascularis and the spiral ligament (Fig 6A–J) after treatment. Quantification of branching points in stria vascularis vessels along the lateral wall demonstrated significantly reduced branching in the *Ndp*‐KO compared to the WT only in the strial apical region (regions 1/8 to 4/8 along the apex‐to‐base axis), which was better rescued in the P2‐L than in the P30‐H treatment group (Fig 6B–F). Appendix Fig S6A–E shows representative images of a dissected cochlea and mapping of the lateral wall into eight equal regions. In the spiral ligament, the vessels of the P2‐L treatment group, but not the P30‐H group, resembled the WT (Fig 6G–J). In the P30‐H treatment groups, the vasculature networks showed an atypical contorted appearance similar to that of the *Ndp*‐KO (Fig 6H and J).

Immunostaining for claudin‐5 (a component of endothelial cell tight junctions previously reported as a marker of the abnormal vasculature in Norrie disease; Bryant *et al*, 2022) showed low/absent claudin‐5 in most of the *Ndp*‐KO blood vessels of the stria vascularis and spiral ligament compared to WT (Fig 6K–P, white arrows). A few atypical vessels showed high claudin‐5 (orange arrows). In the P2‐L, but not the P30‐H, treatment groups, claudin‐5 was restored (Fig 6M–R) and was comparable with the WT distribution.

In the spiral ligament capillary and stria vascularis microvasculature networks, treatment of neonates, but not older mice, ameliorated the pathology. These data suggest that NDP is required for the early development of lateral wall vasculature which is still forming at P2, and this pathology is irreversible with treatment at later timepoints once maturation is complete (P20) (Ando & Takeuchi, 1998).

### AAV9.NDP prevents sensory hair cell loss even after the onset of degeneration

**Figure 7:**
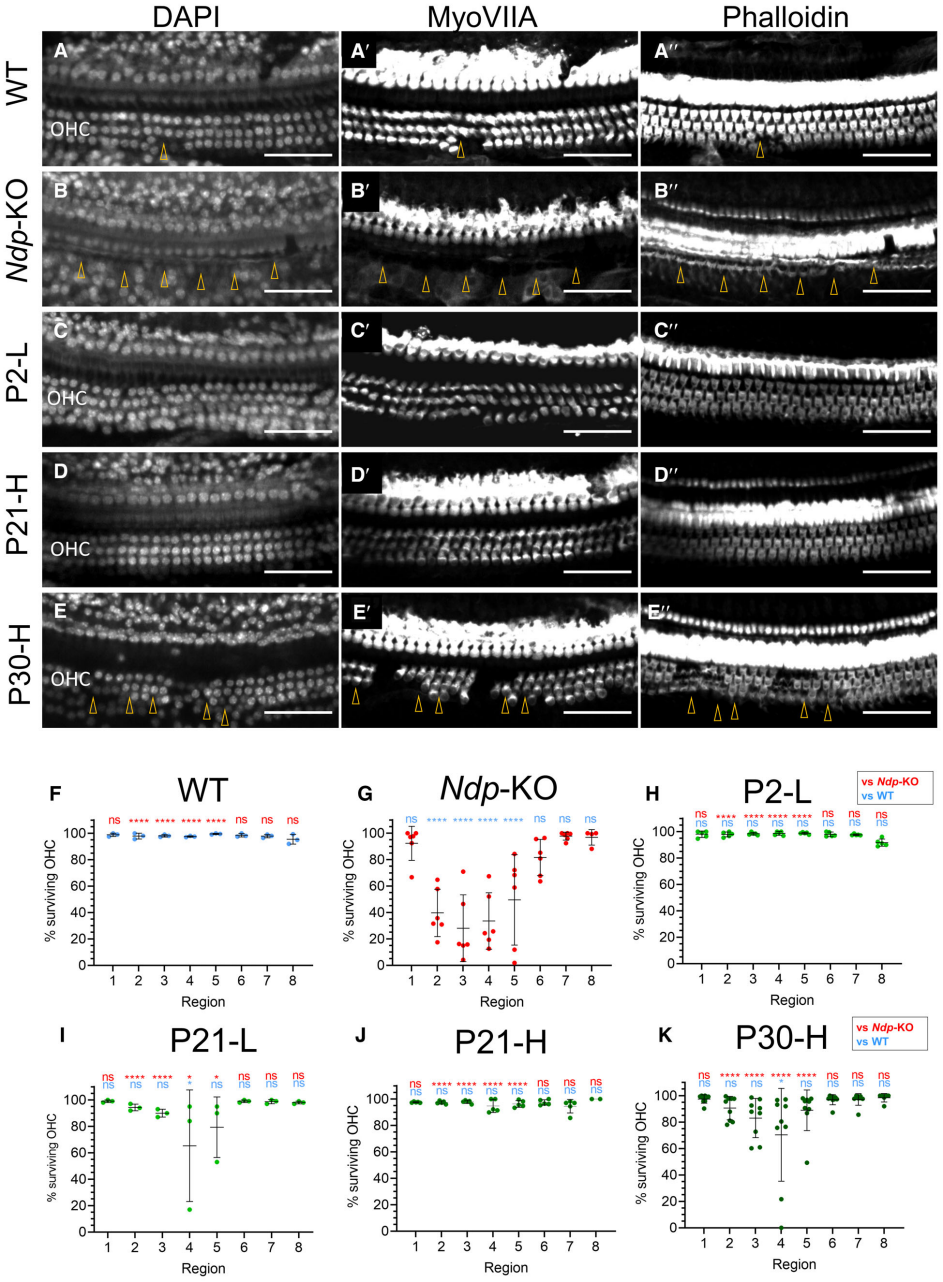
Preservation of the outer hair cells in all treatment groups by 2 months A–E
Examples of hair cell survival in matching “sensitive” region 2/8 corresponding to 6.1–10 kHz along the tonotopic axis from different treatment groups. A–E: DAPI, A′‐E′: MyoVIIA immunostaining, A″‐E″: phalloidin. (A) WT, *n* = 7; (B) *Ndp*‐KO, *n* = 6; (C) P2‐L, *n* = 5; (D) P21‐H, *n* = 6; (E) P30‐H, *n* = 8. Arrowheads indicate site of hair cell loss, OHC, outer hair cell. Scale bar 50 μm.F–K
Quantification of the surviving hair cells from the same samples groups as in A–E. Regions of the organ of Corti defined as fractional distance from the apex (1/8 to 8/8): region 1 (3.1–6.1 kHz), region 2 (6.1–10.0 kHz), region 3 (10.0–15.0 kHz), region 4 (15.0–21.6 kHz), region 5 (21.6–30.2 kHz), region 6 (30.2–41.3 kHz), region 7 (41.3–55.9 kHz), region 8 (55.9–74.8 kHz). Analysed with two‐way repeated measures ANOVA with Tukey's *post hoc* test, samples compared to the WT (blue asterisks) and *Ndp*‐KO (red asterisks). *n* = biological replicates. (F) WT, *n* = 3; (G) *Ndp‐*KO, *n* = 6; (H) P2‐L, *n* = 5; (I) P21‐L, *n* = 3; (J) P21‐H, *n* = 6; (K) P30‐H, *n* = 8. Data information: Quantification data are shown as mean ± SD. Significant effects of region (*P* < 0.0001), treatment group (*P* < 0.0001) and their interaction (*P* < 0.0001). *Post hoc* test values: *P ≤ 0.05, **P ≤ 0.01, ***P ≤ 0.001, ****P ≤ 0.0001; ns, non‐significant. Source data are available online for this figure.

At 2 months, hair cells in the WT cochlea were intact (Fig 7A‐A″), while *Ndp‐*KO had a severe degeneration of OHCs in the mid frequency region (images of apical region corresponding to 6–10 kHz; Fig 7B‐B″). This was consistent with our previous study (Bryant *et al*, 2022). In the treatment groups, OHCs were either preserved entirely, as in groups P2‐L and P21‐H (Fig 7C‐C″ and D‐D″), or partially as in group P30‐H (Fig 7E‐E″). The surviving OHCs were quantified (Fig 7F–K) in whole mounts of the organ of Corti, mapped into regions of equal distance along the apex‐to‐base axis. Data were analysed with two‐way repeated measures ANOVA with Tukey's *post hoc* test for each treatment group individually, compared with the corresponding regions of the WT and *Ndp‐*KO (Fig 7F–K). Analysis confirmed severe degeneration of OHCs in the mid frequency region in regions 2–5 out of 8 corresponding to 6–30 kHz in the *Ndp*‐KO and a complete OHC rescue in P2‐L and P21‐H (Fig 7H and J) groups and significant improvement in the “sensitive” region (2/8–5/8 from the apex) of P21‐L and P30‐H samples (Fig 7I and K). Appendix Fig S6F–K shows region mapping and hair cell survival along the apex‐base axis of the organ of Corti in all groups.

The cochlear whole mount analyses indicated that the treatment of neonates (P2‐L) and juveniles improved microvasculature and prevented onset of hair cell degeneration. Importantly, later treatment of young adults (P30‐H) also reduced hair cell degeneration.

### AAV9.NDP treatment of *Ndp*‐KO mice rescues the auditory function decline

**Figure 8:**
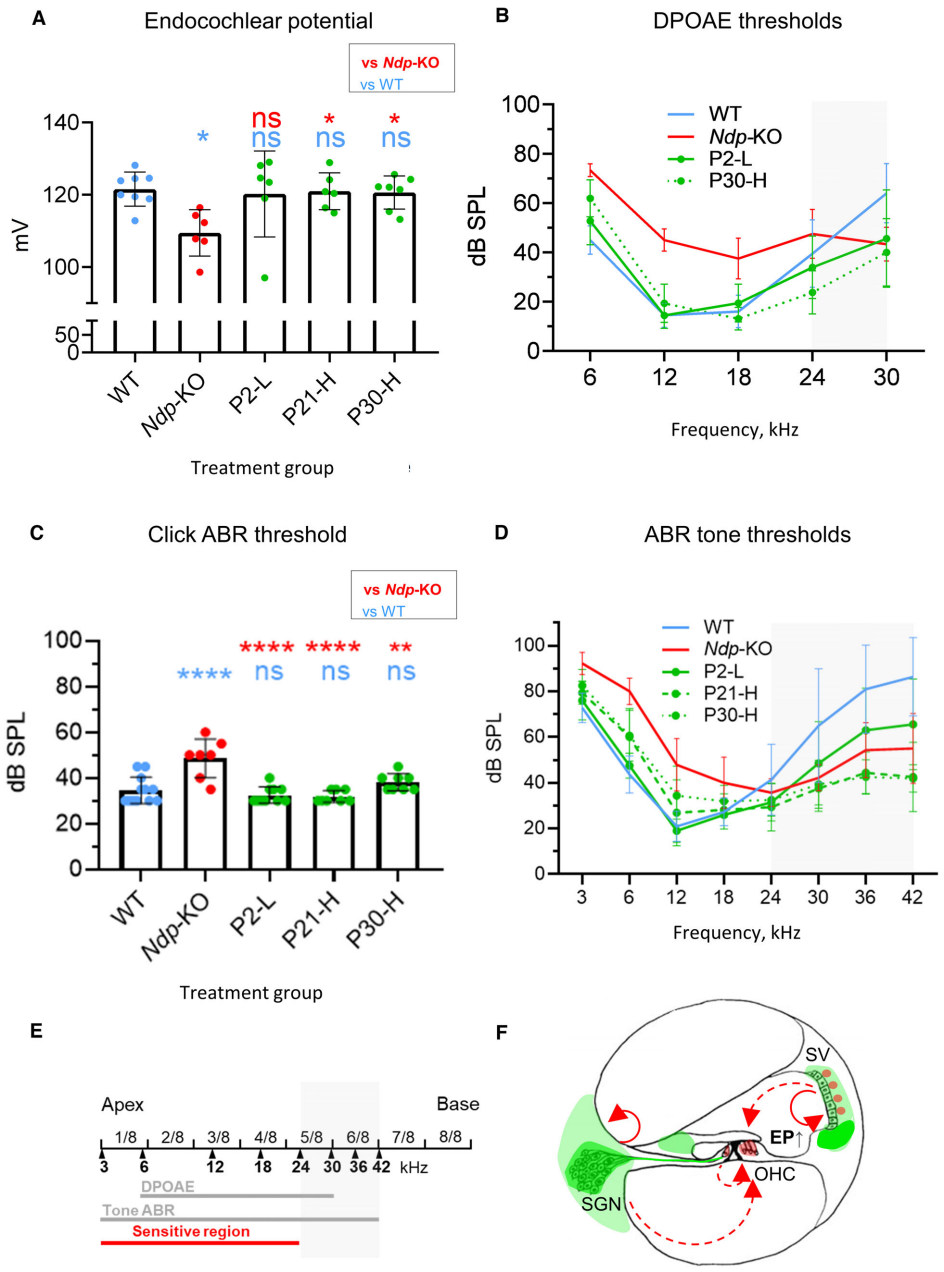
Auditory function at 3 months Endocochlear potentials (mean ± SD) analysed with one‐way ANOVA with Sidak's *post hoc* test, each treatment group compared to the WT (blue asterisks) and *Ndp*‐KO (red asterisks). *P (*Ndp*‐KO vs. WT) = 0.0107.Overlay of DPOAE thresholds (mean ± SD). Grey area marks high‐frequency region affected in the WT. Statistical analysis for (B) is provided in Fig EV7.Click ABR thresholds (mean ± SD) analysed with one‐way ANOVA with Sidak's *post hoc* test, each group compared to WT (blue asterisks) and *Ndp*‐KO (red asterisks).Overlay of pure tone ABR thresholds from control and treatment groups (mean ± SD). Grey area marks region affected in the WT. Statistical analysis for (D) is provided in Fig EV7.Schematic of tonotopic region correspondence with auditory function measures. The whole length of the cochlea is divided in eight equal length regions (1/8–8/8). Black arrowheads mark the respective frequencies (kHz), to which specific points correspond. Red line indicates the tonotopic region, sensitive to degeneration in the *Ndp*‐KO. Grey lines indicate frequency regions, in which the ABR and DPOAE were recorded. Grey area marks region affected in the WT by age‐related degeneration.Schematic of the putative Norrie phenotype rescue mechanism by gene therapy. Green colour labels the areas typically transduced in all treatment groups, and red arrows indicate the putative targeting sites of the NDP, produced in and secreted from the transduced areas (green). SV, stria vascularis; SGN, spiral ganglion; OHC, outer hair cell; EP, endocochlear potential. Data information: Data are shown as mean ± SD. Animal numbers for auditory function analyses: *n* = biological replicates. Endocochlear potential: *n* (WT) = 8, *n* (*Ndp*‐KO) = 6, *n* (P2‐L) = 6, *n* (P21‐H) = 6, *n* (P30‐H) = 7. DPOAE: *n* (WT) = 11, *n* (*Ndp*‐KO) = 6, *n* (P2‐L) = 9, *n* (P30‐H) = 8. Click and pure tone ABR: *n* (WT) = 12, *n* (*Ndp*‐KO) = 7, *n* (P2‐L) = 10, *n* (P21‐H) = 8, *n* (P30‐H) = 8. *Post hoc* test values: *P ≤ 0.05, **P ≤ 0.01, ***P ≤ 0.001, ****P ≤ 0.0001; ns, non‐significant. Source data are available online for this figure.

Finally, to evaluate the therapeutic effect on hearing, we performed electrophysiological assessment of cochlear function at 3 months of age (Fig 8). Endocochlear potential (EP; the high resting potential in the endolymph essential for sound transduction) was significantly reduced in *Ndp‐*KO compared to WT, but recovered in the mice treated as neonates (P2‐L), juvenile (P21‐H) or young adult (P30‐H) mice (Fig 8A).

**Figure EV5:**
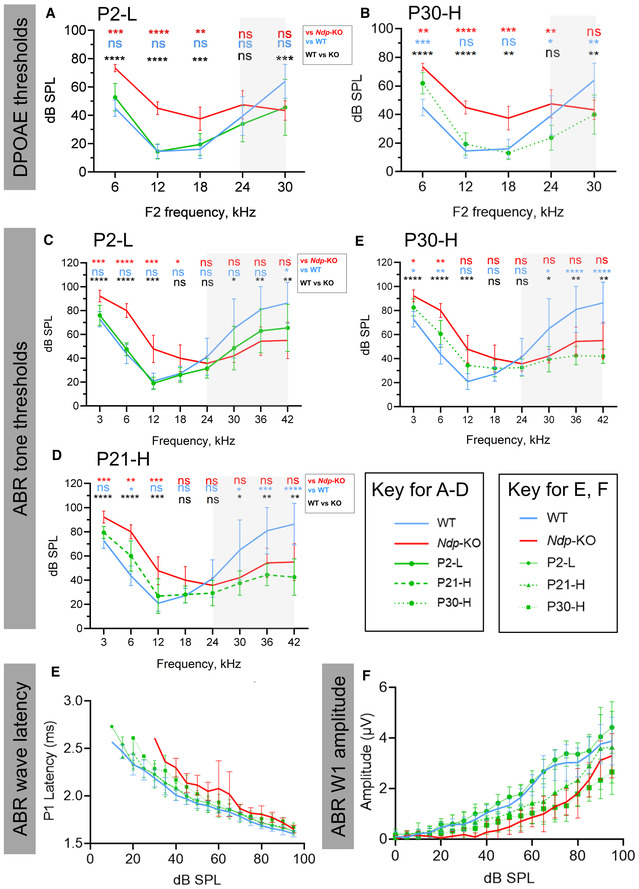
Statistical analysis of auditory function analysis of DPOAE and ABR thresholds for all groups at 3 months A, B
DPOAE thresholds of P2‐L, P30‐H and control groups. *P* values indicate statistically significant difference from WT (blue) and *Ndp*‐KO (red). *n* = biological replicates. *n* (WT) = 11, *n* (*Ndp*‐KO) = 6, *n* (P2‐L) = 9, *n* (P30‐H) = 8.C–E
ABR thresholds of P2‐L, P‐30H and control groups. *P* values (blue) indicate statistically significant difference from WT. *n* = biological replicates. *n* (WT) = 12, *n* (*Ndp*‐KO) = 7, *n* (P2‐L) = 10, *n* (P21‐H) = 8, *n* (P30‐H) = 8.E
ABR wave latency for each treatment group compared to *Ndp*‐KO and WT. *n* = biological replicates. *n* (WT) = 12, *n* (*Ndp*‐KO) = 7, *n* (P2‐L) = 10, *n* (P21‐H) = 8, *n* (P30‐H) = 8.F
ABR wave 1 amplitude for each treatment group compared to *Ndp*‐KO and WT. *n* = biological replicates. *n* (WT) = 12, *n* (*Ndp*‐KO) = 7, *n* (P2‐L) = 10, *n* (P21‐H) = 8, *n* (P30‐H) = 8. Data information: Data are shown as mean ± SD. Statistical analysis was performed by two‐way repeated measures ANOVA with Tukey's *post hoc* test, comparing each treatment group with WT (blue asterisks) and *Ndp*‐KO (red asterisks), and comparing between WT and *Ndp*‐KO (black asterisks). *Post hoc* test values: *P ≤ 0.05, **P ≤ 0.01, ***P ≤ 0.001, ****P ≤ 0.0001. Source data are available online for this figure.

To estimate the functionality of outer hair cells, we measured distortion product otoacoustic emissions (DPOAEs) to f2 frequencies of 6–30 kHz. An overlay of 2f1‐f2 DPOAE average thresholds is shown in Fig 8B, and statistical analysis in Fig EV5A and B. Thresholds in the mid frequency regions at 6–18 kHz were significantly elevated in the *Ndp*‐KO compared to the WT (***P ≤ 0.001, black asterisk), consistent with loss of hair cell integrity at 2 months in these regions of the organ of Corti (see Fig 7); and our previous analysis (Bryant *et al*, 2022). These were fully rescued after treatment of neonates (P2‐L) (Figs 8B and EV5A; **P ≤ 0.01, P2‐L versus *Ndp*‐KO red asterisks; not significantly different from WT, ns, blue). Treated young adults also showed DPOAE thresholds indistinguishable from that of the WT at 12–18 kHz (Figs 8B and EV5B; **P ≤ 0.01, P30‐H versus *Ndp*‐KO red asterisks; not significantly different from WT, ns, blue) consistent with the survival of functional outer hair cells observed in the organ of Corti after treatment.

Unexpectedly, at the highest F2 frequency tested (30 kHz), WT mice showed significantly elevated thresholds compared to the AAV9.NDP treated mice and to the untreated *Ndp*‐KO mice (Figs 8B, shaded grey and EV5). This may reflect onset of age‐related hearing loss in the WT mice, which is a documented feature of C57BL/6 mice (Johnson *et al*, 1997), but does not explain why thresholds are lower in the *Ndp*‐KO litter mates. Difference in the presence of the *ahl* genotype was excluded as genotyping confirmed the expected homozygosity for the *Cdh23* ^*753A*^ age‐related hearing loss (ahl1) allele in all experimental mice. We have provided the complete data set including electrophysiological analyses at all frequencies, greying the high‐frequency range, for data clarity (Fig 8).

To further evaluate the prevention of hearing loss after AAV9.NDP treatment, we recorded auditory brainstem responses (ABR) to broadband (click) (Fig 8C) and pure tone stimulus (Fig 8D). The click thresholds were significantly increased in *Ndp*‐KO in comparison to WT but returned to WT values in all treatment groups (Fig 8C), indicating that a good overall rescue of hearing was achieved.

Analysis of the frequency‐specific ABRs to pure tone stimuli at 3–42 kHz showed that the *Ndp*‐KO thresholds were significantly elevated at 3–12 kHz frequencies compared to WT (average thresholds shown in Fig 8D, and statistical analysis in Fig EV5C–E; ***P ≤ 0.001, black asterisks). After AAV9.NDP treatment of neonates (P2‐L), juvenile (P21‐H) and young adult (P30‐H) mice, thresholds at the 3–12 kHz frequencies were lowered in all groups compared to *Ndp*‐KO (Fig EV5D; **P ≤ 0.01, P21‐H versus *Ndp*‐KO, red asterisks; Fig EV5E; **P ≤ 0.05 P30‐H versus *Ndp*‐KO at 3 and 6 kHz, red asterisks). Complete restoration to WT thresholds was achieved only with neonatal treatment (Figs 8D and EV5C; ***P ≤ 0.001, P2‐L versus *Ndp*‐KO red asterisks; not significantly different from WT at 3–12 kHz, ns, blue). In line with the DPOAE analysis, thresholds of the WT were increased ≥ 30 kHz, while untreated *Ndp*‐KO and the treated groups (P2‐L, P21‐H, P30‐H) maintained hearing within normal limits (Figs 8D and EV5C and D).

Analyses of the latency of the first positive ABR wave (P1) and the peak‐to‐peak wave 1 amplitude (Fig EV5E and F) for responses evoked by 12 kHz tone stimuli showed that latency was prolonged and amplitude was reduced in the *Ndp*‐KO mice, compared to untreated wild type. Following treatment at P2, these measures returned to values comparable to untreated wild types.

In summary, these EP, DPOAE and ABR results indicate that hearing function can be preserved by AAV9.NDP gene therapy after treatment at a range of disease stages. Considered together, these data suggest that rescue is mediated via prevention of progressive hair cell loss and preservation of the OHC function, as the affected and rescued tonotopic regions coincided in the DPOAE and tone ABR thresholds (Fig 8E). Figure 8F shows a schematic model of Norrie phenotype rescue by gene therapy.

## Discussion

### AAV9.NDP treatment prevents disease progression

This proof‐of‐concept study has investigated the applicability of gene therapy to the treatment of Norrie disease. We have for the first time demonstrated that early treatment effects complete rescue of cochlear pathology and retinal vasculature, proving that our vector is delivering functional human norrin. The early treatment time point corresponds to the foetal stage in human development. Foetal gene therapy has, as yet, only been performed in animal models. *NDP* gene delivery may exert adverse effects on placental vasculature (Luhmann *et al*, 2005b) or the developing foetus (Ye *et al*, 2009). However, we also show that the Norrie cochlea is responsive to treatment in later stages of the disease, preventing hearing loss after the onset of degenerative changes. These changes correspond to childhood or young adulthood in Norrie patients, after cochlear development is complete. This suggests that the treatment of patients may be both feasible and deliverable. Since cell turnover in the cochlea is low, AAV9.NDP gene replacement therapy could enable long‐term production of norrin to maintain cochlear vessel barrier function throughout life. Currently without treatment, hearing loss starts around adolescence and severely disrupts the life quality of already blind patients.

In the eye, lack of norrin results in failure to develop the two deep layers of retinal vasculature resulting in tractional retinal detachment from birth. Vision loss in Norrie disease is, therefore, usually present from birth (Redmond *et al*, 1993). Our work has demonstrated that although the missing deep retinal vasculature cannot be regrown with NDP after vascular network maturation, consistent with previous work (Wang *et al*, 2012), the retinal vascular barrier does respond to postnatal AAV9.NDP and remains responsive to treatment even after vascular development is complete. This offers a potential route to treat milder NDP‐associated ocular conditions such as Familial Exudative Vitreoretinopathy (FEVR) and Coats disease in patients where the predominant cause of vision loss is vascular exudation as opposed to tractional retinal detachment.

### The role of vasculature in the Norrie cochlear phenotype

The responsiveness of the cochlea to treatment at late timepoints appears consistent with the pathology being mediated by vascular barrier dysfunction. Lack of NDP in the mouse cochlea causes morphological vessel abnormalities and disrupts the cochlear vascular barrier (Rehm *et al*, 2002; Bryant *et al*, 2022).

Integrity of the cochlear vascular barrier is essential for maintenance of the endocochlear potential, and hence for the survival and function of the hair cells (Liu *et al*, 2016). RNAseq and qRT‐PCR analysis of whole cochlea lysates demonstrated that there was a pronounced dysregulation of vascular barrier and transporter genes in the *Ndp‐*KO, but preservation of expression levels after AAV9.NDP treatment at early and later timepoints. The RNAseq study was informative as it showed the rescue effect at the molecular level by restoring gene expression deficient in the *Ndp*‐KO model. The confirmation by qRT‐PCR correlated well with the rescue, i.e. P2‐L injection restored downstream gene expression robustly, which was associated with better functional outcomes. Notably, restoration of endothelial gene expression to WT levels was also achieved after treatment at later timepoints (e.g. *Plvap*, *Abcb1a* and *Sox17*). Restoration of cochlea gene expression by treatment at later timepoints, even without restoring the cochlear vascular morphology was associated with the preservation of endocochlear potential, OHCs and hearing. The reversibility of the blood vessel barrier within the cochlear vasculature even as disease progresses therefore allows restoration of the normal hair cell environment from the timepoint of treatment and hence their survival and normal function.

It has been suggested that in the cochlea, norrin may act directly on the hair cells via the transcription factor Pou4f3 and is needed for regulating hair cell maturation (Hayashi *et al*, 2021). Constitutive *Ndp* overexpression in supporting cells or neonatal β‐catenin stabilisation in hair cells using *Atoh1‐*Cre was reported to preserve OHC survival (Hayashi *et al*, 2021). In our study, we did not detect a reduction in hair cell marker gene (*Myo7a* and *Pou4f3*) expression by RNAseq analysis at 2 months of age (Dataset EV1), even though marked hair cell loss is apparent by 2 months (Bryant *et al*, 2022). This may be due to the low resolution of bulk RNAseq analysis and/or the sensitivity achievable in analysis of whole cochlea. However, our previous study specifically shows that OHCs develop and mature and are functional in the *Ndp*‐KO by 1 month (Bryant *et al*, 2022), and we now show that they are able to survive (Fig  7) and function (Fig  8, DPOAEs) long term if NDP is restored at P21 or 1 month of age.

### Developing clinical AAV9.NDP gene therapy and safety

Gene therapy is a quickly moving field. Although a majority of treatments are at the pre‐clinical trial stage, four AAV‐mediated therapies—Luxturna^®^, Zolgensma^®^, Upstaza^®^ and Roctavian^®^—have been approved to treat severe genetic disorders. Secreted signalling protein gene therapy is not yet widely studied, and there are potential side‐effects of unregulated prolonged expression. The viral dose used to achieve rescue of the cochlea in this study was low, approximately 5–25 times lower than that of clinically approved Zolgensma^®^ for the treatment of the life‐limiting condition spinal muscular atrophy which used a similar AAV9 vector by intravenous infusion at a dose of 1.1 × 10^14^ vg/kg. Immune responses to AAV9 and genotoxicity have been previously reported with systemic administration at high doses in some animal model studies (Kuzmin *et al*, 2021); for example, associated with ataxia and acute liver toxicity in non‐human primates and piglets at doses of 2 × 10^14^ vg/kg (Flotte & Buning, 2018; Hinderer *et al*, 2018). Rescue via our ubiquitous CAG promoter driven NDP construct implies that precise targeting of sites of NDP expression or OHCs is not necessary, so long as secreted NDP can reach the necessary target cells. This is consistent with rescue achieved in previous reports via ectopic overexpression of *Ndp* in the lens of transgenic mice (Ohlmann *et al*, 2005).

A limitation of the current study is that the treated mice were followed post injection for a maximum of 3 months of age. During which time no adverse health effects resulting from the treatment were observed. As Norrie disease manifests as late‐onset progressive hearing loss, the longer term outcome will be important to evaluate how sustained the treatment is and whether the effects diminish over time. After the later interventions (P21, P30), less cochlear cells were transduced and transgene expression was lower. It is possible that lower efficacy at later treatment timepoints is due to insufficient delivery to the target cells in mature animals and/or low responsiveness of aspects of the pathology already existing at the time of treatment. As systemic delivery of AAV risks side effects, direct delivery to the eye and ear may be more suitable for clinical translation, allow dosage optimisation and enable higher local levels of transduction. It will be important to perform a comparative study in the future by local delivery, compare the result with the current study and decide a possible route for human study. Longitudinal toxicology studies are needed to establish the safety of AAV9.NDP gene therapy. This proof‐of‐concept study demonstrates for the first time that the pathology in Norrie disease responds to gene replacement therapy and opens the way for targeted gene delivery to treat progressive hearing loss and retinal vascular exudation.

Such *NDP* gene delivery may be useful in alleviating ocular disease in FEVR (Wawrzynski *et al*, 2022) or in the future for prenatal gene therapy in cases of prenatal diagnosis of Norrie (Sisk *et al*, 2014). AAV9.NDP gene replacement may also have potential for treatment of peripheral vascular disease symptoms in Norrie patients. To our best knowledge, this is the first such application of systemic AAV9 delivery (Shibata *et al*, 2017) to treat a progressive hearing loss disorder.

## Materials and Methods

### Gene expression plasmids

The CAG>EGFP‐P2A‐NDP‐FLAG pAAV gene therapy construct was designed using vectorbuilder.com and the plasmid supplied by Cyagen. Plasmids expressing human FZ4, LRP6 and TSPAN12 (Chang *et al*, 2015) were provided by Prof Yvonne Jones (University of Oxford). M50 Super 8× TopFlash in pTA‐Luc vector (12456) and M51 Super 8× FopFlash in pGL3 vector (12457) were obtained from Addgene. All plasmids were expanded in *E. coli* using standard methods and purified using the Miraprep protocol (Pronobis *et al*, 2016). HEK293 cells were cultivated in DMEM‐high glucose medium (11965‐084, Gibco) with 10% FBS (A38401, Gibco) in cell culture incubators at 5% CO_2_ and 37°C. Cells were passaged at 1:5 ratio using 1× trypsin/EDTA (25300‐054, Gibco).

### TopFlash assay

HEK293 cells were plated at equal densities in 96‐well plates. The next day cells were transfected (Transfection mix: 40 μg of TopFlash plasmid, 10 μg of mCherry plasmid, and a combination of 10 μg of each of the *NDP* gene therapy construct and norrin receptor plasmids) using FuGENE^®^ HD reagent (2 μl FuGENE: 1 μg DNA) for 24 h followed by washing and replacement with tissue culture medium. And 5 mM LiCl treatment for 24 h was used as a positive control for β‐catenin activation. Following that, cells were assayed for β‐catenin activity using the Dual‐Luciferase^®^ Reporter Assay System (E1910, Promega), according to manufacturer's instructions. Induced luminescence and mCherry fluorescence as a transfection control were measured with a Luminometer Fluostar Optima (BMG Labtech).

### Western blotting

HEK293 cells were transfected with the *NDP* gene therapy construct as described above. And 48 h after the removal of transfection medium, cells were harvested in RIPA buffer containing protease inhibitor cocktail complete TM Mini (11836153001, Promega). Total protein was extracted and quantified by Bradford assay according to standard methods. Alternatively, tissue samples were snap‐frozen and stored at −80°C. Tissue was lysed in RIPA buffer containing protease inhibitor cocktail and dithiothreitol (DTT). Samples were diluted by mixing with 4× Laemmli sample buffer (BioRad) with or without 5% β‐mercaptoethanol, and/or heat inactivated at 75°C, or incubated at room temperature for 10 min, then maintained on ice. And 20–30 μg of protein was loaded per well on a 1 mm 12% SDS–PAGE gels and separated by electrophoresis (Mini‐PROTEAN, BioRad) followed by transfer onto 0.2 μm pore size nitrocellulose membrane (BioRad) in TransBlot semi‐dry transfer system. Membranes were washed in PBST, blocked in 5% non‐fat milk (Blotting‐Grade Blocker, BioRad) in PBST and probed using relevant primary antibodies: Anti‐GAPDH EMD Millipore MAB374 (1:1,000), Anti‐GAPDH Cell Signalling Technology #2118S (1:5,000), Anti‐FLAG eBioscience 14‐6681‐80 (1:1,000), Anti‐GFP Abcam Ab6662 (1:1,000), Anti‐NDP R&D Systems AF3014 (1:1,000), overnight at 4°C, followed by washes in PBST and incubation with secondary antibodies at 1:10,000 or 1:100,000 dilution for 2 h at room temperature. Membranes then were incubated with horseradish peroxidase substrate for 5 min (Clarity™ Western ECL Substrate, BioRad or SuperSignal™ West Atto Ultimate Sensitivity Substrate, Thermo Fisher) and imaged with ChemiDoc XRS+ system (BioRad).

### Virus production and packaging

The gene therapy construct was packaged into AAV capsids in the UCL NeuroGTx Vector Core Facility, using AAVpro® 293T (Takara Bio) cell culture and a triple plasmid transfection protocol and purification by iodixanol gradient ultracentrifugation. Virus titre was determined by qPCR using the linearised construct plasmid as a standard (ITR F: 5′GGAACCCCTAGTGATGGAGTT3′, R: 5′CGGCCTCAGTGAGCGA3′).

### Animal experiments

Animal studies were carried out after University College London and King's College London Ethics Review and in accordance with UK Home Office regulations and the UK Animals (Scientific Procedures) Act of 1986 under UK Home Office licences. Mice were kept at 12 h light, 12 h dark cycle and provided food and water *ad libitum*.

Mice carrying the *Ndp* ^*tm1Wbrg*^ (*Ndp* ^*−*^) allele were created by Prof W. Berger (Berger *et al*, 1996) and the 129 founder backcrossed to the C57BL/6 inbred strain for multiple generations. *Ndp* ^*−/−*^ females are known to be infertile (Luhmann *et al*, 2005b). The colony was maintained at UCL by crossing heterozygous *Ndp* ^*+/−*^ females with *Ndp* ^*Y/+*^ C57BL/6 males from Charles River. *Ndp* ^*Y/−*^ males and *Ndp* ^*−/−*^ females (*Ndp‐*KO) and littermate or age‐matched *Ndp* ^*Y/+*^ males or *Ndp* ^*+/+*^ females (WT) from the breeding colony were used in all experiments. *Ndp* ^*−/+*^ females were not used for experimental analysis. Genomic DNA was isolated from ear or tail biopsies and *Ndp* genotypes determined by PCR (MyFi Mix (BIO‐25050); F: 5′GTATTGCATCCATATTTCTTGG3′ R: 5′CTCTCCATCCCCTGACAAGGA3′, WT amplicon = 528 bp, KO amplicon ~1,500 bp). All animals used for electrophysiological analysis were also genotyped to determine the absence of the dominant *Cdh23* ^*753G*^ protective allele by PCR (F: 5′ATCATCACGGACATGCAAGA3′ R: 5′AGCTACCAGGAACAGCTTGG3′, amplicon size 315 bp) followed by a *HphI* (ThermoFisher) restriction digest. *HphI* digests only the 315 bp amplicon of the *Cdh23* ^*753G*^ allele into fragments of 93 and 222 bp, but not that of the *Cdh23* ^*753A*^ age‐related hearing loss (ahl1) allele carried by C57BL/6 (Suzuki *et al*, 2020). CBA/Ca mouse genomic DNA, which carry the *Cdh23* ^*753G*^ allele, were used as positive controls for the genotype assay.

### Treatment with the AAV9.NDP construct

An 8 μl of AAV9.NDP construct in PBS carrying 2.74 × 10^13^ vg/kg (vector genomes per kilogramme bodyweight) was injected in the superficial temporal vein of neonatal mouse pups (P2) using a 100 μl Hamilton syringe (“low,” L dose, P2‐L group). P21 and P30 mice were maintained at 38°C air temperature for 10 min to dilate the vasculature before injection of AAV9.NDP in PBS carrying either 5.45 × 10^12^ vg/kg (L) or 1.37–2.74 × 10^13^ vg/kg (“high,” H) dose into the tail vein (P21‐L, P21‐H and P30‐H groups). P2‐L and P21‐H groups received equivalent weight‐adjusted doses. Litters were genotyped prior to injection. Experimental groups were spread across litters: *Ndp*‐KO animals were injected with either the construct or a matched volume of PBS or left untreated; WT littermates were injected with a matched volume of PBS or left untreated. PBS injected and untreated animals were pooled for comparative analysis with each AAV9.NDP treatment group (P2‐L, P21‐L, P21‐H and P30‐H). Treated mice were monitored and weighed three times a week until P30, then once a week.

### Auditory electrophysiology

Auditory Brain Stem Responses (ABR), Distortion Product Otoacoustic Emissions (DPOAE) and Endocochlear Potential recordings (EP) were performed exactly as described previously (Bryant *et al*, 2022).

### Electroretinograms

Electroretinograms were performed in a similar fashion to published protocols (Ohlmann *et al*, 2005). Mice were dark‐adapted overnight for a minimum of 12 h and anaesthetised by isoflurane inhalation, and their pupils were dilated (1% tropicamide eye drops) and anaesthetised (proxymetacaine eye drops). The mouse was then connected to the OcuScience® HMsERG system according to the manufacturer's instructions. Mice were prepared for ERG's under a dim red light to avoid loss of dark adaptation. Single flash recordings were obtained under dark‐adapted (scotopic) conditions using stimulus intensities 1 mcds/m^2^, 3 mcds/m^2^, 10 mcds/m^2^, 30 mcds/m^2^, 0.1 cds/m^2^, 0.3 cds/m^2^, 1 cds/m^2^, 3 cds/m^2^, 10 cds/m^2^ and 25 cds/m^2^. Ten responses were averaged at each intensity.

### RNA extraction

Cochleas were isolated from surrounding tissue and the vestibule and snap‐frozen. Retinas were dissected from the eye in cold PBS and snap‐frozen. Total RNA was extracted using a modification of a published protocol (Vikhe Patil *et al*, 2015) (TRI Reagent® 93289‐25ML Sigma‐Aldrich, DirectZol kit). RNA was eluted in 40–50 μl of nuclease‐free water and analysed using the by NanoDrop™ 2000 (Thermo Scientific) and Agilent Bioanalyzer platforms.

### Gene expression analysis

Whole transcriptome analysis was performed using strand‐specific RNA sequencing with poly‐A selection on the Illumina Nova‐seq, with a target library size of 20 million paired end 150 bp reads per sample. Differential expression analysis was performed in edgeR package. To filter out low expression genes, genes with CPM < 0.5 (or < 10 counts) and present in less than two samples were removed from the analysis. Differentially expressed genes with a *P*‐adjusted value of < 0.05 were considered to be statistically significant. Gene set enrichment analysis (GSEA; Subramanian *et al*, 2005) was used to profile the expression of genes related to vascular barrier function (Daneman *et al*, 2010) in WT versus *Ndp*‐KO RNAseq data. For RT‐PCR analysis, cDNA was synthetised from 100 ng RNA using RevertAid H Minus First Strand cDNA Synthesis kit (K1631) with random hexamers according to manufacturer's instructions. cDNA equivalent to 1 ng of RNA per reaction was used for gene expression analysis with PowerSYBR® Green PCR Master mix (436759) and relevant primers (Table 1).

**Table 1:** PCR primers.

Gene/primer ID	Forward	Reverse	Amplicon, bp
*Abcb1a*	GCGACTCCGATACATGGTTT	ACCCTGTAGCCCCTTTCACT	134
*Actin‐b*	TGTTACCAACTGGGACGACA	CTGGGTCATCTTTTCACGGT	139
*Ceacam16*	ATGAAAATGCCATTGACCTGGTA	TGTGTCCGTAGCCCACCT	376
*Common NDP/Ndp*	GATTCTATCAGTCACCCA	AGTGACAGGAGAGGATGT	247
*Cldn5*	TTAAGGCACGGGTAGCACTCACG	TTAGACATAGTTCTTCTTGTCGTAATCG	320
*Clu*	CCTTCCAGTCGAAGATGCTC	TGTGATGGGGTCAGAGTCAA	209
*EGFP*	AGTCCGCCCTGAGCAAAGA	TCCAGCAGGACCATGTGATC	50
*mNdp*	CCCACTGTACAAATGTAGCTCAA	AGGACACCAAGGGCTCAGA	92
*Nr1h4*	AGGGAGAAAACGGAACTCACGG	CCGCCGAACGAAGAAACATGG	283
*Plvap*	GTGGTTGGACTATCTGCCTC	ATAGCGGCGATGAAGCGA	188
*Slc7a1*	TTCGGTTATGGGATCTGGCACAGT	TTTGCACTGGTCCAAGTTGCTGTC	87
*Slc7a5*	CTGGTCTTCGCCACCTACTT	GCCTTTACGCTGTAGCAGTTC	128
*Sox17*	GCACAGCAGAACCCAGATCT	CCGGTACTTGTAGTTGGGGT	156

Publicly available scRNAseq data sets of the mouse retina and cochlea (GSM6513065, GSM3580725, GSM3580727, GSM5124291, GSM5124292, GSM5124293, GSM5124294, GSM5124299, GSM5124300, GSM5124301, and GSM5124302; Heng *et al*, 2019; Milon *et al*, 2021; Dong *et al*, 2022) were obtained from the GEO database. Data were analysed using the Seurat package. Only cells expressing more than 200 genes and fewer than 8,000 and with mitochondrial gene percentages < 60% were used for analysis. Cells from biological replicates of the same tissue were integrated with the Seurat standard protocol, and the effect of mitochondrial genes was regressed out using “ScaleData” function. Clusters were generated using the “FindClusters” function with a resolution of 0.6. The top markers for each cluster were identified using the “FindConservedMarkers” function and used to assign cell identities to clusters. Full lists of the top marker genes used for cell assignment can be found in Datasets EV1 and EV2. Expression of *Ndp* and other genes of interest were plotted as DotPlots or UMAP plots.

### Tissue processing and histology

Eyes were isolated and fixed in 4% paraformaldehyde (PFA) for 60–90 min followed by multiple washes with PBS. For cryosectioning, eyes were sequentially equilibrated in 15% and 30% sucrose in PBS, embedded in OCT medium (ThermoFisher), snap‐frozen in a dry‐ice isopentane slurry and stored at −80°C. Retinal sections were cut at 12 μm thickness on a Leica cryostat and mounted on SuperFrost Plus glass slides (ThermoFisher). Retinal whole mount preparations were made by removing the sclera, choroid and RPE from the posterior segment of the fixed eye and making five radial incisions into the retina with the longest incision marking the ventral retina. Cochlea were isolated and dissected out of the auditory bulla. The cochlear apex and oval and round windows were opened and 1 ml 4% PFA injected through the round window. Fixation was continued in 4% PFA for 2 h, followed by decalcification in 4% EDTA in PBS (w/v), pH 7.4, for 72 h and multiple washes in PBS. Cochlear whole mount preparations were made by removing the otic capsule and separating the lateral wall and modiolus by cutting beneath the stria vascularis. Alternatively, cochlea were embedded in 4% low melting grade agarose (Invitrogen) in PBS and stored at 4°C; 150–200 μm thick cross‐sections were cut using a vibratome (Leica) and stored in PBS at 4°C until further processing. For cryosectioning, cochlea were sequentially equilibrated in 15 and 30% sucrose in PBS, a 1:1 mixture of 30% sucrose in PBS with OCT medium (ThermoFisher), embedded in OCT medium, snap‐frozen in cold isopentane and stored at −80°C. Sections were cut at 10 μm thickness and mounted on SuperFrost Plus glass slides (ThermoFisher).

### Immunohistochemistry

Tissue samples were incubated in permeabilisation/blocking solution (5% FBS, 1% BSA in PBS) containing 0.1% (tissue sections) or 0.5% (wholemounts) Triton X‐100. Samples were incubated with primary antibodies diluted in permeabilisation/blocking solution overnight at 4°C, washed with PBS, incubated for 2 h in secondary antibodies at room temperature, incubated in 1:2,000 DAPI in PBS for 10 min at room temperature, washed with PBS and mounted with Prolong Diamond (P36970, Invitrogen). Specifically, for anti‐NDP staining in the cochlea, permeablisation/blocking solution contained 4% Triton X‐100, and antibody incubations were at 37°C for 4 h (primary) and 2 h (secondary). *Primary antibodies*: Table 2. *Secondary antibodies*: Anti‐mouse IgG(H + L) Alexa Fluor 488 (Life Technologies A11001 1:500), Anti‐mouse IgG(H + L) Alexa Fluor 594(Life Technologies A21203 1:500), Anti‐rat IgG(H + L) Alexa Fluor 647 (ThermoFisher A21247 1:250), Anti‐rabbit IgG(H + L) Alexa Fluor 488 (Life Technologies A21206 1:250), Anti‐rabbit IgG(H + L) Alexa Fluor 568 (ThermoFisher A11036 1:250). *Markers*: Alexa Fluor 647 phalloidin conjugate (Life Technologies A22287 1:200), Alexa Fluor TM Plus 750 phalloidin conjugate (Invitrogen A30105, 1:500), Alexa Fluor 594 isolectin GS‐IB4 conjugate (Life Technologies I21413, 1:100), Alexa Fluor 647 isolectin GS‐IB4 conjugate (Invitrogen I32450, 1:100). Images were taken on a fluorescence microscope (Zeiss Observer, Olympus IX71) or spinning disk confocal (Yokogawa, CSU22) as stacks or tiled scans.

**Table 2:** Antibodies for immunostaining.

Antibody	Host species	Type	Catalogue number (RRID)	Company	Dilution
Anti‐endomucin	Rat	Monoclonal IgG1	Sc53941 (AB_2100038)	SantaCruz	1:100
Anti‐Myo7a	Rabbit	Polyclonal	25‐6790 (AB_10015251)	Proteus	1:200
Anti‐Plvap	Rat	Monoclonal	553849	BD Biosciences	1:100
Anti‐Claudin5	Rabbit	Polyclonal	34‐1600	Invitrogen	1:500
Anti‐NDP	Goat	Polyclonal	AF3014	R&D Systems	1:100
Anti‐GFAP	Rabbit	Polyclonal	AB5804	EMD Millipore Corp	1:500

Conjugated primary antibodies	Host	Type	Catalogue number (RRID)	Company	Dilution
Anti‐GFP‐FITC	Goat	Polyclonal	Ab6662	Abcam	1:200
Anti‐TUBB3	Mouse	Monoclonal	801207	BioLegend	1:500
Anti‐GFP‐488 Alexa Fluor TM 488	Rabbit	Polyclonal	A21311	Invitrogen	1:200

Fluorescent tag conjugated probes	Type/IgG	Host	Catalogue number (RRID)	Company	Dilution
Alexa Fluor 647 phalloidin conjugate	–	–	A22287 (AB_2620155)	Life Technologies	1:200
Alexa Fluor TM Plus 750 phalloidin conjugate	–	–	A30105	Invitrogen	1:200
Alexa Fluor 594 isolectin GS‐IB4 conjugate	–	–	I21413 (AB_2313921)	Life technologies	1:100
Alexa Fluor 647 isolectin GS‐IB4 conjugate	–	–	I32450	Invitrogen	1:100

Secondary antibody	Type/IgG	Host	Catalogue number (RRID)	Company	Dilution
Anti‐mouse Alexa Fluor 488	IgG(H+L)	Goat	A11001 (AB_2534069)	Life Technologies	1:500
Anti‐mouse Alexa Fluor 594	IgG(H+L)	Donkey	A21203 (AB_141633)	Life Technologies	1:500
Anti‐rat Alexa Fluor 647	IgG(H+L)	Goat	A21247 (AB_141778)	ThermoFisher	1:250
Anti‐rabbit Alexa Fluor 488	IgG(H+L)	Donkey	A21206 (AB_2535792)	Life Technologies	1:250
Anti‐rabbit Alexa Fluor 568	IgG(H+L)	Goat	A11036 (AB_10563566)	ThermoFisher	1:250

### Branch point analysis

Branch points of strial capillaries were manually quantified from low magnification images of lateral wall wholemount preparations using ImageJ. Vascular “intersection” points, which had three branches connected, were considered branch points.

### Hair cell quantification

Using low magnification images of wholemount preparations, each organ of Corti sample was mapped using the Measure_line macro for ImageJ (Redmond *et al*, 1993) and divided into 24 equal pieces. Using a custom‐made ImageJ macro, 200‐μm long rectangular images were sampled from each piece and MyoVIIA‐positive hair cells counted using local maxima detection. Empty slots left by dead cells were counted manually. Percentage surviving cells were calculated as surviving/(dead + surviving) × 100%. Values from three adjacent regions were averaged giving a total of eight regions per organ of Corti. Data from treated and control groups were analysed using two‐way ANOVA and Tukey's *post hoc* tests for multiple comparisons (GraphPad PRISM v7.0).

### Statistical analysis

The number of mice (*n*) used for each experiment is stated in the legends. Error bars always represent standard deviation (SD). *n* indicates biological replicates throughout the study. Animals and tissue sample treatment groups were not blinded for analysis except for auditory electrophysiology where genotypes were masked. qRT PCR analysis and animal physiology experiments used samples sizes of at least *n* = 6 biological replicates per group. Samples were not excluded from analyses, except when quality control genotype tests, which were conducted post‐mortem, failed to confirm expected genotype of experimental animal. This criterion was pre‐established. Some samples used for immunostaining analysis were omitted due to high background or damage during dissection. For RNAseq analysis, RNA samples with RIN values < 7.0 were excluded. Data were analysed using statistical tests as appropriate for each data set. Normality of data (Gaussian distribution) was assessed by running a set of normality tests. If normality was passed, data were analysed with Student's *t*‐test and one‐ or two‐way ANOVAs with Dunnett's or Tukey's or Sidak's or other *post hoc* tests, as indicated. The type of statistical tests, significance levels (*P*‐values) and *post hoc* analysis are presented in the respective figure legends. *P* value < 0.05 was considered significant. Data and graphs were analysed using GraphPad PRISM 7.

## Author contributions

**Valda Pauzuolyte:** Conceptualization; formal analysis; investigation; methodology; writing – original draft; writing – review and editing. **Aara Patel:** Data curation; formal analysis; investigation; methodology; writing – review and editing. **James R Wawrzynski:** Formal analysis; investigation; methodology; writing – original draft. **Neil J Ingham:** Formal analysis; investigation; methodology; writing – review and editing. **Yeh Chwan Leong:** Formal analysis; methodology. **Rajvinder Karda:** Methodology. **Maria Bitner‐Glindzicz:** Conceptualization; supervision. **Wolfgang Berger:** Resources. **Simon N Waddington:** Methodology. **Karen P Steel:** Formal analysis; methodology; writing – review and editing. **Jane C Sowden:** Conceptualization; resources; formal analysis; supervision; funding acquisition; methodology; writing – original draft; project administration; writing – review and editing.

## Disclosure and competing interests statement

A patent application relating to this work has been filed by UCLB: application number 2214972.8.

## For more information

- https://norriedisease.org.uk
- https://www.ncbi.nlm.nih.gov/books/NBK1331/
- https://www.omim.org/entry/300658

## Supporting information



Appendix

Expanded View Figures PDF

Dataset EV1

Dataset EV2

Dataset EV3

Source Data for Expanded View

PDF+

Source Data for Figure 1

Source Data for Figure 2

Source Data for Figure 3

Source Data for Figure 4

Source Data for Figure 5

Source Data for Figure 6

Source Data for Figure 7

Source Data for Figure 8

## Data Availability

The datasets produced in this study are available in the following databases: [RNAseq data]: [ArrayExpress collection in BioStudies] [E‐MTAB‐12703] [Bl6 Ndp‐KO systemic gene therapy whole cochlea samples] (https://www.ebi.ac.uk/biostudies/arrayexpress/studies/E-MTAB-12703).
