# Supplementary material for: Systemic gene therapy rescues retinal dysfunction and hearing loss in a model of Norrie disease
Source: EMBO Mol Med. 2023 Aug 29;15(10):e17393. doi: 10.15252/emmm.202317393 (PMC10565640; doi:10.15252/emmm.202317393)
Supplement: Supplementary file 1 — Appendix [file EMMM-15-e17393-s015.pdf]

Appendix

Table of Contents

Table of Contents	Page
Appendix Figure S1	2
Appendix Figure S2	4
Appendix Figure S3	6
Appendix Figure S4	8
Appendix Figure S5	10
Appendix Figure S6	12

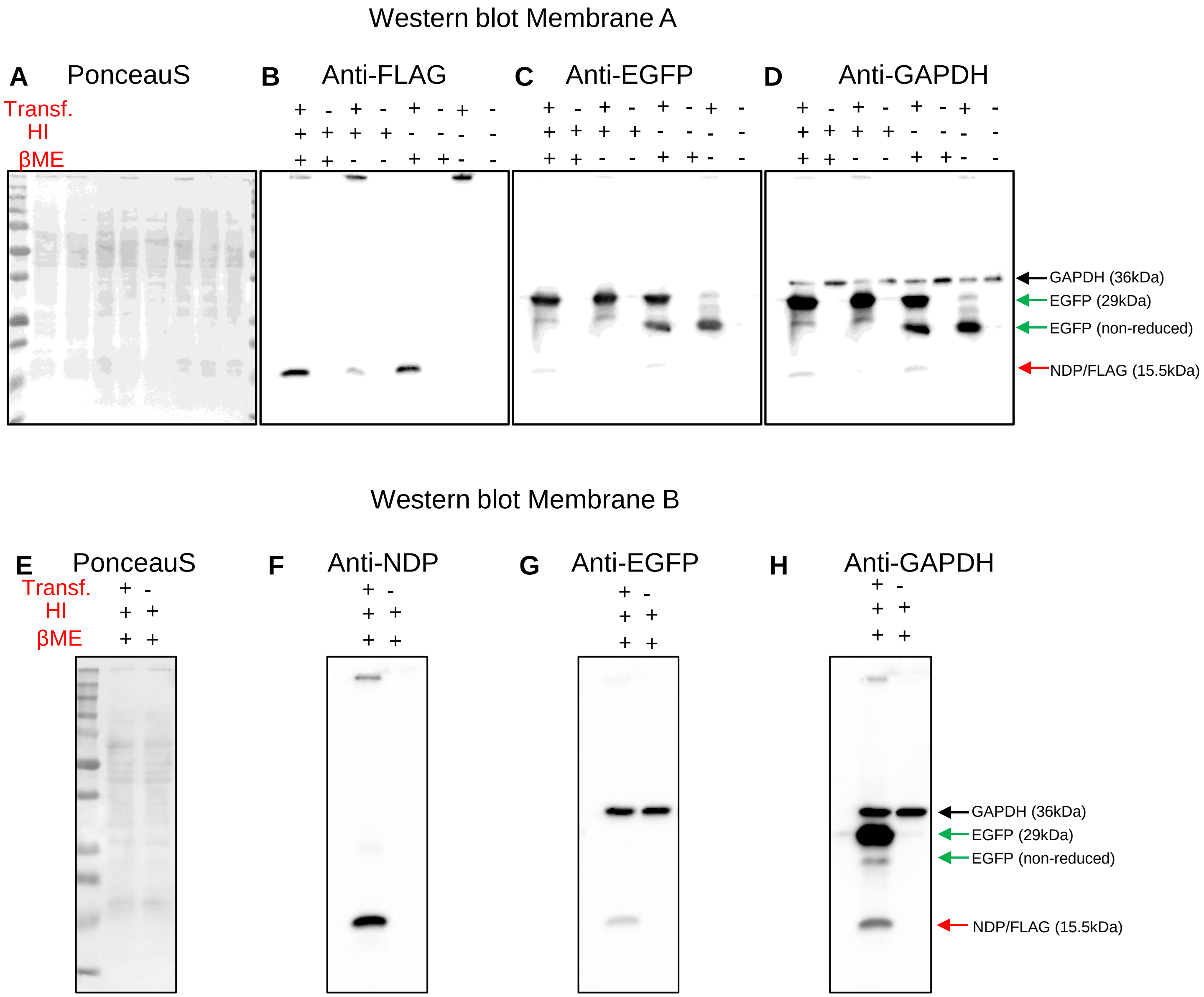

Appendix Figure S1

Appendix Figure S1: Detection of the transgenic proteins by Western blot in lysates of HEK293 cells, transfected with AAV9.NDP construct. Samples processed with (+) or without (-) HI: Heat inactivation, βME: 5% β-mercaptoethanol reduction, Tr: Construct transfection. Molecular weight ladder indicated at left hand side.

(A-D) Membrane A sequentially stained with PonceauS, Anti-FLAG (15 kDa NDP monomer), Anti-EGFP (~29 kDa band in non-reducing conditions and 25 kDa in reducing conditions), Anti-GAPDH.

(E-H) Membrane B sequentially stained with PonseauS, Anti-NDP (15 kDa NDP monomer), Anti-EGFP (~25 kDa band in non-reducing conditions and 29 kDa in reducing conditions), Anti-GAPDH.

Note that Anti-FLAG or Anti-NDP staining do not colocalize with Anti-GFP. The peptide molecular weights are of the predicted sizes, indicating cleavage of the P2A linker and absence of fusion protein. Membranes were stripped and stained sequentially. Red boxes indicate cropped regions in Figure 1C.

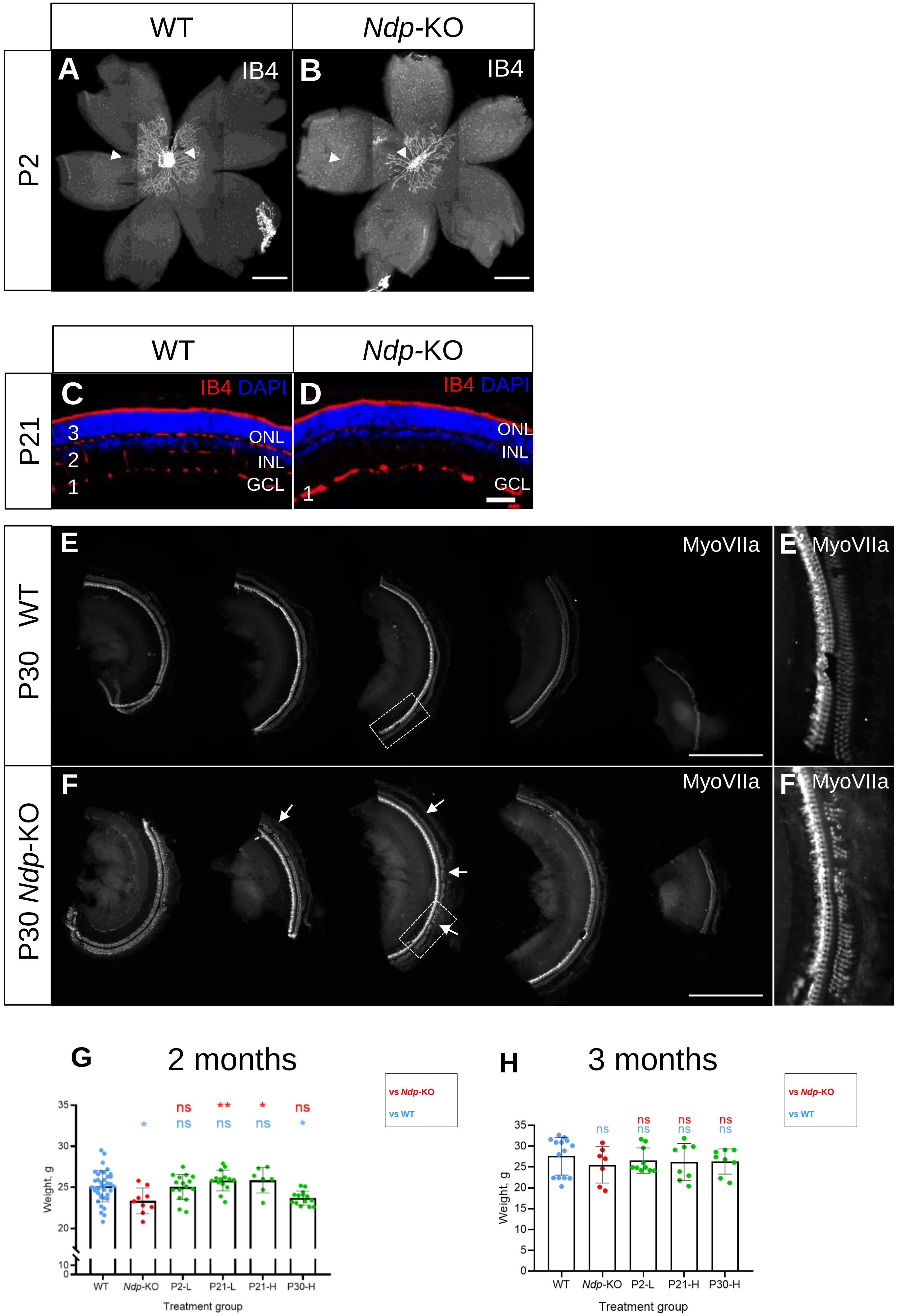

Appendix Figure S2

Appendix Figure S2: *Ndp*-KO phenotypes at time of AAV9.NDP treatment and weights of untreated control WT and *Ndp*-KO mice and treated *Ndp*-KO mice after AAV9.NDP administration.

(A, B) Flatmounts of WT and *Ndp*-KO retinas at P2, vasculature immunostained with anti- isolectin-B4 (IB4). Scale bar = 500 µm.

(C, D) Cryosections of WT and *Ndp*-KO retinas at P21, vasculature immunostained with isolectin-B4 (IB4). Scale bar = 50 µm. GCL – ganglion cell layer, INL – inner nuclear layer, ONL – outer nuclear layer. Numbers 1, 2, 3 label the vascular plexuses.

(E, F) Organ of Corti wholemounts showing limited outer hair cell death in *Ndp*-KO cochleas (F) as compared to WT (E). n(WT) = 3, n(*Ndp*-KO) = 3.

(G, H) Weights of *Ndp*-KO mice after treatment at P2 (P2-L), P21 (P21-L and P21-H) and P30 (P30-H) at 2 months (G, males) and 3 months (H, males and females). Data are shown as mean ± SD; one-way ANOVA with Sidak's *post hoc* test.

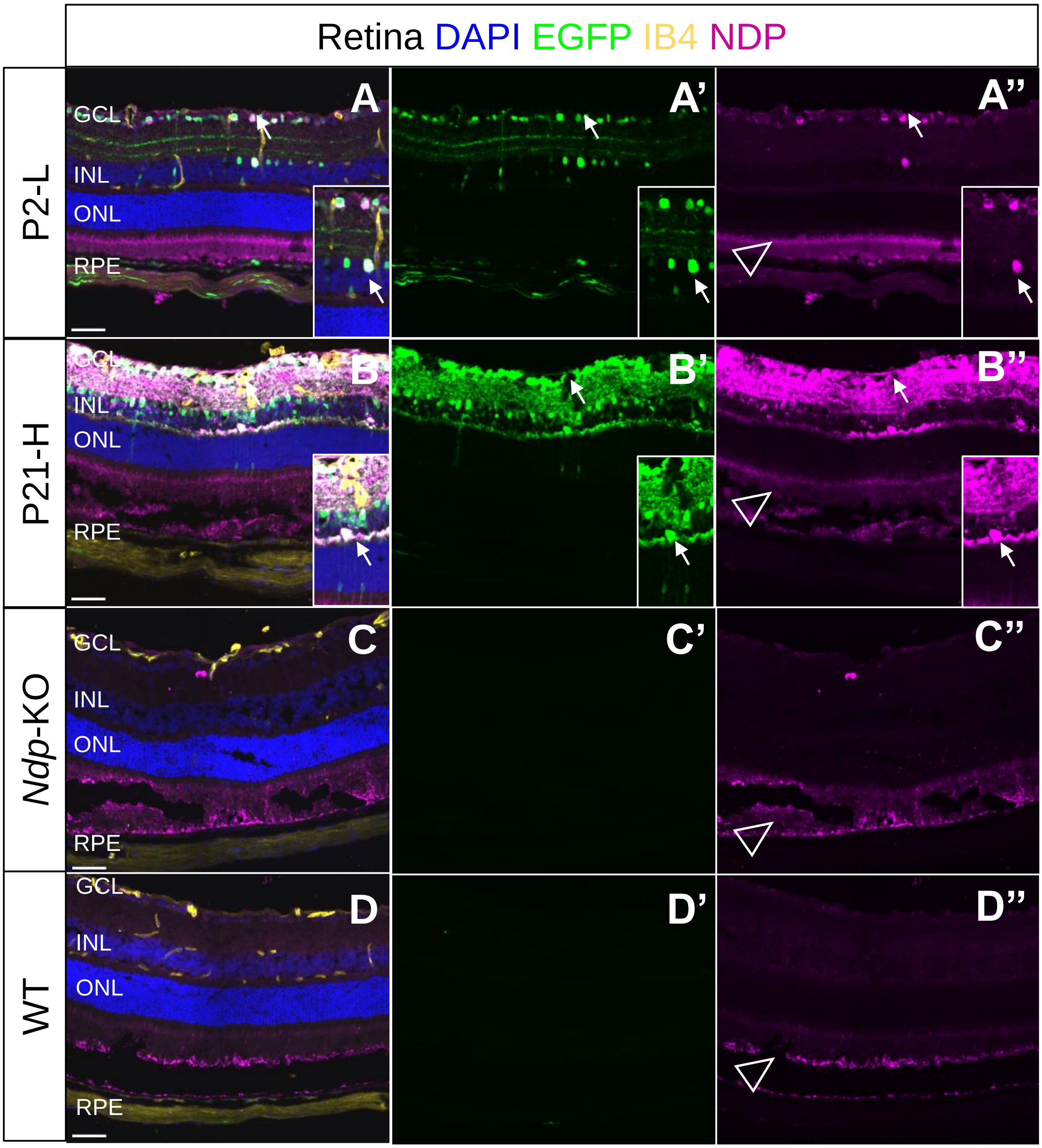

Appendix Figure S3

Appendix Figure S3: AAV9.NDP vector transduction and NDP immunohistochemistry in the retina.

A-D. *Ndp*-KO retinal sections at 2 months after AAV9.NDP treatment: P2-L group, n = 4; P21-H group, n = 4, WT and *Ndp*-KO controls. Immunohistochemistry: anti-GFP antibody (EGFP, green), anti-NDP (NDP, magenta), IB4 (vessels, yellow), DAPI (nuclei, blue). GCL – ganglion cell layer, ONL – outer nuclear layer, INL – inner nuclear layer. White arrows indicate transduced cells. Open arrowhead indicates non specific background signal in photoreceptor segments using anti-NDP-antibody. Scale bar 50µm

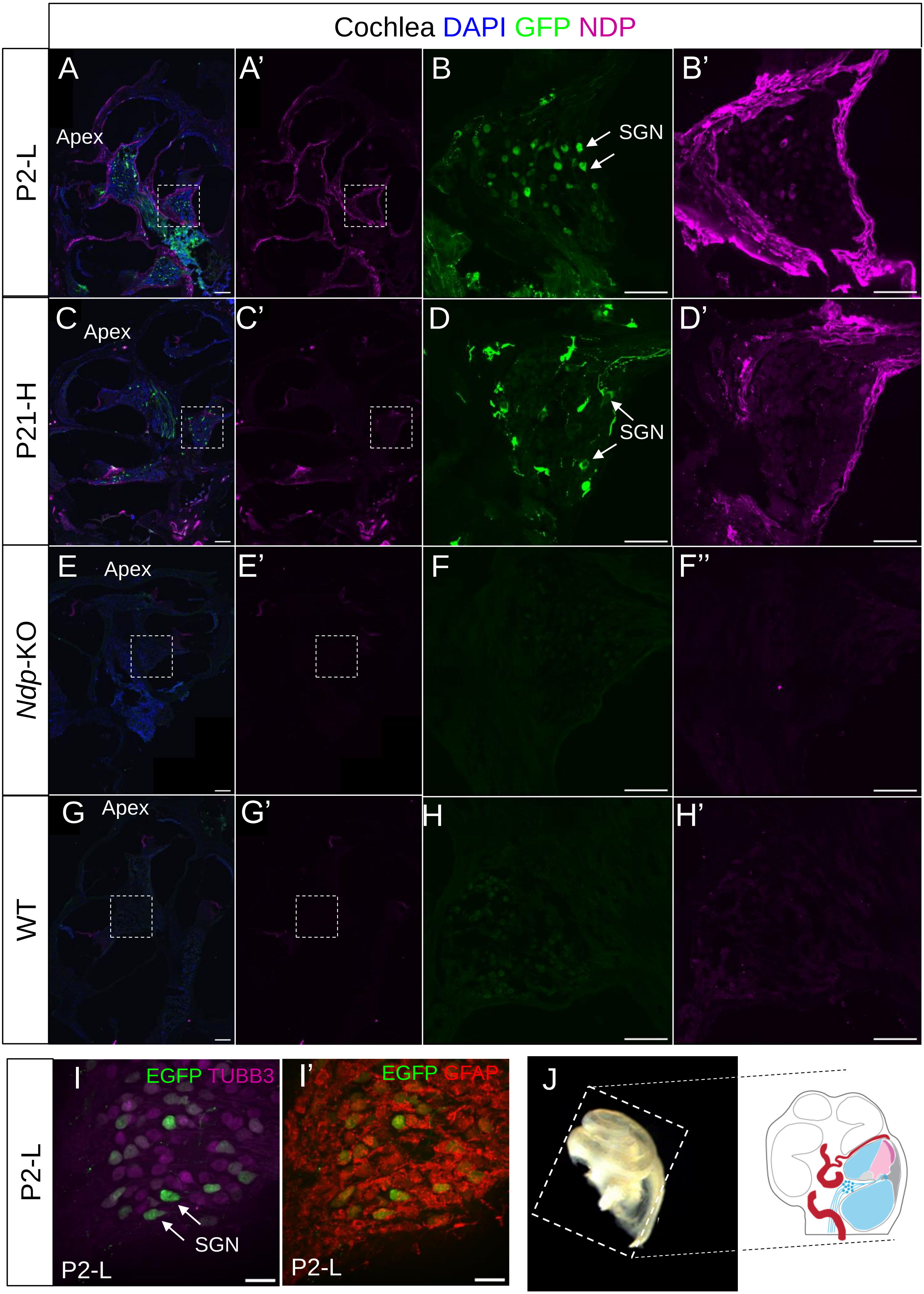

Appendix Figure S4

Appendix Figure S4: AAV9.NDP vector transduction and NDP immunohistochemistry in the cochlea.

(A-H) *Ndp*-KO cochlea sections at 3 months after AAV9.NDP treatment: P2-L, P21-H and untreated *Ndp*-KO and WT cochleas: anti-NDP (NDP, magenta), anti-GFP antibody (EGFP, green), DAPI (nuclei, blue). Boxed GFP /NDP labelled spiral ganglia region shown at higher magnification in B, D, F, H. Immunostaining signal is highest in the P2-L group. Scale bar 100 µm in A, C, E, G and 50 µm in B, D, F, H.

(I, I') Sections through a P2-L cochlea at 3 months immunostained for EGFP (green), TUBB3 (magenta) and GFAP (red) showing that spiral ganglion neurons are transduced. Scale bar: 20 µm. Arrows indicate spiral ganglion neurons, SGN.

(J) Schematic showing the plane of section through the cochlea. Lilac indicates lateral wall. Pink indicated the scala media. Blue indicated the scala typani and scala vestibuli. Spiral ganglion neurons also drawn in blue at the centre of the cochlea. Light grey is the spiral limbus. Figure EV3J reused in Appendix 1, Fig. S4A

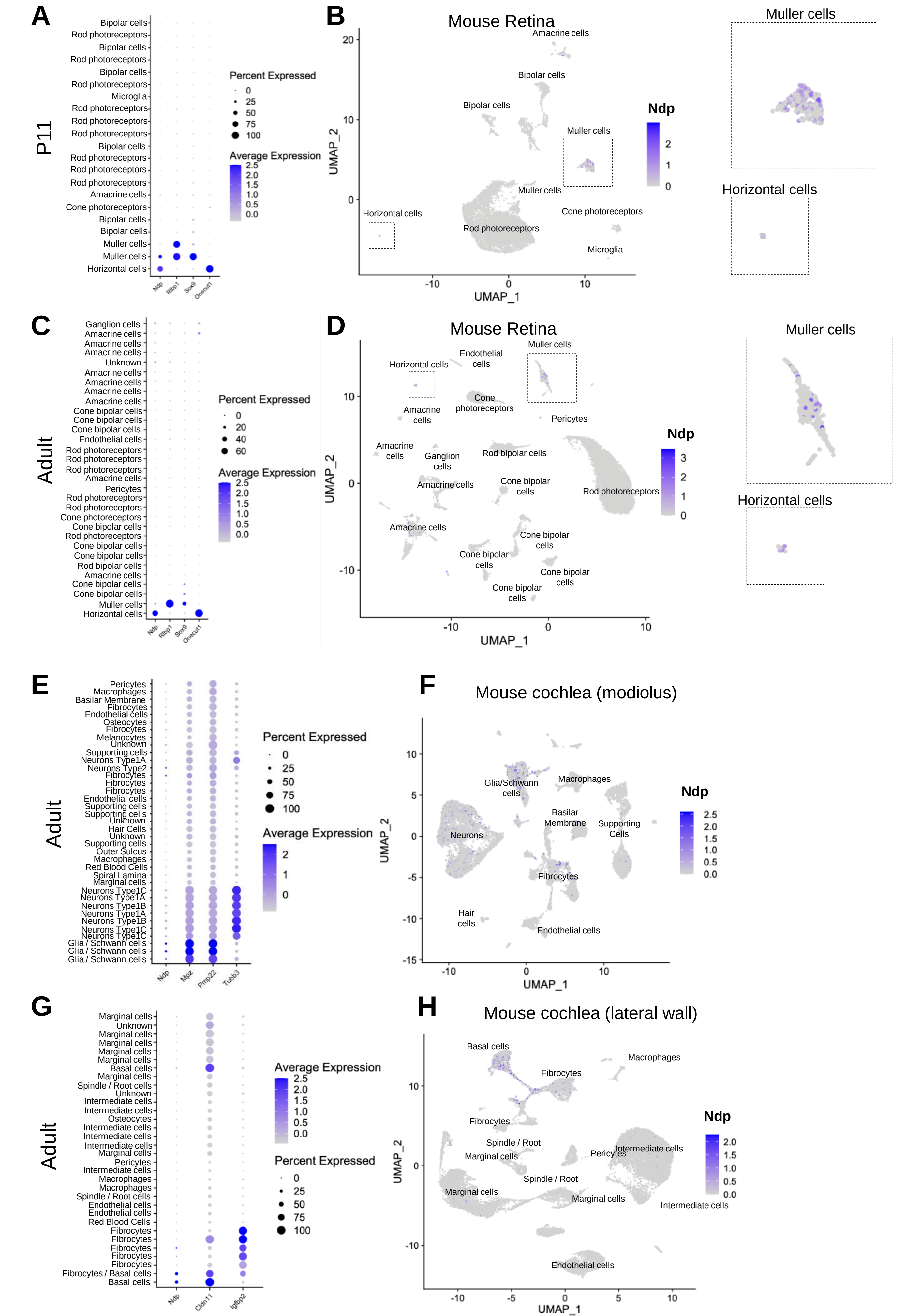

Appendix Figure S5

Appendix Figure S5: Analysis of single-cell transcriptomic atlases of the mouse retina and cochlea showing endogenous sites of *Ndp* expression

(A-D) Dot-plot and UMAP plot showing the expression of *Ndp* in the P11 retina (A-B) and in the adult mouse retina (C-D). Note the strongest expression in clusters expressing Müller glial markers *Rlbp1* and *Sox9* and horizontal cell marker *Onecut1.*

(E-F) Dot-plot and UMAP plot showing the expression of *Ndp* in the modiolus of the adult mouse cochlea. Note strongest expression in clusters expressing glial markers *Mpz* and *Pmp22*; little expression in neurons (spiral ganglion neurons) and none in hair cells.

(G-H) Dot-plot and UMAP plot showing the expression of *Ndp* in the lateral wall of the adult mouse cochlea. Note strongest expression in clusters expressing basal cell marker *Cldn11* and fibrocyte marker *Igfbp2*.

Data source: P11 retina: GSM6513065; Adult retina: GSM3580725, GSM3580727; Adult modiolus: GSM5124291, GSM5124292, GSM5124293, GSM5124294; Adult lateral wall: GSM5124299, GSM5124300, GSM5124301, GSM5124302.

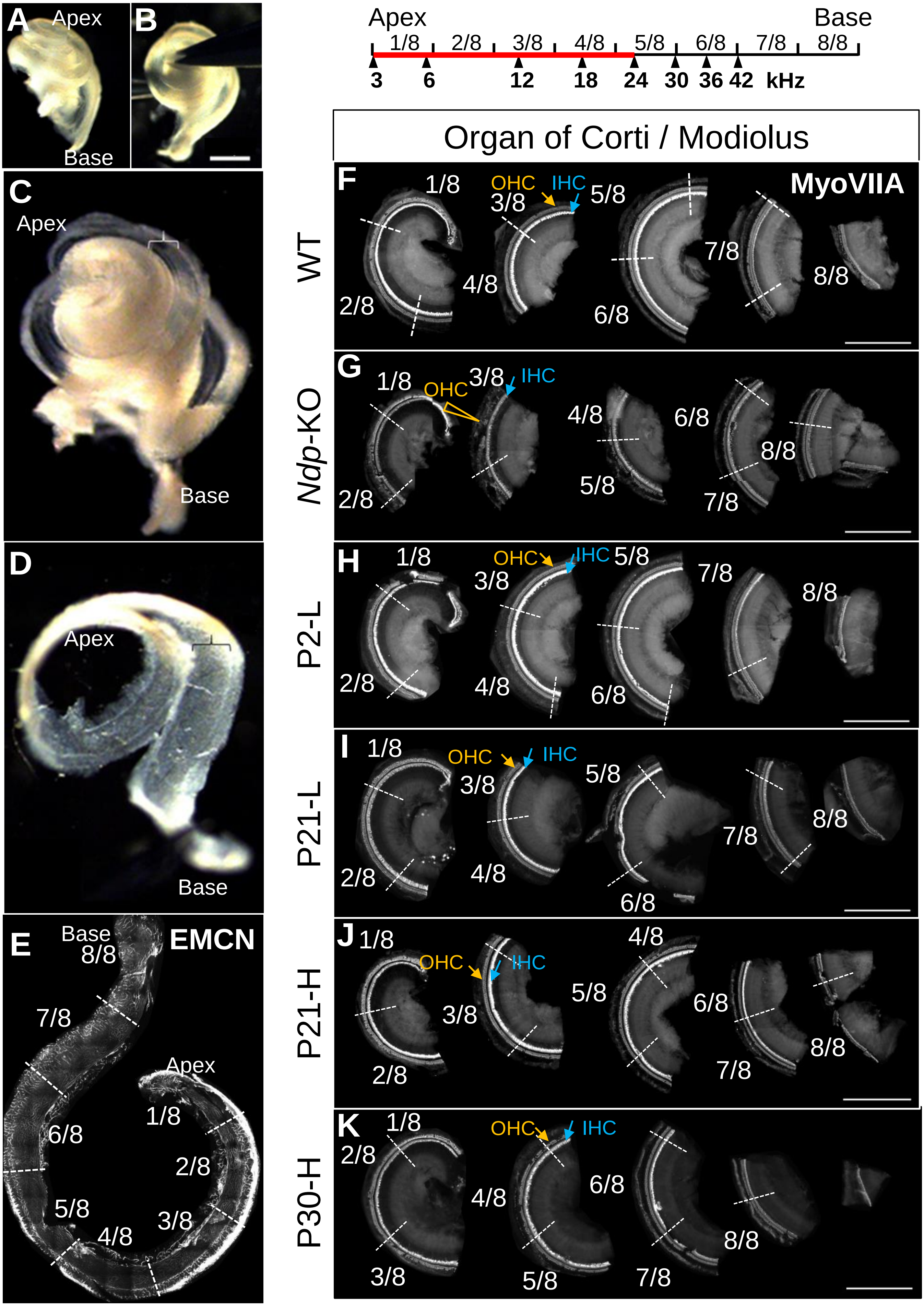

Appendix Figure S6

Appendix Figure S6: **Hair cell survival along the apex to base axis in organ of Corti whole mounts at 2 months.**

A-E. Schematic of regions of the cochlea 1-8 along the apex to base axis and corresponding frequencies are shown at the top of the figure. Mapped regions 1-8 used for quantification.

F-K. Hair cell survival in regions 1-8 along the tonotopic apex to base axis of the organ of Corti from different treatment groups. MyoVIIA immunostaining (white) of outer hair cells (OHC) and inner hair cells (IHC). (F) WT n = 3, (G) *Ndp*-KO n = 6, (H) P2-L n = 5, (I) P21-L n = 3, (J) P21-H n = 6, (K) P30-H n = 8. Scale bar = 500 µm.
