# Supplementary material for: Systemic gene therapy rescues retinal dysfunction and hearing loss in a model of Norrie disease
Source: EMBO Mol Med. 2023 Aug 29;15(10):e17393. doi: 10.15252/emmm.202317393 (PMC10565640; doi:10.15252/emmm.202317393)
Supplement: Supplementary file 2 — Expanded View Figures PDF [file EMMM-15-e17393-s006.pdf]

Expanded View Figures

Figure EV1. AAV9.NDP vector transduction in the cochlea and retina.

A–E Organ of Corti and lateral wall wholemounts stained with anti-GFP antibody. (A-A″) Untreated WT cochlea; (B-B″) P2-L dose; (C-C″) P21-L dose; (D-D″) P21-H dose; (E-E″) P30-H dose. Scale bars = 500 μm (A-E), 500 μm (A′-E′), enlarged view of boxed region shown in A″-E″. SGN, spiral ganglia region; arrowheads, fibrocyte shaped cells in the lateral wall.

F P30-H dose; cochlea stained with anti-MyoVIIa antibody showing that hair cells are not transduced. Scale bar 100 μm.

G, H EGFP immunostaining at 2 months in retinal cryosections after AAV9.NDP treatment at P2 (G) and P21 (H); optic nerve: o.n. Arrows indicate transduced region of the retina; arrowheads indicate transduced cells in the RPE. Scale bar 500 μm.

Source data are available online for this figure.

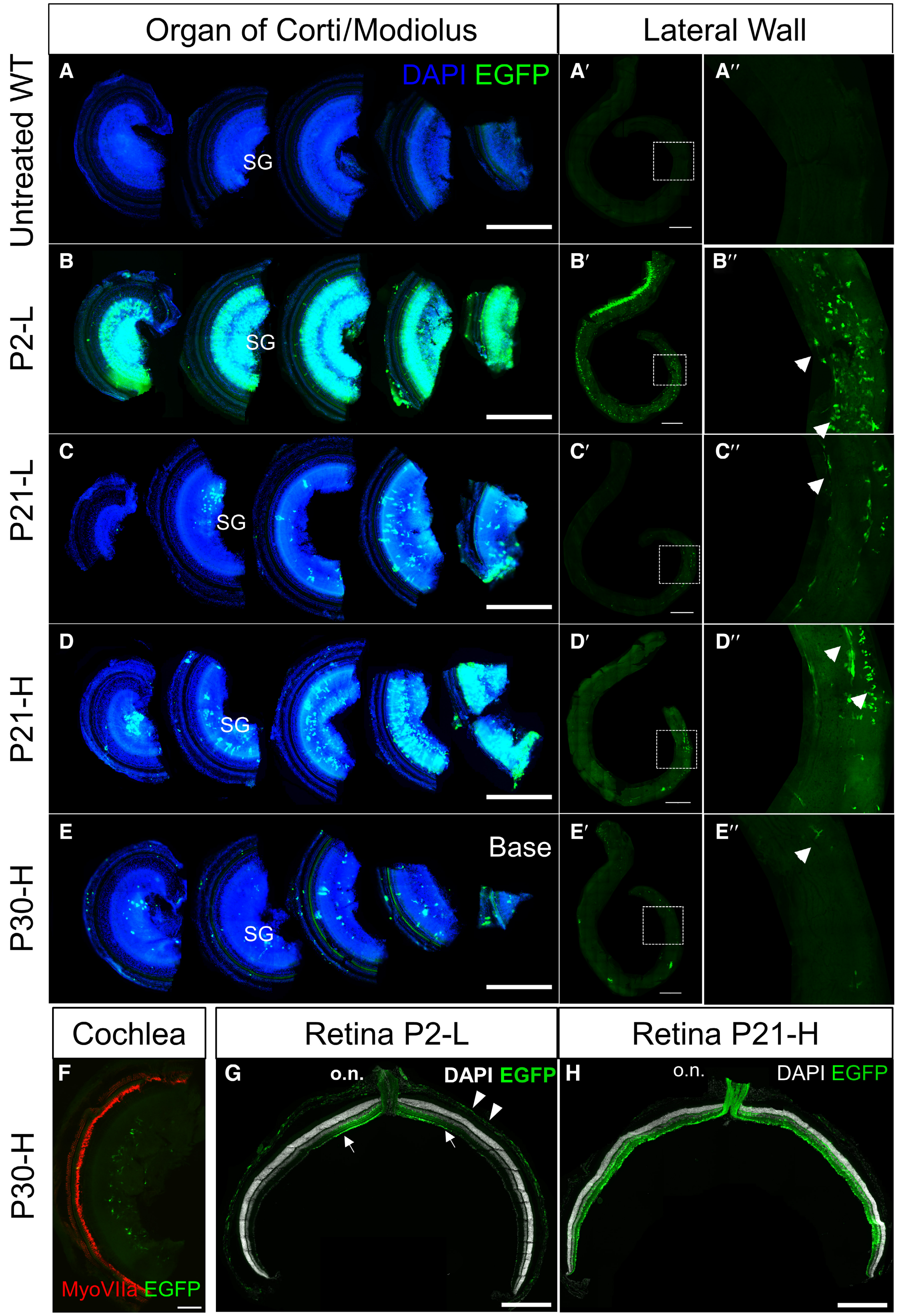

Figure EV1.

© 2023 The Authors

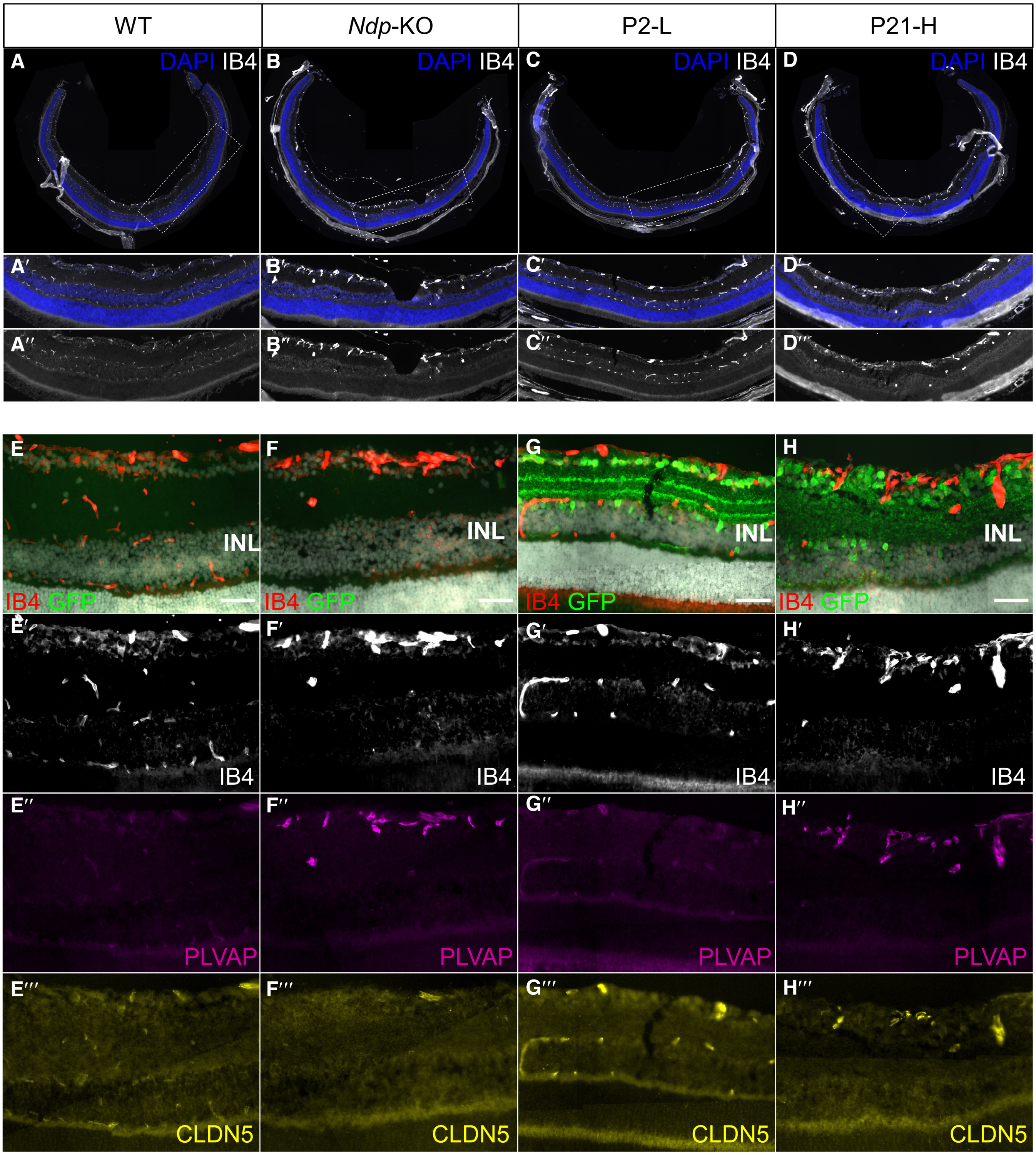

Figure EV2. Effects of AAV9.NDP treatment on the retinal vessel morphology and barrier proteins.

A–H Retinal cryosections at 2 months stained with IB4 (A–D, E–G, E–G′), anti-PLVAP and anti-CLDN5 antibodies (E″–G″) and anti-EGFP antibody (E–H). Note the presence of three layers of vessels on WT (A-A″, E-E′) and P2-L (C-C″, G-G′) groups and only one layer in *Ndp*-KO (B-B″, F-F′) and P21-H (D-D″, H-H′) groups. CLDN5 expression is visible in vessels in WT (E‴), P2-L (G‴) and P21-H (H‴) groups. Scale bar 50 μm (E–H).

Source data are available online for this figure.

© 2023 The Authors

Figure EV3. Electroretinograms showing effects of early and late treatments.

A Representative ERG traces in response to flashes of light of increasing intensity (an average of 10 flashes shown for each trace).
B, C Flash ERG, ratio of b-wave to a-wave amplitude for P2-L and P21-H treatment groups.
D, E Flash ERG, a-wave amplitudes for P2-L and P21-H treatment groups.

n = biological replicates. Data information: *n* (WT) = 10, *n* (*Ndp*-KO) = 10, *n* (P2-L) = 7, *n* (P21-H) = 10. Data are shown as mean ± SD. Statistical analysis was performed by one-way ANOVA with Sidak's *post hoc* test, comparing each treatment group with WT (blue asterisks) and *Ndp*-KO (red asterisks), between WT and *Ndp*-KO (black asterisks). *Post hoc* test values: *$P \leq 0.05$, **$P \leq 0.01$, ***$P \leq 0.001$, ****$P \leq 0.0001$: ns, not significant.
Source data are available online for this figure.

 © 2023 The Authors

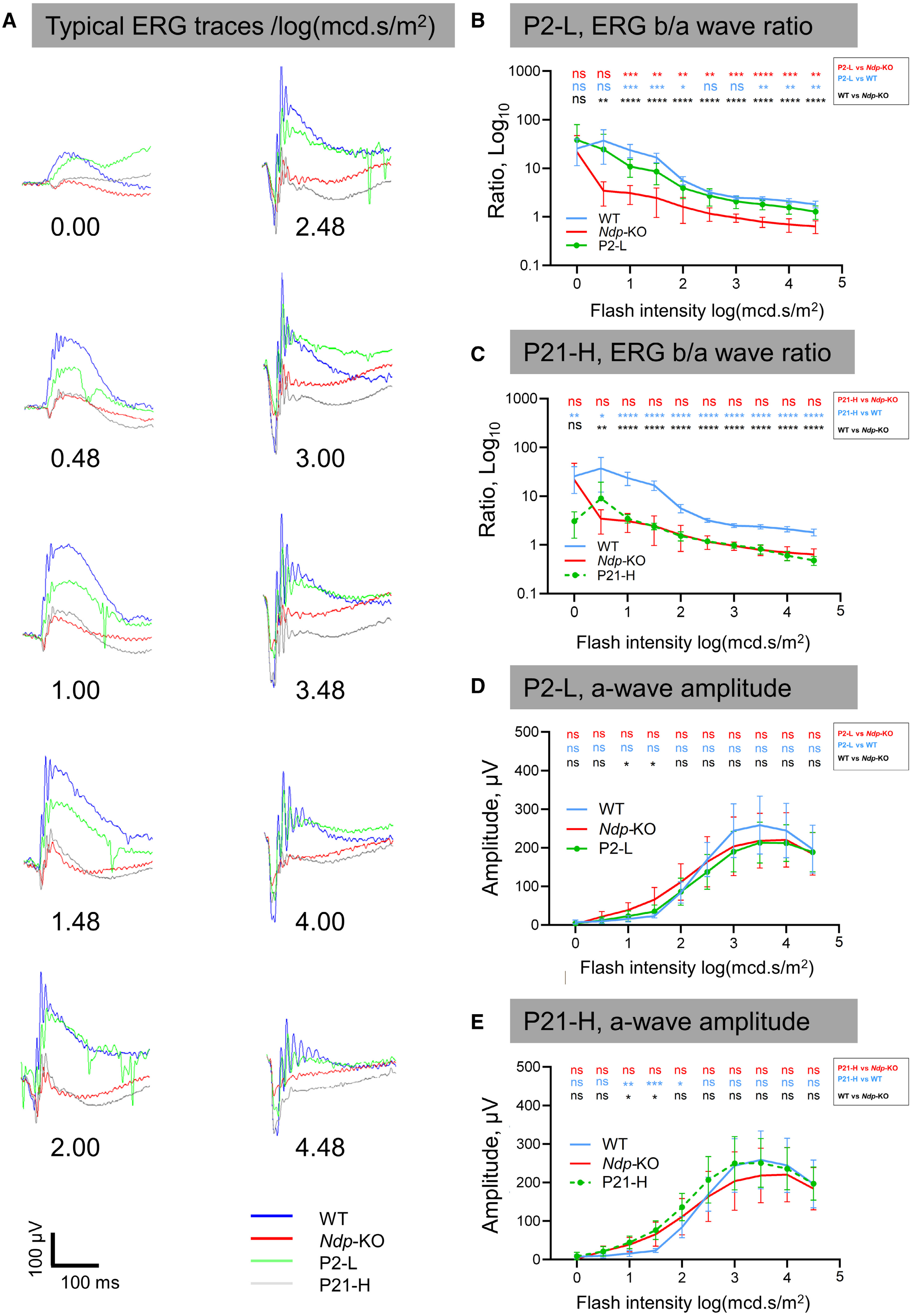
A
Typical ERG traces /log(mcd.s/m2)
0.00
2.48
0.48
3.00
1.00
3.48
1.48
4.00
2.00
4.48
100 µV
100 ms
WT
Ndp-KO
P2-L
P21-H
B
P2-L, ERG b/a wave ratio
Ratio, Log10
Flash intensity log(mcd.s/m2)
P2-L vs Ndp-KO
P2-L vs WT
WT vs Ndp-KO
WT
Ndp-KO
P2-L
C
P21-H, ERG b/a wave ratio
Ratio, Log10
Flash intensity log(mcd.s/m2)
P21-H vs Ndp-KO
P21-H vs WT
WT vs Ndp-KO
WT
Ndp-KO
P21-H
D
P2-L, a-wave amplitude
Amplitude, µV
Flash intensity log(mcd.s/m2)
P2-L vs Ndp-KO
P2-L vs WT
WT vs Ndp-KO
WT
Ndp-KO
P2-L
E
P21-H, a-wave amplitude
Amplitude, µV
Flash intensity log(mcd.s/m2)
P21-H vs Ndp-KO
P21-H vs WT
WT vs Ndp-KO
WT
Ndp-KO
P21-H

Figure EV3.

Figure EV4. Differential gene expression in the cochlea by RNA sequencing and qRT-PCR.

A Venn diagram showing overlap between *Ndp*-KO versus WT DEGs and *Ndp*-KO versus P2-L DEGs.

B Heatmap showing levels of expression of the 18 pathology-related DEGs identified between *Ndp*-KO (red) versus P2-L (blue) in WT, *Ndp*-KO and P2-L treated cochleas (green). Note that these genes showed trends of differential expression between *Ndp*-KO and WT cochleas that did not reach significance. No genes were found to be significantly differentially expressed between WT and P2-L cochleas, indicating rescue by the treatment. In heat map, red indicates upregulated and blue indicates downregulated gene expression in *Ndp*-KO.

C Gene set enrichment analyses of differentially expressed genes between WT and *Ndp*-KO using gene sets were previously defined by transcriptome profiling of FACS sorted CNS vs peripheral endothelial cells (Daneman *et al*, 2010) and previously used to assess transcriptomes of WT and *Ndp*-KO retinas (Zhou *et al*, 2014). Gene sets characterising barrier vasculature, BBB1 and 2, BBB endothelial transporters, CNS endothelial and CNS pericyte were significantly positively correlated (FDR < 0.25) with the WT genotype.

D Dot plot using scRNA seq data of the adult mouse cochlear lateral wall from GEO database: accession numbers GSM5124299, GSM5124300, GSM5124301, and GSM5124302. Gene markers used to distinguish 30 cell type clusters in the UMAP were as previously reported (Gu *et al*, 2020; Bryant *et al*, 2022).

E Dot plot showing expression of the 45 DEGs identified in WT versus *Ndp*-KO analysis in the 30 cell type clusters identified in the adult mouse cochlear lateral wall at the single cell level. Blue arrows indicate endothelial cell genes (*Cldn5*, *Abcb1a* and *Flt1*) downregulated in *Ndp*-KO, and orange arrows indicate upregulated genes in *Ndp*-KO. Box indicates genes significantly different in the P2-L versus *Ndp*-KO comparison. Shaded DEGs are downregulated in the *Ndp*-KO cochlea. Note expression of some DEGs across several different cell type clusters.

 © 2023 The Authors

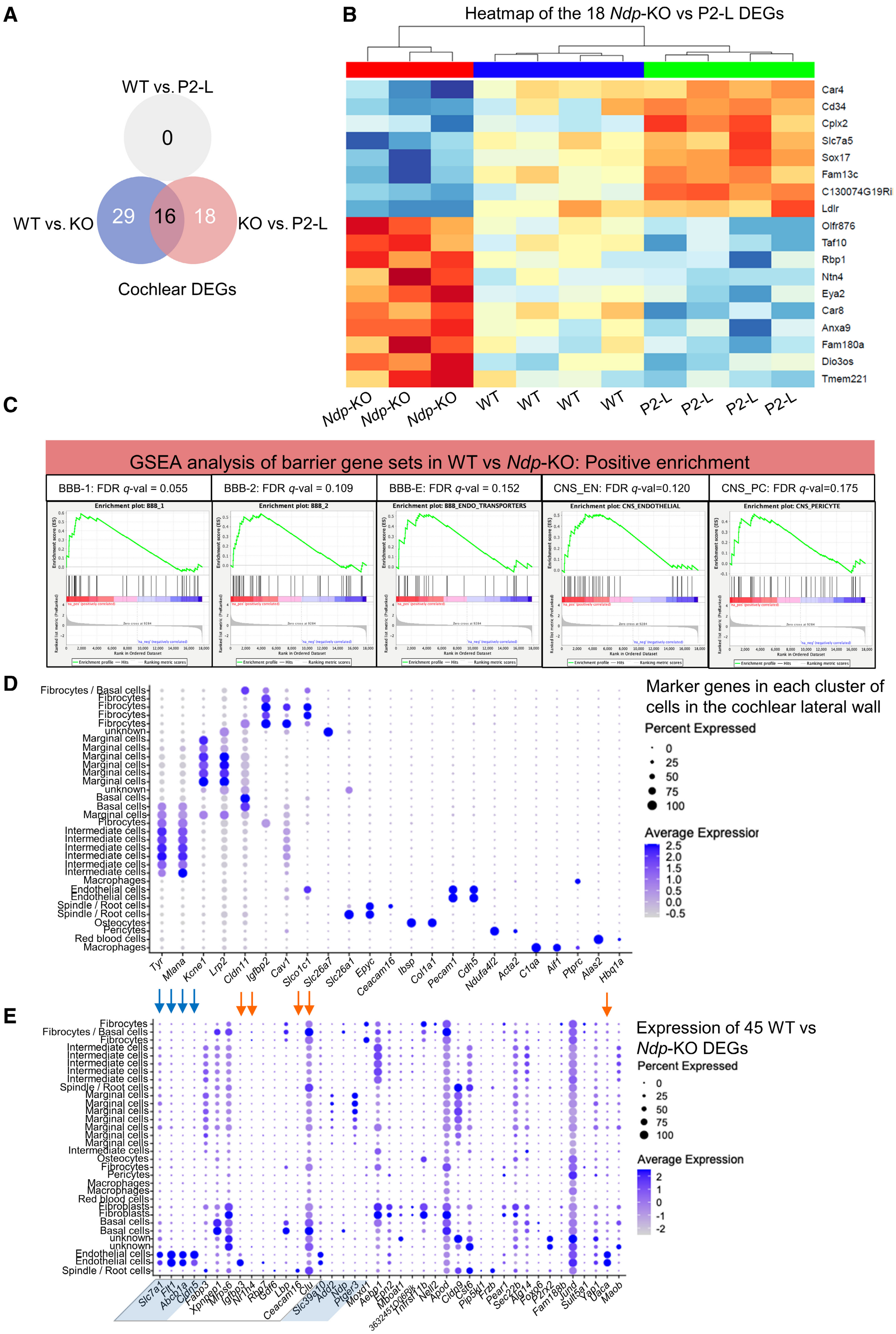
A
WT vs. P2-L
0
WT vs. KO
29
16
18
KO vs. P2-L
Cochlear DEGs
B
Heatmap of the 18 Ndp-KO vs P2-L DEGs
Car4
Cd34
Cplx2
Slc7a5
Sox17
Fam13c
C130074G19Ri
Ldlr
Olfr876
Taf10
Rbp1
Ntn4
Eya2
Car8
Anxa9
Fam180a
Dio3os
Tmem221
Ndp-KO
Ndp-KO
Ndp-KO
WT
WT
WT
WT
P2-L
P2-L
P2-L
P2-L
C
GSEA analysis of barrier gene sets in WT vs Ndp-KO: Positive enrichment
BBB-1: FDR q-val = 0.055
BBB-2: FDR q-val = 0.109
BBB-E: FDR q-val = 0.152
CNS_EN: FDR q-val=0.120
CNS_PC: FDR q-val=0.175
D
Marker genes in each cluster of cells in the cochlear lateral wall
Percent Expressed
0
25
50
75
100
Average Expression
2.5
2.0
1.5
1.0
0.5
0.0
-0.5
Tyr
Mlana
Kcne1
Lrp2
Cldn11
Igfbp2
Cav1
Slco1c1
Slc26a7
Slc26a1
Epyc
Ceacam16
Ibsp
Col1a1
Pecam1
Cdh5
Ndufa4l2
Acta2
C1qa
Aif1
Ptprc
Alas2
Hbq1a
E
Expression of 45 WT vs Ndp-KO DEGs
Percent Expressed
0
25
50
75
100
Average Expression
2
1
0
-1
-2

Figure EV4.

Figure EV5. Statistical analysis of auditory function analysis of DPOAE and ABR thresholds for all groups at 3 months.

A, B DPOAE thresholds of P2-L, P30-H and control groups. *P* values indicate statistically significant difference from WT (blue) and *Ndp*-KO (red). *n* = biological replicates. *n* (WT) = 11, *n* (*Ndp*-KO) = 6, *n* (P2-L) = 9, *n* (P30-H) = 8.

C–E ABR thresholds of P2-L, P-30H and control groups. *P* values (blue) indicate statistically significant difference from WT. *n* = biological replicates. *n* (WT) = 12, *n* (*Ndp*-KO) = 7, *n* (P2-L) = 10, *n* (P21-H) = 8, *n* (P30-H) = 8.

E ABR wave latency for each treatment group compared to *Ndp*-KO and WT. *n* = biological replicates. *n* (WT) = 12, *n* (*Ndp*-KO) = 7, *n* (P2-L) = 10, *n* (P21-H) = 8, *n* (P30-H) = 8.

F ABR wave 1 amplitude for each treatment group compared to *Ndp*-KO and WT. *n* = biological replicates. *n* (WT) = 12, *n* (*Ndp*-KO) = 7, *n* (P2-L) = 10, *n* (P21-H) = 8, *n* (P30-H) = 8.

Data information: Data are shown as mean ± SD. Statistical analysis was performed by two-way repeated measures ANOVA with Tukey's *post hoc* test, comparing each treatment group with WT (blue asterisks) and *Ndp*-KO (red asterisks), and comparing between WT and *Ndp*-KO (black asterisks). *Post hoc* test values: *$P \leq 0.05$, **$P \leq 0.01$, ***$P \leq 0.001$, ****$P \leq 0.0001$.
Source data are available online for this figure.

 © 2023 The Authors

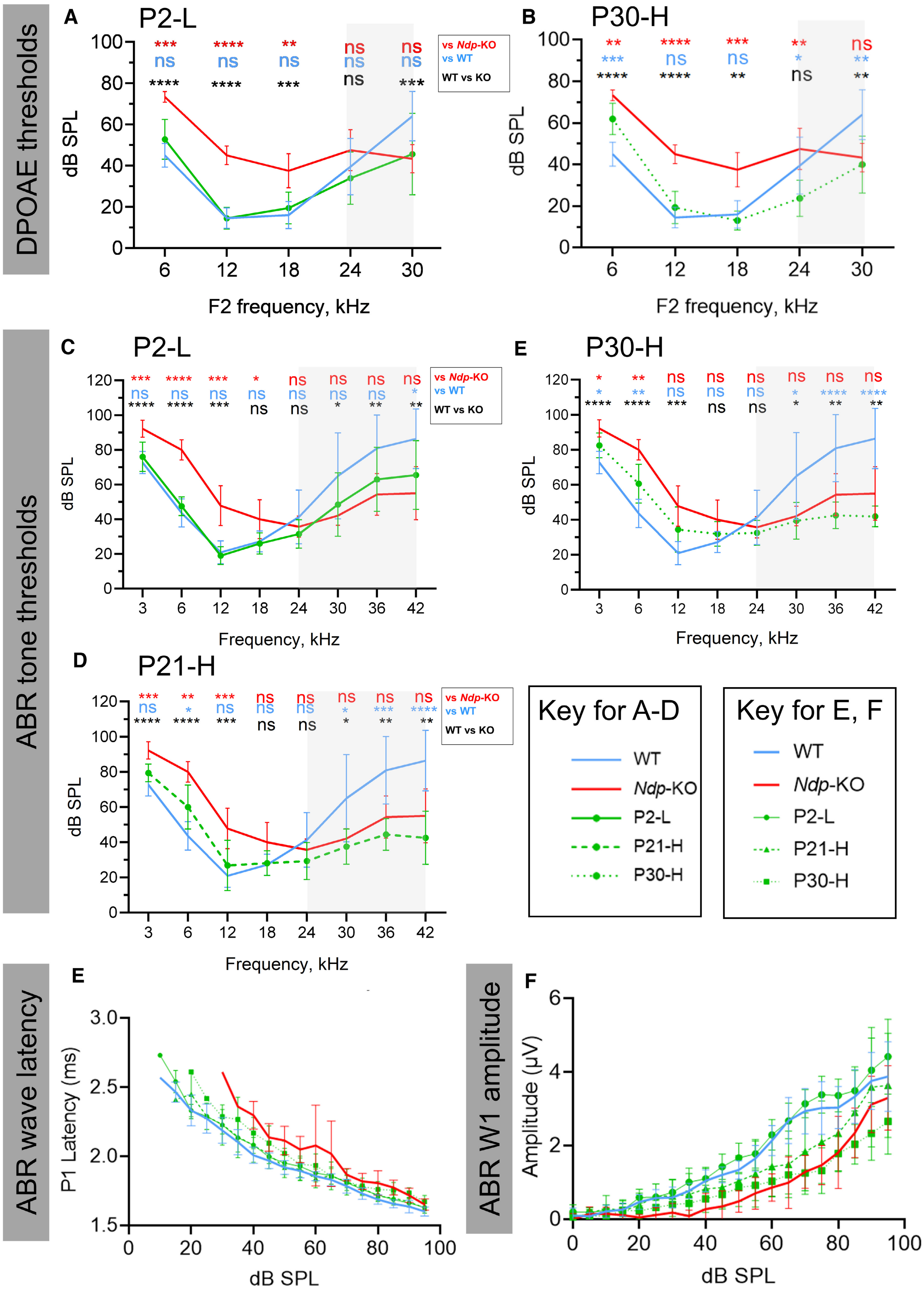
DPOAE thresholds
A P2-L
B P30-H
dB SPL
F2 frequency, kHz
vs Ndp-KO
vs WT
WT vs KO
ABR tone thresholds
C P2-L
E P30-H
D P21-H
Frequency, kHz
Key for A-D
WT
Ndp-KO
P2-L
P21-H
P30-H
Key for E, F
ABR wave latency
E
P1 Latency (ms)
dB SPL
ABR W1 amplitude
F
Amplitude (µV)

Figure EV5.
