# Supplementary material for: Systemic gene therapy rescues retinal dysfunction and hearing loss in a model of Norrie disease
Source: EMBO Mol Med. 2023 Aug 29;15(10):e17393. doi: 10.15252/emmm.202317393 (PMC10565640; doi:10.15252/emmm.202317393)
Supplement: Supplementary file 16 [file EMMM-15-e17393-s016.pdf]

EMBO Molecular Medicine

Systemic gene therapy rescues retinal dysfunction and hearing loss in a model of Norrie disease

Valda Pauzuolyte, Aara Patel, James Wawrzynski, Neil Ingham, Yeh Chwan Leong, Rajvinder Karda, Maria Bitner-Glindzicz, Wolfgang Berger, Simon Waddington, Karen Steel, and Jane Sowden
DOI: 10.15252/emmm.202317393

Corresponding author(s): Jane Sowden (j.sowden@ucl.ac.uk)

Review Timeline:

Submission Date:	5th Jan 23
Editorial Decision:	7th Feb 23
Revision Received:	19th May 23
Editorial Decision:	7th Jun 23
Revision Received:	24th Jul 23
Accepted:	25th Jul 23

Editor: Jingyi Hou / Zeljko Durdevic

Transaction Report:

(Note: With the exception of the correction of typographical or spelling errors that could be a source of ambiguity, letters and reports are not edited. Depending on transfer agreements, referee reports obtained elsewhere may or may not be included in this compilation. Referee reports are anonymous unless the Referee chooses to sign their reports.)

7th Feb 2023

Dear Prof. Sowden,

Thank you again for submitting your work to EMBO Molecular Medicine. We have heard back from three referees who agreed to evaluate your manuscript. As you will see from the reports below, the referees acknowledge the potential interest of the study. However, they raise a series of concerns, which we would ask you to address in a major revision of the manuscript.

I think that the referees' recommendations are relatively straightforward, so there is no need to reiterate their comments. All issues raised by the referees need to be satisfactorily addressed. Please feel free to contact me in case you would like to discuss in further detail any of the issues raised by the reviewers.

We would welcome the submission of a revised version within three months. Please note that EMBO Molecular Medicine strongly supports a single round of revision and that, as acceptance or rejection of the manuscript will depend on another round of review, your responses should be as complete as possible.

EMBO Molecular Medicine has a "scooping protection" policy, whereby similar findings that are published by others during review or revision are not a criterion for rejection. Should you decide to submit a revised version, I do ask that you get in touch after three months if you have not completed it, to update us on the status.

Please also contact us as soon as possible if similar work is published elsewhere. If other work is published we may not be able to extend the revision period beyond three months.

Please read below for important editorial formatting and consult our author's guidelines for proper formatting of your revised article for EMBO Molecular Medicine.

I look forward to receiving your revised manuscript.

Sincerely,
Jingyi

Jingyi Hou
Editor
EMBO Molecular Medicine

*****

When submitting your revised manuscript, please carefully review the instructions that follow below. We perform an initial quality control of all revised manuscripts before re-review; failure to include requested items will delay the evaluation of your revision.

We require:

1) A .docx formatted version of the manuscript text (including legends for main figures, EV figures and tables). Please make sure that the changes are highlighted to be clearly visible.

2) Individual production quality figure files as .eps, .tif, .jpg (one file per figure). For guidance, download the 'Figure Guide PDF': (https://www.embopress.org/page/journal/17574684/authorguide#figureformat).

3) A .docx formatted letter INCLUDING the reviewers' reports and your detailed point-by-point responses to their comments. As part of the EMBO Press transparent editorial process, the point-by-point response is part of the Review Process File (RPF), which will be published alongside your paper.

4) A complete author checklist, which you can download from our author guidelines (https://www.embopress.org/page/journal/17574684/authorguide#submissionofrevisions). Please insert information in the checklist that is also reflected in the manuscript. The completed author checklist will also be part of the RPF.

5) Please note that all corresponding authors are required to supply an ORCID ID for their name upon submission of a revised manuscript.

6) It is mandatory to include a 'Data Availability' section after the Materials and Methods. Before submitting your revision, primary datasets produced in this study need to be deposited in an appropriate public database, and the accession numbers and database listed under 'Data Availability'. Please remember to provide a reviewer password if the datasets are not yet public (see https://www.embopress.org/page/journal/17574684/authorguide#dataavailability).

In case you have no data that requires deposition in a public database, please state so in this section. Note that the Data Availability Section is restricted to new primary data that are part of this study.

7) For data quantification: please specify the name of the statistical test used to generate error bars and P values, the number (n) of independent experiments (specify technical or biological replicates) underlying each data point and the test used to calculate p-values in each figure legend. The figure legends should contain a basic description of n, P and the test applied. Graphs must include a description of the bars and the error bars (s.d., s.e.m.). See also 'Figure Legend' guidelines: https://www.embopress.org/page/journal/17574684/authorguide#figureformat

8) At EMBO Press we ask authors to provide source data for the main manuscript figures. Our source data coordinator will contact you to discuss which figure panels we would need source data for and will also provide you with helpful tips on how to upload and organize the files.

9) Our journal encourages inclusion of *data citations in the reference list* to directly cite datasets that were re-used and obtained from public databases. Data citations in the article text are distinct from normal bibliographical citations and should directly link to the database records from which the data can be accessed. In the main text, data citations are formatted as follows: "Data ref: Smith et al, 2001" or "Data ref: NCBI Sequence Read Archive PRJNA342805, 2017". In the Reference list, data citations must be labeled with "[DATASET]". A data reference must provide the database name, accession number/identifiers and a resolvable link to the landing page from which the data can be accessed at the end of the reference. Further instructions are available at .

10) We replaced Supplementary Information with Expanded View (EV) Figures and Tables that are collapsible/expandable online. A maximum of 5 EV Figures can be typeset. EV Figures should be cited as 'Figure EV1, Figure EV2" etc... in the text and their respective legends should be included in the main text after the legends of regular figures.

- For the figures that you do NOT wish to display as Expanded View figures, they should be bundled together with their legends in a single PDF file called *Appendix*, which should start with a short Table of Content. Appendix figures should be referred to in the main text as: "Appendix Figure S1, Appendix Figure S2" etc.

- Additional Tables/Datasets should be labeled and referred to as Table EV1, Dataset EV1, etc. Legends have to be provided in a separate tab in case of .xls files. Alternatively, the legend can be supplied as a separate text file (README) and zipped together with the Table/Dataset file.

See detailed instructions here:

.

11) The paper explained: EMBO Molecular Medicine articles are accompanied by a summary of the articles to emphasize the major findings in the paper and their medical implications for the non-specialist reader. Please provide a draft summary of your article highlighting

- the medical issue you are addressing,

- the results obtained and

- their clinical impact.

This may be edited to ensure that readers understand the significance and context of the research. Please refer to any of our published articles for an example.

12) For more information: There is space at the end of each article to list relevant web links for further consultation by our readers. Could you identify some relevant ones and provide such information as well? Some examples are patient associations, relevant databases, OMIM/proteins/genes links, author's websites, etc...

13) Author contributions: You will be asked to provide CRediT (Contributor Role Taxonomy) terms in the submission system.

These replace a narrative author contribution section in the manuscript.

14) A Conflict of Interest statement should be provided in the main text.

15) Every published paper now includes a 'Synopsis' to further enhance discoverability. Synopses are displayed on the journal webpage and are freely accessible to all readers. They include a short stand first (maximum of 300 characters, including space) as well as 2-5 one-sentences bullet points that summarizes the paper. Please write the bullet points to summarize the key NEW findings. They should be designed to be complementary to the abstract - i.e. not repeat the same text. We encourage inclusion of key acronyms and quantitative information (maximum of 30 words / bullet point). Please use the passive voice. Please attach these in a separate file or send them by email, we will incorporate them accordingly.

Please also suggest a striking image or visual abstract to illustrate your article as a PNG file 550 px wide x 300-600 px high.

EMBO Molecular Medicine has a "scooping protection" policy, whereby similar findings that are published by others during review or revision are not a criterion for rejection. Should you decide to submit a revised version, I do ask that you get in touch after three months if you have not completed it, to update us on the status.

Please note: When submitting your revision you will be prompted to enter your funding and payment information. This will allow Wiley to send you a quote for the article processing charge (APC) in case of acceptance. This quote takes into account any reduction or fee waivers that you may be eligible for. Authors do not need to pay any fees before their manuscript is accepted and transferred to the publisher.

EMBO Press participates in many Publish and Read agreements that allow authors to publish Open Access with reduced/no publication charges. Check your eligibility: https://authorservices.wiley.com/author-resources/Journal-Authors/open-access/affiliation-policies-payments/index.html

***** Reviewer's comments *****

Referee #1 (Comments on Novelty/Model System for Author):

The study was in general well conceived and carried out. As indicated in my comments, some of the figures were difficult to read and understand. The application of AAV-mediated gene therapy to Norris syndrome is novel and the ability to target both the auditory and visual systems simultaneously is exciting. However, Norrie syndrome is a rare disease and therefore the medical impact is likely not to be high.

Referee #1 (Remarks for Author):

In this study, the authors delivered human Norrie Disease Pseudoglioma (NDP) cDNA by intravenous (IV) injection of AAV9 into a mouse model of Norrie syndrome at three different time points (P2, P21, and P30). They found that neonatal treatment of AAV9-NDP was able to rescue retinal vascularization and cochlear pathology, leading to improved visual and auditory functions, as measured by ERG and ABR, respectively. I think this is a good study. The application of AAV-mediated gene therapy to Norrie syndrome is novel. The use of AAV-mediated gene therapy to target and the auditory and visual systems simultaneously is exciting. My comments are listed below:

1. While the data suggest that AAV9-NDP treatment in neonatal NDP-KO mice improves the auditory and visual functions of these animals, it would be more convincing if NDP expression can be shown in treated mice. Is there an effective antibody for NDP?
2. One important issue to address with this study is the fact that AAV9-NDP was delivered intravenously. It would be helpful to examine whether the transgene is expressed in other organ systems in order to assess for the possibility of systemic toxicity.
3. In Figure 2, it is not clear to me how long after AAV9-NDP treatment were the images obtained. It would be helpful to indicate this in the figure/figure legend.
4. In Figure 3G-3J, the authors reported that AAV9-NDP treatment improved retinal vascularization. However, it is difficult for me to tell this from the images shown. How long after gene therapy treatment were these images taken?
5. In Figure 3K-3N, the authors reported that AAV9-NDP treatment improved CLDN5 expression and improved tight junction organization. However, it is difficult to see the differences between treated and untreated mice on the images shown. How long after gene therapy treatment were these images taken?
6. In Figure 3Q and 3R, it is not clear to me what the black asterisks represent. This is a common issue throughout the manuscript (also see Fig. S5 and Fig. S8).
7. In Figure 5, the authors quantified capillary branching point numbers in different regions of stria vascularis. It is not clear to me what the different regions (1-4) indicate. It would be helpful to have a cartoon to help the readers. In addition, Figure 5B (which is not labeled) seems to indicate the regions as 1/8 to 4/8. Were there 8 regions that were examined? I would suggest the authors to clarify.
8. In Figure 5G-5J, it is not clear to me what the red arrows and orange arrows are pointing to. The figure legend indicates that the red arrows are for "abnormal meshwork vessels", and the orange arrows are for "barrier vessels". It is not clear how these

vessels are defined.
9. In Figure 5B, the only region where there is a difference in branching points between the treated and non-treated mice is in region 2/8. Does this specific region have any special physiologic properties?
10. In Figure 7B and 7D, the authors showed that NDP-KO mice treated with AAV9-NDP showed improvement in DPAOE and ABR. Were these differences statistically significant?

Referee #2 (Remarks for Author):

This is a well-written and important study to investigate whether or not systemic delivery of an AAV9-mediated NDP gene at different stages of development (P2, P21, and P30) could prevent and rescue hearing and vision in Norrie disease. This therapeutic study follows a previous report from the lab published in JCI insight on cochlear pathogenesis in Norrie disease. The finding would benefit clinical treatment of Norrie disease.

However, some of the data need to be consolidated

Results:

• No data demonstrate the efficacy of AAV9.NDP expression in vivo. Figure 2 lists animal weight and shows pattern of AAV9-GFP distribution in the retina and cochlea. It is essential to assess the efficacy of NDP gene expression in the tissue. This can be done with WB (protein level) in the transfected retina and cochlea.
• The lower effectiveness on vascular development in the later stages might be due to viral transfection to the cochlea failing, as shown in Figure 2 (E-I). The AAV9-GFP signal is unexpectedly weak. The BLB should be mature at P21 and P30 in the WT but not in the NDP mutant. If the tissues demonstrated in Figure 2 were collected from the NDP mutant, we would expect to see more GFP signal as a vascular abnormality and loss of BLB integrity (increased vascular leakage) in the mutant, especially with IV delivery. This needs to be explained.
• Additional data to demonstrate CLDN5 expression in the stria vascularis is necessary. It is not sufficient to only show expression in the SL. The endothelium in the SL does not form as tight and controls a barrier as the strial endothelium. NDP KO is known to have less effect on the development of SL but a significant effect on SV development (Heidi L. Rehm et al., 2002).
• Figure 5 demonstrates increased vascular volume after gene therapy. It would be better if markers, such as EdU, were used to show growth of new blood vessels with NDP gene therapy.
• Results presented in S4 are confusing. When delivered at P30, HCs are apparently transfected. However, HCs are not transfected when delivered at P2 and P21? This result is intriguing but needs further discussion. Again, are the samples from the NDP mutant or WT? this needs to be clarified in the figure caption.
Discussion:
• NDP mutation causes abnormal development and morphology in retinal and cochlear blood vessels. However, in their Discussion, the authors' state "Lack of NDP does not result in major morphological abnormalities or absence of the cochlear vasculature but only disrupts the blood-labyrinth barrier". This statement is not very accurate. Heidi L. Rehm et al., 2002 describe NDP mutation pathology in the cochlea. They descript significantly enlarged and underdeveloped vessels in the stria vascularis (vessels in the SL are not as affected, as NDP protein is predominantly expressed in the stria and other parts of cochlea, less so in the SL). Accurate references need to be cited here.
• The authors could improve the paper with discussion of the relationship between NDP gene mutation and up-regulation of genes for Plvap, Clu, Ceacam16, Nr1h4 Ndp-KO; Abcb1a, and Cldn5, and down-regulation of genes for Slc7a1 and Slc7a5 that lead to retinal and cochlear pathology.
• Most of the vectors found recently trigger an innate immune response. Is there immune response to AAV9-gene vectors? It would be nice to discuss this in the Discussion regarding the safety.
Minor:

"CNS pericyte)". Delete ")".

Referee #3 (Comments on Novelty/Model System for Author):

See my comments to authors

Referee #3 (Remarks for Author):

Comments

Norrie disease is a devasting disease that affects vision and hearing in patients without treatment. The study aims to develop a gene therapy by AAV as a treatment for Norrie disease using a mouse model and lay the groundwork for future application in patients. Overall the study was well designed and executed, with encouraging data that strongly support continuing the effort

towards the clinic.

There are some issues in the study which should be addressed.

1. The study tested interventions at different time points to rescue vision and hearing. Not surprisingly, early intervention yielded a better rescue effect. While P2 injection is before the onset of the disease, it is important to know the other time points in relation to hearing and vision loss. Please provide a figure to show the onset and progression of the phenotypes and discuss the rescue effect according to the onset of the disease. This part will help to understand when the intervention will likely work and to what degree. For instance, the injections at P21 and P30 recovered some hearing that is less than the P2 injection. Do we know if hearing loss has started at P21 or not? The discussion should include the part about preventing and reversing hearing loss (it does not apply to blindness as it occurs very early). Most gene therapies for hearing loss work by preventing/slowing down hearing loss and not by reversing hearing loss already occurred. This information is essential in the design of any clinical trial.

2. The significant conclusions should include 1). Systemic delivery at early stage results in the rescue of vision and hearing; 2). The time point used in mice may not be applicable to humans due to the difference in the development of ears and eyes and the onset of blindness and hearing loss; 3). Local delivery may be advantageous for humans. The study did not characterize other organs/tissues that AAV9 targets. The expression of NDP in unrelated tissues may cause safety concerns over time. Beyond the safety feature, local delivery allows testing a range of virus doses.

3. One major limitation of the study is a relatively short time window post injection. In most gene therapy studies, the results tend to be more effective at the early stage, but the effects diminish over time. As NDP manifests as late onset progressive hearing loss, the long term outcome will be important to evaluate how sustained the treatment is. Please discuss the limitation of the current study.

4. It will be helpful to present a figure to show the expression of Ndp in the retina and cochlea, so readers can compare it with the AAV-mediated Ndp expression. I am not clear if the cells transduced by the AAV are the same Ndp expressing cells or there are some differences. This information will help understand the rescue effect.

5. The RNAseq study is informative as it shows the rescue effect on the molecular level by restoring gene expression deficient in the Ndp model. The confirmation by RT-PCR correlates well with the rescue, i.e. P2-L injection restored downstream genes more robustly, which leads to better functional recovery. Please discuss the point.

6. P9, 2nd paragraph, the description is not accurate. Judging by RT-PCR, P2 injection better recapitulated the expression level of genes (Fig.4E, F, G, H, I). This data is informative as it may predicate the final outcome of the treatment, which is the best by P2 intervention. Please re-write the paragraph as it leaves people with the impression that later interventions work just well as early intervention.

7. P4, "with anti-FLAG immunostaining on the cell surface (yellow, Fig. 1 B-B')", the color should be red, not yellow.

8. I don't see much labeling of GFP in the SGNs in the P30-H group. As the result, the statement" Spiral ganglion neurons were transduced in all treatment groups (Fig. 2E, Fig. S4 A-E)" should be rewritten.

9. Judging by GFP labeling, transduction at p2-L and p21-H seems to be efficient in targeting the lateral wall, but not at p30, even at a high dose.

10. Fig.1E, explain the arrows. Are they SGNs? Should do double labeling with TuJ1 to show GFP+ cells are neurons. Looks like other cells, in addition to SGNs, are also GFP+.

11. In later interventions, the number of cells transduced is fewer, and the expression of the transgene is lower. This could be due to insufficient delivery to the target cells in mature animals. It will be important to perform a comparative study in the future by local delivery, compare the result with the current study, and decide a possible route for human study.

Jingyi Hou
Editor
EMBO Molecular Medicine

Dear Editor,

Re: Systemic gene therapy rescues retinal pathology and hearing loss in a model of Norrie disease

Authors: Valda Pauzuolyte, Aara Patel, James R. Wawrzynski, Neil J. Ingham, Yeh Chwan Leong, Maria Bitner-Glindzicz, Wolfgang Berger, Simon Waddington, Karen P. Steel, Jane C. Sowden.

Thank you for the favourable reviews and positive interest in our study, and the opportunity to submit a revised manuscript.

We thank the Reviewers' for their helpful comments. We have addressed all issues raised and hope that the manuscript is now considered to be satisfactory for publication.

We have provided all the source data for the main manuscript figures.

As we describe, the application of AAV-mediated gene therapy to Norris syndrome is novel and the ability to target both the auditory and visual systems simultaneously is of significant interest. To our knowledge this is the first study using AAV9 to treat a progressive hearing loss disorder and the efficacy of the therapy after the onset of degenerative disease shows that it has potential for clinical translation.

Please find below the Reviewers' reports in blue font and our detailed point-by-point responses to their comments in black font. Revised test inserted into the manuscript is marked in red font.

Yours sincerely,

Jane Sowden

Referee #1 (Comments on Novelty/Model System for Author):
The study was in general well conceived and carried out. As indicated in my comments, some of the figures were difficult to read and understand. The application of AAV-mediated gene therapy to Norris syndrome is novel and the ability to target both the auditory and visual systems simultaneously is exciting. However, Norrie syndrome is a rare disease and therefore the medical impact is likely not to be high.

Referee #1 (Remarks for Author):
In this study, the authors delivered human Norrie Disease Pseudoglioma (NDP) cDNA by intravenous (IV) injection of AAV9 into a mouse model of Norrie syndrome at three different time points (P2, P21, and P30). They found that neonatal treatment of AAV9-NDP was able to rescue retinal vascularization and cochlear pathology, leading to improved visual and auditory functions, as measured by ERG and ABR, respectively. I think this is a good study. The application of AAV-mediated gene therapy to Norrie syndrome is novel. The use of AAV-mediated gene therapy to target and the auditory and visual systems simultaneously is exciting.
My comments are listed below:
1. While the data suggest that AAV9-NDP treatment in neonatal NDP-KO mice improves the auditory and visual functions of these animals, it would be more convincing if NDP expression can be shown in treated mice. Is there an effective antibody for NDP?

We appreciate the point made by the Reviewer. We employed an EGFP-P2A-NDP construct to assess AAV9.NDP transduction as previously we and others were not able to detect NDP protein by or Western blotting analysis of WT mouse tissue when testing a number of commercially available antibodies; detection by immunostaining is also complicated by the fact that NDP is secreted and NDP binding to extracellular matrix makes it difficult to extract from the tissue. We therefore used EGFP for both labelling the transduced cells and for relative estimation of NDP levels, as both NDP and EGFP proteins are expected to be translated at a constant stoichiometric ratio from the common EGFP-P2A-NDP mRNA as separate proteins due to the P2A linker. We have reiterated this point in the manuscript.

We have nevertheless succeeded in overcoming these technical challenges to address the Reviewer's comment.

We now provide a new Figure 3, showing NDP immunostaining in retinal cryosections at 2 months and in cochlea cryosections at 3 months after treatment at P2 and P21 in (Fig 3 L-P) and described in a new section "Transgenic norrin was detected by immunostaining in the retina and cochlea of treated mice" on pages 7-8. We have added new Fig EV2 and Fig EV3 including high magnification insets, showing the separate anti NDP and EGFP fluorescent channels.

In the new Figure 3, we have provided Western blot analysis of EGFP in retina and cochlea from treated *Ndp*-KO mice. Note that EGFP levels in Western blot are consistent with those seen in tissue whole mounts and sections in both retina and cochlea.

We also were able to detect NDP protein by Western blot analysis of *Ndp*-KO retina at 2 months after AAV9.NDP treatment at P21. Note that this treatment group (P21-H) also showed the highest levels of construct mRNA as assessed by additional qRT-PCR analysis. NDP protein was not detected in other samples by Western blot, which we think may be a result of its lower levels and the difficult isolation of NDP from the tissue due to its property to attach to extracellular matrix (Perez-Vilar, J. and R.L. Hill, 1997). This is described on page 7 and Fig 3.

Together these data confirm NDP expression in treated mice and show that we are delivering human NDP to the retina and the cochlea.

2. One important issue to address with this study is the fact that AAV9-NDP was delivered intravenously. It would be helpful to examine whether the transgene is expressed in other organ systems in order to assess for the possibility of systemic toxicity.

AAV9 is known to transduce multiple sites of the organism, when injected intravenously (Massaro, Hughes et al., 2020, Mattar, Wong et al., 2015). We did not observe any abnormal behaviour or weight abnormalities in the treated mice during this study suggesting good toleration of the treatment (Fig 2A; Fig S2G, H). Only cochlear and retinal tissues- the sites of known Norrie disease pathology - were analysed in this study, and future studies will be needed to assess other organs and the possibility of long-term toxicity.

3. In Figure 2, it is not clear to me how long after AAV9-NDP treatment were the images obtained. It would be helpful to indicate this in the figure/figure legend.

We apologise that this was not clear. The schematic in Figure 1F shows the study design. Images shown in Figure 2 are from samples collected at 2 months (B-I). We have indicated this in the Figure legend.

4. In Figure 3G-3J, the authors reported that AAV9-NDP treatment improved retinal vascularization. However, it is difficult for me to tell this from the images shown. How long after gene therapy treatment were these images taken?

The images in Fig 4 (previously Fig 3) are at 3 months (C-F) and 2 months (G-N). We have indicated this is the Figure legend. We improved the brightness of the high magnification images and added arrows, pointing to the rescued vessels. To further highlight the improved vascularisation, we have also now included a new figure (Fig EV4) which provides lower magnification views of transverse cross sections to demonstrate the degree of rescue of the retinal vasculature across the retina (Fig EV4 A-D).

5. In Figure 3K-3N, the authors reported that AAV9-NDP treatment improved CLDN5 expression and improved tight junction organization. However, it is difficult to see the differences between treated and untreated mice on the images shown. How long after gene therapy treatment were these images taken?

The images in Fig 4K-N (previously Fig 3K-N) are at 2 months. This was now indicated in the Figure legend. We increased the brightness of the original images and added arrows, pointing to the rescued vasculature. We have also now provided additional high magnification images of transverse retinal cross sections (Fig EV4 E-H), which show triple co-staining and colocalization of IB4 (vasculature marker) with CLDN5 and PLVAP at 2 months.

To further demonstrate the degree of rescue of the retinal vasculature we also now provide new qRT-PCT analysis of *Plvap* and *Cldn5* gene expression in Fig 4O-P. These data demonstrate restoration (P2-L) or

improvement (P21-H, P30-H) of *Cldn5* and *Plvap* mRNA levels in *Ndp*-KO samples collected at 2 months, which is consistent with the observations in the immunostained tissue.

6. In Figure 3Q and 3R, it is not clear to me what the black asterisks represent. This is a common issue throughout the manuscript (also see Fig. S5 and Fig. S8).

We apologise for lack of clarity. Three colours were used to represent significance values of the *post hoc* comparisons between different groups. Note that there are more groups in two-way ANOVA analyses, which requires three types of labelling. The black asterisks represent the *post hoc* comparison between WT and *Ndp*-KO, blue asterisks represent the comparison between treatment groups and WT mice, and red represent the comparison between treatment groups and *Ndp*-KO mice in all repeated-measures two-way ANOVA analyses. Similarly, in one-way ANOVA analysis, blue asterisks represent comparison with the WT, red asterisks represent comparison with the *Ndp*-KO mice. A colour key has now been added to all figures with graphs. By this analysis we report restoration to WT levels and significant difference from the *Ndp*-KO pathological state.

7. In Figure 5, the authors quantified capillary branching point numbers in different regions of stria vascularis. It is not clear to me what the different regions (1-4) indicate. It would be helpful to have a cartoon to help the readers. In addition, Figure 5B (which is not labelled) seems to indicate the regions as 1/8 to 4/8. Were there 8 regions that were examined? I would suggest the authors to clarify.

Images of the lateral wall were divided into 8 equal regions from apex to base for image analysis and quantification. We have updated all figure annotation so that these are referred in apex-to-base direction to as 1/8, 2/8, 3/8, 4/8, 5/8, 6/8, 7/8 and 8/8 consistently. For clarity, stereoscopic images of a dissected cochlea with the modiolus/organ of Corti and the lateral wall separated have now been added in Fig S4. Also note examples of a full-length mapped lateral wall and dissected and mapped organs of Corti in Fig S4 A-E.

8. In Figure 5G-5J, it is not clear to me what the red arrows and orange arrows are pointing to. The figure legend indicates that the red arrows are for "abnormal meshwork vessels", and the orange arrows are for "barrier vessels". It is not clear how these vessels are defined.

We described previously the atypical staining pattern of Claudin-5 on *Ndp*-KO vessels in the spiral ligament and stria vascularis (Bryant, Pauzuolyte, et al, 2022, JCI Insight). We apologise that this was not clearly described. We have simplified the description and have included higher magnification images and added arrows in Fig 6 to point out the atypical low CLDN5 staining pattern observed on some, but not all vessels, in the *Ndp*-KO. We observed that only the early (P2-L) but not the late treatment prevented the vessel pathology.

9. In Figure 5B, the only region where there is a difference in branching points between the treated and non-treated mice is in region 2/8. Does this specific region have any special physiologic properties?

We are not aware of any specific physiologic properties of the apical vasculature.

We previously demonstrated that the morphological abnormalities of the stria vascularis in the C57BL/6 *Ndp*-KO mouse were most prominent in the apical region (Bryant, Pauzuolyte et al., 2022). Development of the stria vascularis capillary network is also known to progress from the base towards the apex (Iwagaki, Suzuki et al., 2000).

10. In Figure 7B and 7D, the authors showed that NDP-KO mice treated with AAV9-NDP showed improvement in DPAOE and ABR. Were these differences statistically significant?

Yes, these differences were statistically significant and are now detailed in the text on Page 14 and 15 in red font, also we refer the reader to the full statistical analysis provided in the extended view Fig EV7 (indicated in red font; Fig 7B, D are now Fig 8B, Fig 7D). Statistical tests used are also highlighted in red in the legend to Fig EV7 on page 37.

We have depicted all the *post hoc* comparisons between the different treatment as separate graphs in the extended view together with the statistical analyses (indicated by asterisks; Fig EV7A-D). Note that

throughout the study significance values of comparisons between WT and *Ndp*-KO are depicted as black asterisks, comparisons between WT and treatment groups as blue asterisks, and comparisons between *Ndp*-KO and treatment groups as red asterisks. This is described in the text and also in the Fig 7 legend (red font, page 14 and 15).

Referee #2 (Remarks for Author):
This is a well-written and important study to investigate whether or not systemic delivery of an AAV9-mediated NDP gene at different stages of development (P2, P21, and P30) could prevent and rescue hearing and vision in Norrie disease. This therapeutic study follows a previous report from the lab published in JCI insight on cochlear pathogenesis in Norrie disease. The finding would benefit clinical treatment of Norrie disease.
However, some of the data need to be consolidated
Results:
• No data demonstrate the efficacy of AAV9.NDP expression in vivo. Figure 2 lists animal weight and shows pattern of AAV9-GFP distribution in the retina and cochlea. It is essential to assess the efficacy of NDP gene expression in the tissue. This can be done with WB (protein level) in the transfected retina and cochlea.

We agree with the point the Reviewer makes regarding the importance of assessing the *NDP* gene expression in tissue of the treated mice. Please also see reply to Reviewer 1, point 1 above.

Because of the lack of NDP antibodies that reliably detect NDP we designed and used an EGFP-P2A-NDP tagged vector throughout this study (referred to as AAV9.NDP for simplicity). We have showed the pattern of EGFP transduction in whole mounts and sections of tissue by immunostaining for EGFP.

As described above we have now provided additional data using additional methods to quantify NDP transgene gene expression in the transduced cochlea and retina and the resultant level of GFP protein in these target tissues.

(i) In Fig 4 (now Fig 5) we showed qRT-PCR analysis of the EGFP-P2A-NDP mRNA levels. This has now been moved new Figure 3. We have added additional qRT PCR analysis performed using primers complementary to conserved coding sequences in mouse *Ndp* and human *NDP* (*Ndp/NDP* primers, Fig 3 A, B, F). This allowed the comparison of human NDP transgene expression levels with endogenous *Ndp* mRNA expression. We show expression of the transgenic NDP mRNA was higher than the mouse intrinsic *Ndp* mRNA in treatment groups P2-L, P21-H, P30-H. "Transduction levels at 2 months appeared highest in the cochlea after treatment at P2, and highest in the retina after treatment at P21." This point has been added to the text on page 7.
(ii) In new Fig 3 data we have also included Western blots comparing EGFP levels between the different treatment groups in protein preparations from cochlea and retina. Available antibodies do not readily detect the intrinsic mouse norrin protein, and we only identified one antibody, which detected recombinant NDP (seven commercially available antibodies were tested). "Western blot assays detected EGFP protein levels consistent with qRT-PCR and immunohistochemistry results". This point has been added to the text on page 7.
(iii) We also performed immunostainings of retina and cochlea tissue transverse cryosections to confirm the presence of the recombinant NDP protein in the treated mice. The anti-NDP immunostaining successfully confirmed presence of the NDP protein in both the retina and the cochlea, and the results are now included as Fig 3 and Fig EV2 and Fig EV3 and described on page 7-8.

• The lower effectiveness on vascular development in the later stages might be due to viral transfection to the cochlea failing, as shown in Figure 2 (E-I). The AAV9-GFP signal is unexpectedly weak. The BLB should be mature at P21 and P30 in the WT but not in the NDP mutant. If the tissues demonstrated in Figure 2 were collected from the NDP mutant, we would expect to see more GFP signal as a vascular abnormality and loss of BLB integrity (increased vascular leakage) in the mutant, especially with IV delivery. This needs to be explained.

We appreciate the Reviewers comment and confirm that our analyses show that with the same dose of virus particles per kilogram body weight we achieve a relatively low transduction in the cochlea after AAV9.NDP delivery at later timepoints (P21, P30). We confirm that the cochlear tissues shown in Fig 2 (E-I)

show treated *Ndp*-KO mice. We agree that the different efficacies achieved by treating at different treatment timepoints is an important and clinically relevant point. However, note that transduction is not failing as both P21-H and P30-H groups have higher levels of the transgenic human *NDP* mRNA than the endogenous levels of *Ndp* in the WT mice (see the new Fig 3 and the new qRT-PCR analyses F-H).

We do not have an explanation for the lower transduction efficiency in adult compared to neonates but it likely relates to the differences in the cochlear tissue composition and expression of the cell surface receptors required for the AAV attachment and entry into the cells in neonate and adult mice. We have not treated wildtype mice so cannot comment on the relative transduction efficiencies in WT versus *Ndp*-KO.

• Additional data to demonstrate CLDN5 expression in the stria vascularis is necessary. It is not sufficient to only show expression in the SL. The endothelium in the SL does not form as tight and controls a barrier as the strial endothelium. NDP KO is known to have less effect on the development of SL but a significant effect on SV development (Heidi L. Rehm et al., 2002).

We have now added new data to Fig 6. showing high magnification images of CLDN5 immunostaining in the stria vascularis and the spiral ligament (Fig 6K-R) and revised the text on page 12-13 indicated in red "In the P2-L, but not the P30-H, treatment groups, claudin-5 was restored (Fig 6M-R) and was comparable with the WT distribution. In the spiral ligament capillary and stria vascularis microvasculature networks, treatment of neonates, but not older mice ameliorated the pathology".

In our recent study (Bryant, Pauzuolyte et al., 2022) we did already demonstrate that there is an early pathology in the spiral ligament in addition to abnormality of the stria vascularis.

• Figure 5 demonstrates increased vascular volume after gene therapy. It would be better if markers, such as EdU, were used to show growth of new blood vessels with NDP gene therapy.

We thank the reviewer for this interesting suggestion, which could be further employed in the future to study the mechanisms of the cochlear vascular pathology development. However, in this case, we do not show evidence of growth of new vessels or increased vascular volume after gene therapy, rather that early treatment prevents abnormal morphology of cochlear vessels, which could be formed in ways other than lack of vascular outgrowth.

• Results presented in S4 are confusing. When delivered at P30, HCs are apparently transfected. However, HCs are not transfected when delivered at P2 and P21? This result is intriguing but needs further discussion. Again, are the samples from the NDP mutant or WT? this needs to be clarified in the figure caption.

We apologise for insufficient clarity of the Fig EV1 (previously S4). Across the study, *Ndp*-KO animals were treated, not the WTs. The hair cells (IHC and OHC) were rarely transduced in all treatment groups. We have now included an example of an organ of Corti whole mount sample from the P30-H treatment group counterstained with an anti-MyoVIIA antibody to clarify the location of the IHC and OHC (Fig EV1F). Arrows indicating the location of the IHC/OHC have also been added.

Discussion:

• NDP mutation causes abnormal development and morphology in retinal and cochlear blood vessels. However, in their Discussion, the authors' state "Lack of NDP does not result in major morphological abnormalities or absence of the cochlear vasculature but only disrupts the blood-labyrinth barrier". This statement is not very accurate. Heidi L. Rehm et al., 2002 describe NDP mutation pathology in the cochlea. They descript significantly enlarged and underdeveloped vessels in the stria vascularis (vessels in the SL are not as affected, as NDP protein is predominantly expressed in the stria and other parts of cochlea, less so in the SL). Accurate references need to be cited here.

We have corrected this statement on page 16 (indicated in red) and cited the study by (Rehm, Zhang et al., 2002) as well as our previous study, which demonstrate abnormalities in both stria and ligament capillaries (Bryant, Pauzuolyte et al., 2022).

"Lack of NDP in the mouse cochlea causes morphological vessel abnormalities and disrupts the cochlear vascular barrier (Bryant et al., 2022, Rehm et al., 2002)."

Please note that Rehm et al, 2002, use *Ndp*-KO mice on a different strain background, which may also account for differences from our C57BL/6 *Ndp*-KO mice.

• The authors could improve the paper with discussion of the relationship between NDP gene mutation and up-regulation of genes for Plvap, Clu, Ceacam16, Nr1h4 Ndp-KO; Abcb1a, and Cldn5, and down-regulation of genes for Slc7a1 and Slc7a5 that lead to retinal and cochlear pathology.

We have discussed the DEG findings in the results on page 11. We have added the additional point that Ndp signalling may regulate common pathways important for vascular endothelial cell in the retina and the cochlea and as indicated in red below:

"Endothelial cell DEGs associated with the normal function of cochlear microvasculature were identified as likely downstream targets of NDP signalling. Barrier gene *Cldn5*, vascular endothelial growth factor receptor 1 gene (*Flt1*), which is important for vascular barrier and branching (Eilken, Diéguez-Hurtado et al., 2017, Wang, Zhang et al., 2019, Zhang, Hou et al., 2021), and molecule transporter genes, *Abcb1a, Slc7a1*, were all downregulated in the *Ndp*-KO and returned to normal levels with treatment (Fig 4 A). *Abcb1a* is associated with hearing loss and increased sensitivity to ototoxicity in mice (Saito, Zhang et al., 2001, Zhang, Saito et al., 2000). *Slc7a1* is an amino acid transporter, typical to normal blood brain barrier (Yahyaoui & Pérez-Frías, 2019). *Slc7a5,* another amino acid transporter gene known to be expressed in cochlear vasculature (Sharlin, Visser et al., 2011) showed increased expression after treatment (Fig E6 B). Investigation of a scRNAseq atlas data set of the mouse cochlea confirmed that these genes are expressed in vascular endothelial cells of the cochlear lateral wall (Fig EV6D). These findings of down regulation of endothelial cell barrier markers and transporters in the *Ndp*-KO, were consistent with microvasculature as a primary site of pathology in Norrie disease supporting the hypothesis that microvascular disruption leads to an unsuitable microenvironment for hair cell survival in the Norrie cochlea. The genes upregulated in the *Ndp*-KO are also expressed in the lateral wall (Fig EV6D, E) and considering their function could be related to the Norrie disease cochlear pathology (Fig 4 A; Fig EV6). *Clu* is expressed in multiple cell types in the cochlea and encodes a secreted chaperone protein (Lee, Shin et al., 2017) known to be involved in responses to cell and tissue damage (Rohne, Prochnow et al., 2016). Ceacam16 is a secreted glycoprotein that interacts with the acellular tectorial membrane and is critical for maintaining this structure (Zheng, Miller et al., 2011). It is also expressed in spindle/root cells of the lateral wall (Fig E6 E). Nr1h4 is thought to play a role in vascular endothelial homeostasis (He, Li et al., 2006)". Of note is the fact that several of the cochlea DEGs have also been identified in studies of differential gene expression in the Ndp-KO retina. For example, *Cldn5* and *Slc7a1* were identified as downregulated in the postnatal *Ndp*-KO retina (Schafer, Luhmann et al., 2009, Zhou, Wang et al., 2014) suggesting NDP signalling acts on similar pathways needed for vascular endothelial cell function in the cochlea and the retina."

• Most of the vectors found recently trigger an innate immune response. Is there immune response to AAV9-gene vectors? It would be nice to discuss this in the Discussion regarding the safety.

We have added the following additional point to the discussion on page 17.

"Immune responses to AAV9 and genotoxicity have been previously reported with systemic administration at high doses in some animal model studies (Kuzmin, Shutova et al., 2021); for example, associated with ataxia and acute liver toxicity in non-human primates and piglets at doses of $2{\times}10^{14}$ vg/kg (Flotte & Buning, 2018, Hinderer, Katz et al., 2018)"

Minor:
"CNS pericyte)". Delete ")".

This change has been made.

Referee #3 (Remarks for Author):
Comments
Norrie disease is a devasting disease that affects vision and hearing in patients without treatment. The study aims to develop a gene therapy by AAV as a treatment for Norrie disease using a mouse model and lay the groundwork for future application in patients. Overall the study was well designed and executed, with encouraging data that strongly support continuing the effort towards the clinic.
There are some issues in the study which should be addressed.

1. The study tested interventions at different time points to rescue vision and hearing. Not surprisingly, early intervention yielded a better rescue effect. While P2 injection is before the onset of the disease, it is

important to know the other time points in relation to hearing and vision loss. Please provide a figure to show the onset and progression of the phenotypes and discuss the rescue effect according to the onset of the disease. This part will help to understand when the intervention will likely work and to what degree. For instance, the injections at P21 and P30 recovered some hearing that is less than the P2 injection. Do we know if hearing loss has started at P21 or not? The discussion should include the part about preventing and reversing hearing loss (it does not apply to blindness as it occurs very early). Most gene therapies for hearing loss work by preventing/slowing down hearing loss and not by reversing hearing loss already occurred. This information is essential in the design of any clinical trial.

The cochlear phenotype progression is as described in our recent study of the early cochlear pathology of this *Ndp*-KO mouse model (Bryant, Pauzuolyte et al., 2022), and the retinal phenotype has been described by others previously (Luhmann, Lin et al., 2005, Richter, Gottanka et al., 1998).

We have summarized the pathological changes in the *Ndp*-KO mice in Fig 1F showing our study design. We have now also provided additional information on the phenotype timepoints in the introduction by inserting the following sentences on page 3.

"Vascular morphological abnormalities were apparent in the spiral ligament and stria vascularis as early as P10. Loss of cochlear vascular barrier was detected at P20, and reduction of endocochlear potential by 1 month. "

We have also now included an example of an organ of Corti at P30 showing the onset of OHC death (Fig S2E-F) and examples of the retinal vessel pathology at P2 and P21. These examples represent pathological events that characteristically distinguish the three treatment timepoints.

2. The significant conclusions should include 1). Systemic delivery at early stage results in the rescue of vision and hearing; 2). The time point used in mice may not be applicable to humans due to the difference in the development of ears and eyes and the onset of blindness and hearing loss; 3). Local delivery may be advantageous for humans. The study did not characterize other organs/tissues that AAV9 targets. The expression of NDP in unrelated tissues may cause safety concerns over time. Beyond the safety feature, local delivery allows testing a range of virus doses.

We thank the Reviewer for these suggestions, which we have used to prepare the below "Paper Explained Section" as required by the journal:

"PROBLEM: Norrie disease is a devastating genetic disorder that causes dual vision and hearing lossin patients without treatment. The study aims to develop a gene replacement therapy for Norrie disease using a mouse model and lay the groundwork for future application in patients.

RESULTS: 1) Systemic treatment at an early stage (neonates) resulted in the rescue of vision and hearing, but may not be translatable to humans due to the differences in the development of ears and eyes and the onset of blindness and hearing loss; 2) Treatment at later stages in mice, equivalent to treatment of children and young adults, was not efficient for rescue of retinal dysfunction, but showed efficacy in significantly improving the outcomes of the progressive hearing deterioration . 3) Vascular barrier abnormalities in the retina and inner ear were at least partially responsive to treatment across the different stages of the disease.

IMPACT: This study demonstrates that *NDP* gene therapy could be a viable approach to prevent the progression of hearing loss in a genetic deafblindness syndrome, Norrie disease. The efficacy of the therapy after the onset of degenerative changes in the cochlea and in improvement of the vascular barrier in eye and ear strongly supports continuing the effort towards the clinic. "

We have included the concerns about the safety and the benefits of local delivery in the Discussion (Page 15 and 18) (also see the question 3):

"As systemic delivery of AAV risks side effects, therefore direct, local delivery to the eye and ear may be more suitable for clinical translation, allow dosage optimization, and enable higher local levels of transduction."

3. One major limitation of the study is a relatively short time window post injection. In most gene therapy studies, the results tend to be more effective at the early stage, but the effects diminish over time. As NDP manifests as late onset progressive hearing loss, the long term outcome will be important to evaluate how sustained the treatment is. Please discuss the limitation of the current study.

We agree with the Reviewer's point that the relatively short duration of the study (3 months) is a limitation and have added these points to the discussion on page 18

Page 18 "A limitation of the current study is that the treated mice were followed post injection up to a maximum of 3 months of age. During which time no adverse health effects resulting from the treatment were observed. As Norrie disease manifests as late onset progressive hearing loss, the long-term outcome will be important to evaluate how sustained the treatment is and whether the effects diminish over time. After the later interventions (P21, P30), less cochlear cells were transduced and transgene expression was lower. It is possible that lower efficacy at later treatment timepoints is due to insufficient delivery to the target cells in mature animals and/or low responsiveness of aspects of the pathology already existing at the time of treatment. As systemic delivery of AAV risks side effects, therefore direct, local delivery to the eye and ear may be more suitable for clinical translation, allow dosage optimization, and enable higher local levels of transduction."

4. It will be helpful to present a figure to show the expression of Ndp in the retina and cochlea, so readers can compare it with the AAV-mediated Ndp expression. I am not clear if the cells transduced by the AAV are the same Ndp expressing cells or there are some differences. This information will help understand the rescue effect.

We agree that this is an important mechanistic point and have now performed additional analyses of scRNAseq datasets presented in new Fig S3 to help understand the rescue effect along with additional analysis of NDP/GFP proteins by immunostainings of the tissue (New Fig EV2 and Fig EV3 and new Fig 3). These data are presented and described in new section "NDP immunohistochemical analysis in the retina and cochlea" on pages 7-8.

These analyses suggest that there are some differences in the sites of AAV-mediated NDP expression and the endogenous Ndp expression sites.

On page 6 we state "Retinal ganglion cells were efficiently transduced in early or late treated mice whereas expression in Müller glial cells, a physiological site of *Ndp* expression (Ye, Wang et al., 2009) was rare (Fig 2B-C)."

As we state in the discussion page 15 "Rescue via our ubiquitous CAG promoter driven NDP construct implies that precise targeting of sites of NDP expression or OHCs is not necessary, so long as secreted NDP can reach the necessary target cells. This is consistent with rescue achieved in previous reports via ectopic overexpression of *Ndp* in the lens of transgenic mice (Ohlmann, Scholz et al., 2005).

5. The RNAseq study is informative as it shows the rescue effect on the molecular level by restoring gene expression deficient in the Ndp model. The confirmation by RT-PCR correlates well with the rescue, i.e. P2-L injection restored downstream genes more robustly, which leads to better functional recovery. Please discuss the point.

We have added this important point to the discussion on page 14.

"The RNAseq study was informative as it showed the rescue effect at the molecular level by restoring gene expression deficient in the *Ndp*-KO model. The confirmation by qRT-PCR correlated well with the rescue, i.e. P2-L injection restored downstream gene expression robustly, which was associated with better functional outcomes."

6. P9, 2nd paragraph, the description is not accurate. Judging by RT-PCR, P2 injection better recapitulated the expression level of genes (Fig4E, F, G, H, I). This data is informative as it may predicate the final outcome of the treatment, which is the best by P2 intervention. Please re-write the paragraph as it leaves people with the impression that later interventions work just well as early intervention.

We agree with the reviewer's observation, and we edited the text on page 12 to emphasise this point as follows:

qRT-PCR confirmed significant differential expression between *Ndp*-KO and WT for nine genes; *Plvap*, *Clu*, *Ceacam16, Nr1h4* were upregulated in the *Ndp*-KO*; Abcb1a*, *Cldn5*, *Slc7a1, Slc7a5* and *Sox17* were downregulated; (Fig 4B-J, two-way ANOVA with Tukey's *post hoc* test $p < 0.05$). At 2 months, disease biomarker gene expression returned to WT expression levels in the neonatal P2-L and juvenile P21-H treatment groups (all nine genes), similarly in the young adult P30-H group (all except *Cldn5, Clu, Nr1h4*), while the low dose P21-L treatment was less effective (Fig 5B-J) (blue, ns, indicating gene expression showing no significant difference from WT in each treatment group, and red asterisks, showing significant difference from the *Ndp*-KO).

These data indicate that dysregulated gene expression levels found in the *Ndp*-KO was restored to that of the WT, not only after neonatal treatment, but also after later treatment of juvenile and young adult mice, at later stages of the pathology. Overall P2 injection better recapitulated the WT expression level of genes (Fig 4E-I) (see red asterisks indicating significant difference from *Ndp*-KO, and no significant difference from WT, blue, ns). These patterns of rescue of gene dysregulation are in line with the levels of GFP transduction and transgene expression in the cochlea (Fig 3) whereby the highest levels of cochlea transduction were shown after treatment at P2. Since several of these genes are biomarkers for cochlear microvascular pathology, our results suggest that delivery of NDP by gene therapy may maintain and restore cochlear barrier and transport function.

7. P4, "with anti-FLAG immunostaining on the cell surface (yellow, Fig 1B-B')", the color should be red, not yellow.

We have corrected the sentence as follows:

"Fig 1B shows cytoplasmic EGFP (green) in the transfected HEK293 cells that are colabeled with anti-FLAG (red) immunostaining on the cell surface (co-localisation yellow, Fig 1B-B'')."

8. I don't see much labeling of GFP in the SGNs in the P30-H group. As the result, the statement" Spiral ganglion neurons were transduced in all treatment groups (Fig 2E, Fig S4 A-E)" should be rewritten.

We have re-written this section on page 6 as follows:

The spiral ganglia region was transduced as well as the lateral wall and modiolus (Fig2F-I, Fig EV1A-E). Transduction appeared higher after neonatal administration compared with treatment in juveniles and young adults (Fig 2F-I, Fig EV1A-E). We also added in immunofluorescence analysis of higher magnification cryosections of the spiral ganglia region which shows co-labelling of TUBB3 labelled neurons and GFP transduced cells in the P2-L treated *Ndp*-KO cochlea (Fig EV3).

9. Judging by GFP labeling, transduction at p2-L and p21-H seems to be efficient in targeting the lateral wall, but not at p30, even at a high dose.

We added this point on page 6:

"GFP labelling showed that lateral wall transduction was efficient in the P2-L and P21-H group, but not in the P30-H group."

10. Fig~~1~~E, explain the arrows. Are they SGNs? Should do double labeling with TuJ1 to show GFP+ cells are neurons. Looks like other cells, in addition to SGNs, are also GFP+.

Please see answer to point 8 above. We have confirmed TUBB3 (TuJ1) labelled SGNs are transduced. Please see new Fig EV3.

11. In later interventions, the number of cells transduced is fewer, and the expression of the transgene is lower. This could be due to insufficient delivery to the target cells in mature animals. It will be important to perform a comparative study in the future by local delivery, compare the result with the current study, and decide a possible route for human study.

We agree with the reviewer and added these points to the Discussion (Page 18):

After the later interventions (P21, P30), less cochlear cells were transduced and transgene expression was lower. It is possible that lower efficacy at later treatment timepoints is due to insufficient delivery to the target cells in mature animals and/or low responsiveness of aspects of the pathology already existing at the time of treatment. As systemic delivery of AAV risks side effects, direct delivery to the eye and ear may be more suitable for clinical translation, allow dosage optimization, and enable higher local levels of transduction. It will be important to perform a comparative study in the future by local delivery, compare the result with the current study, and decide a possible route for human study."

REFERENCES

Bryant D, Pauzuolyte V, Ingham NJ, Patel A, Pagarkar W, Anderson LA, Smith KE, Moulding DA, Leong YC, Jafree DJ, Long DA, Al-Yassin A, Steel KP, Jagger DJ, Forge A, Berger W, Sowden JC, Bitner-Glindzicz M (2022) The timing of auditory sensory deficits in Norrie disease has implications for therapeutic intervention. *JCI insight* 7

Eilken HM, Diéguez-Hurtado R, Schmidt I, Nakayama M, Jeong H-W, Arf H, Adams S, Ferrara N, Adams RH (2017) Pericytes regulate VEGF-induced endothelial sprouting through VEGFR1. *Nature communications* 8: 1574

Flotte TR, Buning H (2018) Severe Toxicity in Nonhuman Primates and Piglets with Systemic High-Dose Administration of Adeno-Associated Virus Serotype 9-Like Vectors: Putting Patients First. *Hum Gene Ther* 29: 283-284

He F, Li J, Mu Y, Kuruba R, Ma Z, Wilson A, Alber S, Jiang Y, Stevens T, Watkins S, Pitt B, Xie W, Li S (2006) Downregulation of endothelin-1 by farnesoid X receptor in vascular endothelial cells. *Circ Res* 98: 192-9

Hinderer C, Katz N, Buza EL, Dyer C, Goode T, Bell P, Richman LK, Wilson JM (2018) Severe Toxicity in Nonhuman Primates and Piglets Following High-Dose Intravenous Administration of an Adeno-Associated Virus Vector Expressing Human SMN. *Hum Gene Ther* 29: 285-298

Iwagaki T, Suzuki T, Nakashima T (2000) Development and regression of cochlear blood vessels in fetal and newborn mice. *Hear Res* 145: 75-81

Kuzmin DA, Shutova MV, Johnston NR, Smith OP, Fedorin VV, Kukushkin YS, van der Loo JCM, Johnstone EC (2021) The clinical landscape for AAV gene therapies. *Nat Rev Drug Discov* 20: 173-174

Lee S, Shin JO, Sagong B, Kim UK, Bok J (2017) Spatiotemporal expression patterns of clusterin in the mouse inner ear. *Cell Tissue Res* 370: 89-97

Luhmann UFO, Lin J, Acar N, Lammel S, Feil S, Grimm C, Seeliger MW, Hammes H-P, Berger W (2005) Role of the Norrie disease pseudoglioma gene in sprouting angiogenesis during development of the retinal vasculature. *Investigative ophthalmology & visual science* 46: 3372-3382

Massaro G, Hughes MP, Whaler SM, Wallom K-L, Priestman DA, Platt FM, Waddington SN, Rahim AA (2020) Systemic AAV9 gene therapy using the synapsin I promoter rescues a mouse model of neuronopathic Gaucher disease but with limited cross-correction potential to astrocytes. *Human molecular genetics* 29: 1933-1949

Mattar CN, Wong AM, Hoefer K, Alonso-Ferrero ME, Buckley SM, Howe SJ, Cooper JD, Waddington SN, Chan JK, Rahim AA (2015) Systemic gene delivery following intravenous administration of AAV9 to fetal and neonatal mice and late-gestation nonhuman primates. *FASEB J* 29: 3876-88

Ohlmann A, Scholz M, Goldwich A, Chauhan BK, Hudl K, Ohlmann AV, Zrenner E, Berger W, Cvekl A, Seeliger MW, Tamm ER (2005) Ectopic norrin induces growth of ocular capillaries and restores normal retinal angiogenesis in Norrie disease mutant mice. *The Journal of neuroscience : the official journal of the Society for Neuroscience* 25: 1701-1710

Rehm HL, Zhang D-S, Brown MC, Burgess B, Halpin C, Berger W, Morton CC, Corey DP, Chen Z-Y (2002) Vascular defects and sensorineural deafness in a mouse model of Norrie disease. *The Journal of neuroscience : the official journal of the Society for Neuroscience* 22: 4286-4292

Richter M, Gottanka J, May CA, Welge-Lüssen U, Berger W, Lütjen-Drecoll E (1998) Retinal vasculature changes in Norrie disease mice. *Investigative ophthalmology & visual science* 39: 2450-2457

Rohne P, Prochnow H, Koch-Brandt C (2016) The CLU-files: disentanglement of a mystery. *Biomol Concepts* 7: 1-15

Saito T, Zhang ZJ, Tokuriki M, Ohtsubo T, Shibamori Y, Yamamoto T, Saito H (2001) Cyclosporin A inhibits the extrusion pump function of p-glycoprotein in the inner ear of mice treated with vinblastine and doxorubicin. *Brain Res* 901: 265-70

Schafer NF, Luhmann UF, Feil S, Berger W (2009) Differential gene expression in Ndph-knockout mice in retinal development. *Invest Ophthalmol Vis Sci* 50: 906-16

Sharlin DS, Visser TJ, Forrest D (2011) Developmental and cell-specific expression of thyroid hormone transporters in the mouse cochlea. *Endocrinology* 152: 5053-64

Wang X, Zhang J, Li G, Sai N, Han J, Hou Z, Kachelmeier A, Shi X (2019) Vascular regeneration in adult mouse cochlea stimulated by VEGF-A165 and driven by NG2-derived cells ex vivo. *Hearing research* 377: 179-188

Yahyaoui R, Pérez-Frías J (2019) Amino Acid Transport Defects in Human Inherited Metabolic Disorders. *International journal of molecular sciences* 21

Ye X, Wang Y, Cahill H, Yu M, Badea TC, Smallwood PM, Peachey NS, Nathans J (2009) Norrin, frizzled-4, and Lrp5 signaling in endothelial cells controls a genetic program for retinal vascularization. *Cell* 139: 285-298

Zhang J, Hou Z, Wang X, Jiang H, Neng L, Zhang Y, Yu Q, Burwood G, Song J, Auer M, Fridberger A, Hoa M, Shi X (2021) VEGFA165 gene therapy ameliorates blood-labyrinth barrier breakdown and hearing loss. *JCI insight* 6

Zhang Z-J, Saito T, Kimura Y, Sugimoto C, Ohtsubo T, Saito H (2000) Disruption of mdr1a p-glycoprotein gene results in dysfunction of blood–inner ear barrier in mice. *Brain Research* 852: 116-126

Zheng J, Miller KK, Yang T, Hildebrand MS, Shearer AE, DeLuca AP, Scheetz TE, Drummond J, Scherer SE, Legan PK, Goodyear RJ, Richardson GP, Cheatham MA, Smith RJ, Dallos P (2011) Carcinoembryonic antigen-related cell adhesion molecule 16 interacts with alpha-tectorin and is mutated in autosomal dominant hearing loss (DFNA4). *Proc Natl Acad Sci U S A* 108: 4218-23

Zhou Y, Wang Y, Tischfield M, Williams J, Smallwood PM, Rattner A, Taketo MM, Nathans J (2014) Canonical WNT signaling components in vascular development and barrier formation. *J Clin Invest* 124: 3825-46

7th Jun 2023

Dear Prof. Sowden,

Thank you for the submission of your revised manuscript to EMBO Molecular Medicine. I am pleased to inform you that we will be able to accept your manuscript pending the following final amendments:

1) Figures: Please reduce the number of EV figures to max 5 (e.g. move 2 figures to Appendix).
2) In the main manuscript file, please do the following:
- Reduce keywords to max. 5.
- Add callouts for Figure 8F.
- Remove data not shown (p.17).
- Move Tables 1 and 2 to the end of the manuscript.
- In M&M, add statistical paragraph that should reflect all information that you have filled in the Authors Checklist, especially regarding randomization, blinding, replication.
- Please remove all Supplementary Table legends and add them to the corresponding table file (in separate tab in .xls files).
- Correct heading in the EV figure legends to Expanded Figure Legends.
- Please rename "Conflict of Interest" to "Disclosure Statement & Competing Interests". We updated our journal's competing interests policy in January 2022 and request authors to consider both actual and perceived competing interests. Please review the policy https://www.embopress.org/competing-interests and update your competing interests if necessary.
- Author contributions: Please remove it from the manuscript and specify author contributions in our submission system. CRediT has replaced the traditional author contributions section because it offers a systematic machine-readable author contributions format that allows for more effective research assessment. You are encouraged to use the free text boxes beneath each contributing author's name to add specific details on the author's contribution. More information is available in our guide to authors:
https://www.embopress.org/page/journal/17574684/authorguide#authorshipguidelines
- In data availability section we noticed that deposited RNA seq data are currently not accessible. Please be aware that all datasets should be made freely available upon acceptance, without restriction. Please check "Author Guidelines" for more information. https://www.embopress.org/page/journal/17574684/authorguide#availabilityofpublishedmaterial
- Correct the reference citation in the reference list. Where there are more than 10 authors on a paper, 10 will be listed, followed by "et al.". Please check "Author Guidelines" for more information.
https://www.embopress.org/page/journal/17574684/authorguide#referencesformat
3) Apendix: Please add page numbers and orrect nomenclature to Appendix Figure S1 etc., also in the main manuscript text.
4) Tables: Please rename Tables S1-3 to Dataset EV1-3 with their legends in separate tab and update their callouts in the main text.
5) Synopsis: Every published paper now includes a 'Synopsis' to further enhance discoverability. Synopses are displayed on the journal webpage and are freely accessible to all readers. They include separate synopsis image and synopsis text.
- Synopsis image: Please provide the synopsis image as a high-resolution jpeg file 550 px-wide x (250-400)-px high.
- Synopsis text: In addition to the short standfirst (maximum of 300 characters, including space) please provide 2-5 one sentence bullet points that summarise the paper as a .doc file. Please write the bullet points to summarise the key NEW findings. They should be designed to be complementary to the abstract - i.e. not repeat the same text. We encourage inclusion of key acronyms and quantitative information (maximum of 30 words / bullet point). Please use the passive voice.
- Please check your synopsis text and image before submission with your revised manuscript. Please be aware that in the proof stage minor corrections only are allowed (e.g., typos).
6) Source data: Please zipp all EV figure source data and upload as one file.
7) For more information: This space should be used to list relevant web links for further consultation by our readers. Could you identify some relevant ones and provide such information as well? Some examples are patient associations, relevant databases, OMIM/proteins/genes links, author's websites, etc...
8) As part of the EMBO Publications transparent editorial process initiative (see our Editorial at http://embomolmed.embopress.org/content/2/9/329), EMBO Molecular Medicine will publish online a Review Process File (RPF) to accompany accepted manuscripts. This file will be published in conjunction with your paper and will include the anonymous referee reports, your point-by-point response and all pertinent correspondence relating to the manuscript. Let us know whether you agree with the publication of the RPF and as here, if you want to remove or not any figures from it prior to publication. Please note that the Authors checklist will be published at the end of the RPF.
9) Please provide a point-by-point letter INCLUDING my comments as well as the reviewer's reports and your detailed responses (as Word file).

I look forward to reading a new revised version of your manuscript as soon as possible.

Yours sincerely,

Zeljko Durdevic

Zeljko Durdevic
Editor
EMBO Molecular Medicine

*** Instructions to submit your revised manuscript ***

*** PLEASE NOTE *** As part of the EMBO Publications transparent editorial process initiative (see our Editorial at https://www.embopress.org/doi/pdf/10.1002/emmm.201000094), EMBO Molecular Medicine will publish online a Review Process File to accompany accepted manuscripts.

In the event of acceptance, this file will be published in conjunction with your paper and will include the anonymous referee reports, your point-by-point response and all pertinent correspondence relating to the manuscript. If you do NOT want this file to be published, please inform the editorial office at contact@embomolmed.org.

When submitting your revised manuscript, please include:

1) a .docx formatted version of the manuscript text (including Figure legends and tables)

2) Separate figure files*

3) supplemental information as Expanded View and/or Appendix. Please carefully check the authors guidelines for formatting Expanded view and Appendix figures and tables at https://www.embopress.org/page/journal/17574684/authorguide#expandedview

4) a letter INCLUDING the reviewer's reports and your detailed responses to their comments (as Word file).

5) The paper explained: EMBO Molecular Medicine articles are accompanied by a summary of the articles to emphasize the major findings in the paper and their medical implications for the non-specialist reader. Please provide a draft summary of your article highlighting
- the medical issue you are addressing,
- the results obtained and
- their clinical impact.
This may be edited to ensure that readers understand the significance and context of the research.
Please refer to any of our published articles for an example.

6) For more information: There is space at the end of each article to list relevant web links for further consultation by our readers. Could you identify some relevant ones and provide such information as well? Some examples are patient associations, relevant databases, OMIM/proteins/genes links, author's websites, etc...

7) Author contributions: the contribution of every author must be detailed in a separate section.

8) EMBO Molecular Medicine now requires a complete author checklist (https://www.embopress.org/page/journal/17574684/authorguide) to be submitted with all revised manuscripts. Please use the checklist as guideline for the sort of information we need WITHIN the manuscript. The checklist should only be filled with page numbers were the information can be found. This is particularly important for animal reporting, antibody dilutions (missing) and exact values and n that should be indicted instead of a range.

9) Every published paper now includes a 'Synopsis' to further enhance discoverability. Synopses are displayed on the journal webpage and are freely accessible to all readers. They include a short stand first (maximum of 300 characters, including space) as well as 2-5 one sentence bullet points that summarise the paper. Please write the bullet points to summarise the key NEW findings. They should be designed to be complementary to the abstract - i.e. not repeat the same text. We encourage inclusion of key acronyms and quantitative information (maximum of 30 words / bullet point). Please use the passive voice. Please attach

these in a separate file or send them by email, we will incorporate them accordingly.

You are also welcome to suggest a striking image or visual abstract to illustrate your article. If you do please provide a jpeg file 550 px-wide x 400-px high.

10) A Conflict of Interest statement should be provided in the main text

11) Please note that we now mandate that all corresponding authors list an ORCID digital identifier. This takes <90 seconds to complete. We encourage all authors to supply an ORCID identifier, which will be linked to their name for unambiguous name identification.

Currently, our records indicate that the ORCID for your account is 0000-0003-0937-2479.

Please click the link below to modify this ORCID:
Link Not Available

12) The system will prompt you to fill in your funding and payment information. This will allow Wiley to send you a quote for the article processing charge (APC) in case of acceptance. This quote takes into account any reduction or fee waivers that you may be eligible for. Authors do not need to pay any fees before their manuscript is accepted and transferred to our publisher.

*Additional important information regarding Figures

Each figure should be given in a separate file and should have the following resolution:
Graphs 800-1,200 DPI
Photos 400-800 DPI
Colour (only CMYK) 300-400 DPI"

Figures are not edited by the production team. All lettering should be the same size and style; figure panels should be indicated by capital letters (A, B, C etc). Gridlines are not allowed except for log plots. Figures should be numbered in the order of their appearance in the text with Arabic numerals. Each Figure must have a separate legend and a caption is needed for each panel.

*Additional important information regarding figures and illustrations can be found at https://bit.ly/EMBOPressFigurePreparationGuideline. See also figure legend preparation guidelines: https://www.embopress.org/page/journal/17574684/authorguide#figureformat

The system will prompt you to fill in your funding and payment information. This will allow Wiley to send you a quote for the article processing charge (APC) in case of acceptance. This quote takes into account any reduction or fee waivers that you may be eligible for. Authors do not need to pay any fees before their manuscript is accepted and transferred to our publisher.

***** Reviewer's comments *****

Referee #1 (Comments on Novelty/Model System for Author):

I think this is a high quality study which is worthy of publication.

Referee #1 (Remarks for Author):

The authors have adequately addressed my previous concerns. It is my opinion that this revised manuscript is suitable for publication. Congratulations!

Referee #3 (Comments on Novelty/Model System for Author):

The revision addressed all my and others' concerns satisfactorily.

Referee #3 (Remarks for Author):

The revision addressed all my and others' concerns satisfactorily. This study will likely lead to the further development of treatment for Norrie disease. While the systemic approach may not be applicable to Norrie disease due to early retina disease manifestations, it has the potential to be useful for other diseases, notably some forms of Usher syndrome.

Zeljko Durdevic

Editor
EMBO Molecular Medicine

Dear Editor,

Re: Systemic gene therapy rescues retinal pathology and hearing loss in a model of Norrie disease

Authors: Valda Pauzuolyte, Aara Patel, James R. Wawrzynski, Neil J. Ingham, Yeh Chwan Leong, Maria Bitner-Glindzicz, Wolfgang Berger, Simon Waddington, Karen P. Steel, Jane C. Sowden.

Thank you for the reviews of our revised manuscript submitted to EMBO Molecular Medicine. We are very pleased that the Reviewers were satisfied and that you will accept our manuscript pending the following final amendments. We have addressed all of these as detailed below.

Yours sincerely,

Jane Sowden

***** Reviewer's comments *****

Referee #1 (Comments on Novelty/Model System for Author):

I think this is a high quality study which is worthy of publication.

Referee #1 (Remarks for Author):

The authors have adequately addressed my previous concerns. It is my opinion that this revised manuscript is suitable for publication. Congratulations!

Referee #3 (Comments on Novelty/Model System for Author):

The revision addressed all my and others' concerns satisfactorily.

Referee #3 (Remarks for Author):

The revision addressed all my and others' concerns satisfactorily. This study will likely lead to the further development of treatment for Norrie disease. While the systemic approach may not be applicable to Norrie disease due to early retina disease manifestations, it has the potential to be useful for other diseases, notably some forms of Usher syndrome.

Editors comments
1) Figures: Please reduce the number of EV figures to max 5 (e.g. move 2 figures to Appendix).

EV2 and EV3 were moved to the appendix as S3 and S4 respectively. Figure references and legends have been amended accordingly.

2) In the main manuscript file, please do the following:

- Reduce keywords to max. 5.

This had been done

- Add callouts for Figure 8F.

Figure 8F (p. 13) was referred to in the text as Figure 7F. This has now been corrected.

- Remove data not shown (p.17).

This has now been removed (on p. 14)

- Move Tables 1 and 2 to the end of the manuscript.

Tables 1 and 2 have been moved to the end of the manuscript (before figure legends)

- In M&M, add statistical paragraph that should reflect all information that you have filled in the Authors Checklist, especially regarding randomization, blinding, replication.

This has been added on p. 20

- Please remove all Supplementary Table legends and add them to the corresponding table file (in separate tab in .xls files).

This had been done

- Correct heading in the EV figure legends to Expanded Figure Legends.

This has been done

- Please rename "Conflict of Interest" to "Disclosure Statement & Competing Interests". We updated our journal's competing interests policy in January 2022 and request authors to consider both actual and perceived competing interests. Please review the policy https://www.embopress.org/competing-interests and update your competing interests if necessary.

This has been renamed

- Author contributions: Please remove it from the manuscript and specify author contributions in our submission system. CRediT has replaced the traditional author contributions section because it offers a systematic machine-readable author contributions format that allows for more effective research assessment. You are encouraged to use the free text boxes beneath each contributing author's name to add specific details on the author's contribution. More information is available in our guide to authors:
https://www.embopress.org/page/journal/17574684/authorguide#authorshipguidelines

These have been removed and entered in the submission system.

- In data availability section we noticed that deposited RNA seq data are currently not accessible. Please be aware that all datasets should be made freely available upon acceptance, without restriction. Please check "Author Guidelines" for more information. https://www.embopress.org/page/journal/17574684/authorguide#availabilityofpublishedmaterial

We will set the Array express data E-MTAB-12703 release date to coincide with the embargo lifting /publication date.

- Correct the reference citation in the reference list. Where there are more than 10 authors on a paper, 10 will be listed, followed by "et al.". Please check "Author Guidelines" for more information. https://www.embopress.org/page/journal/17574684/authorguide#referencesformat
This has been done

3) Appendix: Please add page numbers and correct nomenclature to Appendix Figure S1 etc., also in the main manuscript text.

This has been done

4) Tables: Please rename Tables S1-3 to Dataset EV1-3 with their legends in separate tab and update their callouts in the main text.

This has been done

5) Synopsis: Every published paper now includes a 'Synopsis' to further enhance discoverability. Synopses are displayed on the journal webpage and are freely accessible to all readers. They include separate synopsis image and synopsis text.
- Synopsis image: Please provide the synopsis image as a high-resolution jpeg file 550 px-wide x (250-400)-px high.
- Synopsis text: In addition to the short standfirst (maximum of 300 characters, including space) please provide 2-5 one sentence bullet points that summarise the paper as a .doc file. Please write the bullet points to summarise the key NEW findings. They should be designed to be complementary to the abstract - i.e. not repeat the same text. We encourage inclusion of key acronyms and quantitative information (maximum of 30 words / bullet point). Please use the passive voice.
- Please check your synopsis text and image before submission with your revised manuscript. Please be aware that in the proof stage minor corrections only are allowed (e.g., typos).

Synopsis image

This image file has been uploaded.

Standfirst (maximum of 300 characters, including space)

Norrie disease is a genetic condition causing blindness and progressive deafness. Successful AAV mediated gene augmentation therapy in a mouse model showed that the Norrie phenotype is responsive to treatment after the onset of degeneration, preventing further progression of hearing loss.

Bullet points

- Injection of AAV9 NDP gene therapy in neonatal *Ndp*-KO mice prevented retinal dysfunction and hearing loss in adult mice and rescued retinal and cochlea vasculature abnormalities.
- Treatment of older *Ndp*-KO mice also preserved hearing by preventing the loss of sensory hair cells in the cochlea.
- RNAseq analyses showed that dysregulated gene expression patterns in the *Ndp*-KO cochlea were normalised by AAV9 NDP gene therapy.

6) Source data: Please zipp all EV figure source data and upload as one file.

Source data has been uploaded as separate zipp EV files because of file size. Sub-folders have been renamed to reflect the updated manuscript (maximum 5 EV figures)

7) For more information: This space should be used to list relevant web links for further consultation by our readers. Could you identify some relevant ones and provide such information as well? Some examples are patient associations, relevant databases, OMIM/proteins/genes links, author's websites, etc...

We have added relevant websites.

8) As part of the EMBO Publications transparent editorial process initiative (see our Editorial at http://embomolmed.embopress.org/content/2/9/329), EMBO Molecular Medicine will publish online a Review Process File (RPF) to accompany accepted manuscripts. This file will be published in conjunction with your paper and will include the anonymous referee reports, your point-by-point response and all pertinent correspondence relating to the manuscript. Let us know whether you agree with the publication of the RPF and as here, if you want to remove or not any figures from it prior to publication. Please note that the Authors checklist will be published at the end of the RPF.

We agree with the publication of the RPF.

9) Please provide a point-by-point letter INCLUDING my comments as well as the reviewer's reports and your detailed responses (as Word file).

This has been provided.

25th Jul 2023

Dear Prof. Sowden,

We are pleased to inform you that your manuscript is accepted for publication and is now being sent to our publisher to be included in the next available issue of EMBO Molecular Medicine.

Please read below for additional IMPORTANT information regarding your article, its publication and the production process.

Congratulations on your interesting work,

Zeljko Durdevic

Zeljko Durdevic
Editor
EMBO Molecular Medicine

Follow us on Twitter @EmboMolMed
Sign up for eTOCs at embopress.org/alertsfeeds

***** Reviewer's comments *****

*** *** *** IMPORTANT INFORMATION *** *** ***

SPEED OF PUBLICATION
The journal aims for rapid publication of papers, using using the advance online publication "Early View" to expedite the process: A properly copy-edited and formatted version will be published as "Early View" after the proofs have been corrected. Please help the Editors and publisher avoid delays by providing e-mail address(es), telephone and fax numbers at which author(s) can be contacted.

Should you be planning a Press Release on your article, please get in contact with embomolmed@wiley.com as early as possible, in order to coordinate publication and release dates.

LICENSE AND PAYMENT:

All articles published in EMBO Molecular Medicine are fully open access: immediately and freely available to read, download and share.

EMBO Molecular Medicine charges an article processing charge (APC) to cover the publication costs. You, as the corresponding author for this manuscript, should have already received a quote with the article processing fee separately. Please let us know in case this quote has not been received.

Once your article is at Wiley for editorial production you will receive an email from Wiley's Author Services system, which will ask you to log in and will present you with the publication license form for completion. Within the same system the publication fee can be paid by credit card, an invoice, pro forma invoice or purchase order can be requested.

Payment of the publication charge and the signed Open Access Agreement form must be received before the article can be published online.

PROOFS

You will receive the proofs by e-mail approximately 2 weeks after all relevant files have been sent o our Production Office. Please return them within 48 hours and if there should be any problems, please contact the production office at embopressproduction@wiley.com.

Please inform us if there is likely to be any difficulty in reaching you at the above address at that time. Failure to meet our deadlines may result in a delay of publication.

All further communications concerning your paper proofs should quote reference number EMM-2023-17393-V3 and be directed to the production office at embopressproduction@wiley.com.

Thank you,

Zeljko Durdevic
Editor
EMBO Molecular Medicine

EMBO Press Author Checklist

Corresponding Author Name: Jane Sowden

Journal Submitted to: EMBO Molecular Medicine
Manuscript Number: EMM-2023-17393

USEFUL LINKS FOR COMPLETING THIS FORM
The EMBO Journal - Author Guidelines
EMBO Reports - Author Guidelines
Molecular Systems Biology - Author Guidelines
EMBO Molecular Medicine - Author Guidelines

Reporting Checklist for Life Science Articles (updated January
This checklist is adapted from Materials Design Analysis Reporting (MDAR) Checklist for Authors. MDAR establishes a minimum set of requirements in transparent reporting in the life sciences (see Statement of Task: 10.31222/osf.io/9sm4x). Please follow the journal's guidelines in preparing your manuscript.
Please note that a copy of this checklist will be published alongside your article.

Abridged guidelines for figures
1. Data
The data shown in figures should satisfy the following conditions:
- the data were obtained and processed according to the field's best practice and are presented to reflect the results of the experiments in an accurate and unbiased manner.
- ideally, figure panels should include only measurements that are directly comparable to each other and obtained with the same assay.
- plots include clearly labeled error bars for independent experiments and sample sizes. Unless justified, error bars should not be shown for technical
- if n<5, the individual data points from each experiment should be plotted. Any statistical test employed should be justified.
- Source Data should be included to report the data underlying figures according to the guidelines set out in the authorship guidelines on Data

2. Captions
Each figure caption should contain the following information, for each panel where they are relevant:
- a specification of the experimental system investigated (eg cell line, species name).
- the assay(s) and method(s) used to carry out the reported observations and measurements.
- an explicit mention of the biological and chemical entity(ies) that are being measured.
- an explicit mention of the biological and chemical entity(ies) that are altered/varied/perturbed in a controlled manner.
- the exact sample size (n) for each experimental group/condition, given as a number, not a range;
- a description of the sample collection allowing the reader to understand whether the samples represent technical or biological replicates (including how many animals, litters, cultures, etc.).
- a statement of how many times the experiment shown was independently replicated in the laboratory.
- definitions of statistical methods and measures:
  - common tests, such as t-test (please specify whether paired vs. unpaired), simple χ2 tests, Wilcoxon and Mann-Whitney tests, can be unambiguously identified by name only, but more complex techniques should be described in the methods section;
  - are tests one-sided or two-sided?
  - are there adjustments for multiple comparisons?
  - exact statistical test results, e.g., P values = x but not P values < x;
  - definition of 'center values' as median or average;
  - definition of error bars as s.d. or s.e.m.

Please complete ALL of the questions below.
Select "Not Applicable" only when the requested information is not relevant for your study.

Materials

Newly Created Materials	Information included in the manuscript?	In which section is the information available? (Reagents and Tools Table, Materials and Methods, Figures, Data Availability Section)
New materials and reagents need to be available; do any restrictions apply?	Yes	Materials and Methods

Antibodies	Information included in the manuscript?	In which section is the information available? (Reagents and Tools Table, Materials and Methods, Figures, Data Availability Section)
For **antibodies** provide the following information:<br>- Commercial antibodies: RRID (if possible) or supplier name, catalogue number and or/clone number<br>- Non-commercial: RRID or citation	Yes	Materials and Methods

DNA and RNA sequences	Information included in the manuscript?	In which section is the information available? (Reagents and Tools Table, Materials and Methods, Figures, Data Availability Section)
**Short novel DNA or RNA including primers, probes**: provide the sequences.	Yes	Materials and Methods

Cell materials	Information included in the manuscript?	In which section is the information available? (Reagents and Tools Table, Materials and Methods, Figures, Data Availability Section)
**Cell lines:** Provide species information, strain. Provide accession number in repository **OR** supplier name, catalog number, clone number, and/**OR** RRID.	Yes	Materials and Methods
**Primary cultures:** Provide species, strain, sex of origin, genetic modification status.	Not Applicable	
Report if the cell lines were recently **authenticated** (e.g., by STR profiling) and tested for mycoplasma contamination.	Not Applicable	

Experimental animals	Information included in the manuscript?	In which section is the information available? (Reagents and Tools Table, Materials and Methods, Figures, Data Availability Section)
**Laboratory animals or Model organisms:** Provide species, strain, sex, age, genetic modification status. Provide accession number in repository **OR** supplier name, catalog number, clone number, **OR** RRID.	Yes	Materials and Methods
**Animal observed in or captured from the field:** Provide species, sex, and age where possible.	Not Applicable	
Please detail **housing and husbandry conditions**.	Yes	Materials and Methods

Plants and microbes	Information included in the manuscript?	In which section is the information available? (Reagents and Tools Table, Materials and Methods, Figures, Data Availability Section)
**Plants:** provide species and strain, ecotype and cultivar where relevant, unique accession number if available, and source (including location for collected wild specimens).	Not Applicable	
**Microbes:** provide species and strain, unique accession number if available, and source.	Not Applicable	

Human research participants	Information included in the manuscript?	In which section is the information available? (Reagents and Tools Table, Materials and Methods, Figures, Data Availability Section)
If collected and within the bounds of privacy constraints report on age, sex and gender or ethnicity for all study participants.	Not Applicable	

Core facilities	Information included in the manuscript?	In which section is the information available? (Reagents and Tools Table, Materials and Methods, Figures, Data Availability Section)
If your work benefited from core facilities, was their service mentioned in the acknowledgments section?	Yes	Acknowledgements

Design

Study protocol	Information included in the manuscript?	In which section is the information available? (Reagents and Tools Table, Materials and Methods, Figures, Data Availability Section)
If study protocol has been **pre-registered, provide DOI in the manuscript**. For clinical trials, provide the trial registration number **OR** cite DOI.	Not Applicable	
Report the **clinical trial registration number** (at ClinicalTrials.gov or equivalent), where applicable.	Not Applicable	

Laboratory protocol	Information included in the manuscript?	In which section is the information available? (Reagents and Tools Table, Materials and Methods, Figures, Data Availability Section)
Provide DOI OR other citation details if **external detailed step-by-step protocols** are available.	Not Applicable	

Experimental study design and statistics	Information included in the manuscript?	In which section is the information available? (Reagents and Tools Table, Materials and Methods, Figures, Data Availability Section)
Include a statement about **sample size** estimate even if no statistical methods were used.	Yes	Materials and Methods, statistics
Were any steps taken to minimize the effects of subjective bias when allocating animals/samples to treatment (e.g. **randomization procedure**)? If yes, have they been described?	Yes	Materials and Methods, statistics
Include a statement about **blinding** even if no blinding was done.	Yes	Materials and Methods, statistics
Describe **inclusion/exclusion criteria** if samples or animals were excluded from the analysis. Were the criteria pre-established?<br><br>If sample or data points were omitted from analysis, report if this was due to attrition or intentional exclusion and provide justification.	Yes	Materials and Methods, statistics
For every figure, are **statistical tests** justified as appropriate? Do the data meet the assumptions of the tests (e.g., normal distribution)? Describe any methods used to assess it. Is there an estimate of variation within each group of data? Is the variance similar between the groups that are being statistically compared?	Yes	Materials and Methods; Data availability Section

Sample definition and in-laboratory replication	Information included in the manuscript?	In which section is the information available? (Reagents and Tools Table, Materials and Methods, Figures, Data Availability Section)
In the figure legends: state number of times the experiment was **replicated** in laboratory.	Yes	Legends, Materials and Methods
In the figure legends: define whether data describe **technical or biological replicates**.	Yes	Legends, Materials and Methods

Ethics

Ethics	Information included in the manuscript?	In which section is the information available? (Reagents and Tools Table, Materials and Methods, Figures, Data Availability Section)
Studies involving **human participants**: State details of **authority granting ethics approval** (IRB or equivalent committee(s), provide reference number for approval.	Not Applicable	
Studies involving **human participants**: Include a statement confirming that **informed consent** was obtained from all subjects and that the experiments conformed to the principles set out in the WMA Declaration of Helsinki and the Department of Health and Human Services Belmont Report.	Not Applicable	
Studies involving **human participants:** For publication of **patient photos**, include a statement confirming that consent to publish was obtained.	Not Applicable	
Studies involving experimental **animals**: State details of **authority granting ethics approval** (IRB or equivalent committee(s), provide reference number for approval. Include a statement of compliance with ethical regulations.	Yes	Materials and Methods
Studies involving **specimen and field samples:** State if relevant **permits** obtained, provide details of authority approving study; if none were required, explain why.	Not Applicable	

Dual Use Research of Concern (DURC)	Information included in the manuscript?	In which section is the information available? (Reagents and Tools Table, Materials and Methods, Figures, Data Availability Section)
Could your study fall under dual use research restrictions? Please check biosecurity documents and list of **select agents and toxins** (CDC): https://www.selectagents.gov/sat/list.htm	Not Applicable	
If you used a select agent, is the security level of the lab appropriate and reported in the manuscript?	Not Applicable	
If a study is subject to dual use research of concern regulations, is the name of the **authority granting approval and reference number** for the regulatory approval provided in the manuscript?	Not Applicable	

Reporting

The MDAR framework recommends adoption of discipline-specific guidelines, established and endorsed through community initiatives. Journals have their own policy about requiring specific guidelines and recommendations to complement MDAR.

Adherence to community standards	Information included in the manuscript?	In which section is the information available? (Reagents and Tools Table, Materials and Methods, Figures, Data Availability Section)
State if relevant guidelines or checklists (e.g., **ICMJE, MIBBI, ARRIVE, PRISMA**) have been followed or provided.	Not Applicable	
For **tumor marker prognostic studies,** we recommend that you follow the **REMARK** reporting guidelines (see link list at top right). See author guidelines, under 'Reporting Guidelines'. Please confirm you have followed these guidelines.	Not Applicable	
For **phase II and III randomized controlled trials**, please refer to the **CONSORT** flow diagram (see link list at top right) and submit the CONSORT checklist (see link list at top right) with your submission. See author guidelines, under 'Reporting Guidelines'. Please confirm you have submitted this list.	Not Applicable	

Data Availability

Data availability	Information included in the manuscript?	In which section is the information available? (Reagents and Tools Table, Materials and Methods, Figures, Data Availability Section)
Have **primary datasets** been deposited according to the journal's guidelines (see 'Data Deposition' section) and the respective accession numbers provided in the Data Availability Section?	Yes	Data Availability Section
Were **human clinical and genomic datasets** deposited in a public access-controlled repository in accordance to ethical obligations to the patients and to the applicable consent agreement?	Not Applicable	
Are **computational models** that are central and integral to a study available without restrictions in a machine-readable form? Were the relevant accession numbers or links provided?	Not Applicable	
If publicly available data were reused, provide the respective **data citations in the reference list**.	Yes	Materials and Methods
